# Experimental evidence that stripes do not cool zebras

**DOI:** 10.1038/s41598-018-27637-1

**Published:** 2018-06-19

**Authors:** Gábor Horváth, Ádám Pereszlényi, Dénes Száz, András Barta, Imre M. Jánosi, Balázs Gerics, Susanne Åkesson

**Affiliations:** 1 Department of Biological Physics, ELTE Eötvös Loránd University, H-1117 Budapest, Pázmány sétány 1, Hungary; 2 Department of Zoology, Hungarian Natural History Museum, H-1083 Budapest, Ludovika tér 2-6, Hungary; 3 Estrato Research and Development Ltd., H-1124 Németvölgyi út 91/c, Budapest, Hungary; 4 Department of Physics of Complex Systems, ELTE Eötvös Loránd University, H-1117 Budapest, Pázmány sétány 1, Hungary; 5 Department of Anatomy and Histology, University of Veterinary Medicine, H-1078 Budapest, István utca 2, Hungary; 6 Department of Biology, Centre for Animal Movement Research, Lund University, Ecology Building, SE-223 62, Lund, Sweden

## Abstract

There are as many as 18 theories for the possible functions of the stripes of zebras, one of which is to cool the animal. We performed field experiments and thermographic measurements to investigate whether thermoregulation might work for zebra-striped bodies. A zebra body was modelled by water-filled metal barrels covered with horse, cattle and zebra hides and with various black, white, grey and striped patterns. The barrels were installed in the open air for four months while their core temperature was measured continuously. Using thermography, the temperature distributions of the barrel surfaces were compared to those of living zebras. The sunlit zebra-striped barrels reproduced well the surface temperature characteristics of sunlit zebras. We found that there were no significant core temperature differences between the striped and grey barrels, even on many hot days, independent of the air temperature and wind speed. The average core temperature of the barrels increased as follows: white cattle, grey cattle, real zebra, artificial zebra, grey horse, black cattle. Consequently, we demonstrate that zebra-striped coats do not keep the body cooler than grey coats challenging the hypothesis of a thermoregulatory role of zebra stripes.

## Introduction

The enigmatic role of the striking black and white stripe pattern of zebras has been the subject of vigorous discussions among researchers since Wallace^1,2^ and Darwin^3^. Until now, as many as 18 different explanations have been proposed for the possible functions of zebra stripes which can be combined into the following four major groups^4^:

1. anti-predation, including camouflage and various aspects of visual confusion^1,2,5–19^,
2. facilitating social interactions^14,17,20–23^,
3. thwarting the attack of biting flies^4,24–34^,
4. regulating body temperature^14,17,20,35,36^.

Hypothesis 3 has been experimentally tested and corroborated by field observations and experiments^4,25,26,29–32^. In this study, however, we test hypothesis 4 using field experiments.

According to hypothesis 4, zebra stripes are expected to cool the body by means of convective air eddies induced by temperature gradients over alternating black and white stripes. This hypothesis seems reasonable, because in sunshine the black zebra stripes are warmer due to their stronger absorption of sunlight compared to the cooler white stripes of higher reflectance^35^. Infrared photography of zebras showed that sunlit black stripes are warmer than sunlit white stripes and that the difference between them increases with rising air temperature. At night, however, temperature differences are reversed, with black stripes being cooler than white ones^37–39^. The infrared camera measurements of Caro^4^ did not confirm lower surface temperatures of sunlit zebras than sympatric sunlit ungulates (giraffe, impala and buffalo). On the other hand, data by Irondo and Rubenstein gathered with a laser infrared digital thermometer gun briefly mentioned by Larison *et al*.^36^ illustrated that zebras in sunshine might maintain significantly lower surface temperatures (29.2 °C) than similar-size sunlit hartebeests (32.5 °C) although these data have never been formally presented. Note, however, that surface temperatures do not represent the internal core temperature of extended bodies^4^, which is the most relevant parameter in the context of thermoregulation. The core temperature must not be higher than a critical value, otherwise the animal overheats which is lethal. Caro *et al*.^33^ matched annual mean air temperatures to striping across the 7 striped and unstriped equid species and 20 equid subspecies and found no correlation. Contrarily, Larison *et al*.^36^ matched air temperature variability to changes in striping across populations of plains zebras throughout Africa and found positive correlations between isothermality and mean temperature of the coldest season with striping intensity. Our present work sets out to resolve this discrepancy between previous studies.

**Figure 1:**
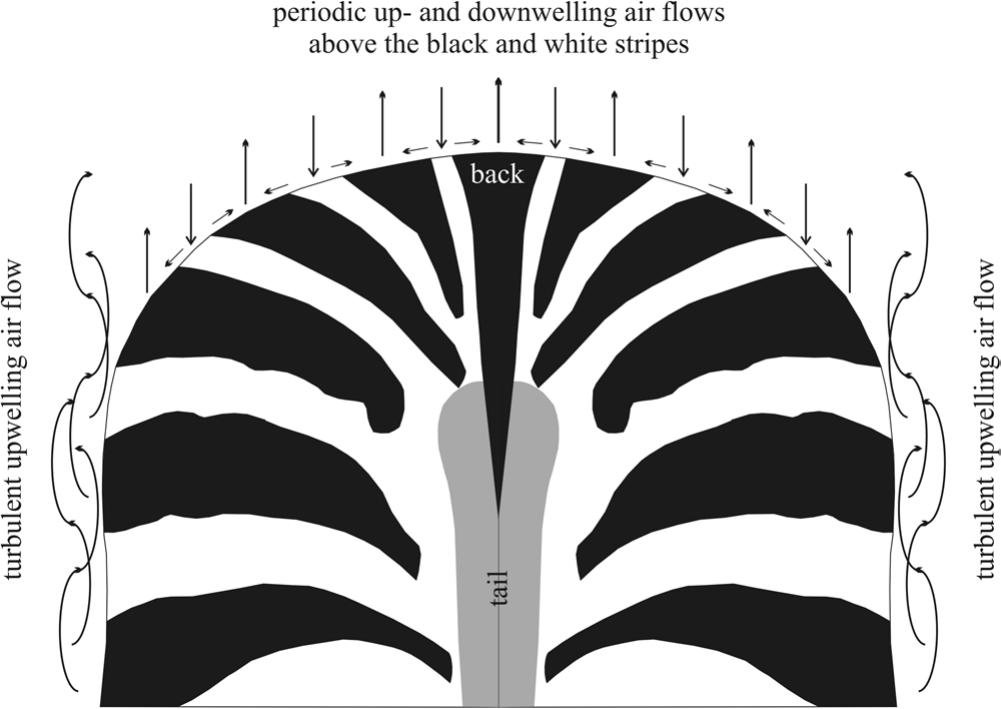
Hypothesized cooling by zebra stripes. According to the thermoregulation hypothesis, in sunshine the black zebra stripes are warmer than the white ones, thus in calm weather there may be upwelling and downwelling air streams above the former and latter stripes, respectively. Note that periodic up- and downwelling air streams (eddies) could form only above nearly horizontal region of the zebra body. At the tilted and vertical body surfaces the air flow might be rather chaotic, and can expected to be turbulent. The schematic striped pattern of a zebra back was drawn from a photograph taken by Gábor Horváth.

According to the hypothesis of thermoregulation, upwelling air streams may form over the warmer black stripes which are replaced by cooler air from the adjacent white stripes with downwelling air flows (Fig. 1). Consequently, convective air eddies might build up above periodic patterns of black and white stripes. In principle, such eddies might cool the zebra body in sunshine by transporting warm air away over the black stripes, and/or accelerating the evaporation of sweat on the zebra skin.

There are at least three problems with this hypothesis. (1) Until now, the formation of convective eddies over zebra bodies has not been documented. Principally, such buoyancy-driven eddies may be formed only over nearly horizontal parts of the striped body surface of zebras. Above strongly tilted or vertical parts of the body, such as the flanks, upwelling turbulent air flow could be formed parallel to the surface preventing the formation of periodic eddies (Fig. 1). Thus, considering possible thermoregulation under sunny and hot conditions, the use of stripes would be advantageous only along the nearly horizontal back zone of the body, while due to their strong light absorption, black stripes are definitely disadvantageous on the oblique and vertical side regions and legs. If zebras were striped to produce cooling air eddies, then they should only be striped on their dorsal surface. (2) The stability of convective eddies over horizontal surfaces is not known. Eddies could be easily blown off by weak local wind, which is practically always present in sunny weather. Furthermore, once the zebra moves, these eddies could be easily disrupted^4^. (3) It is unknown whether possible convective eddies above a perspiring horizontal surface of a striped coat could have a stronger cooling effect than cooling by perspiration over any monochrome coat.

**Figure 2:**
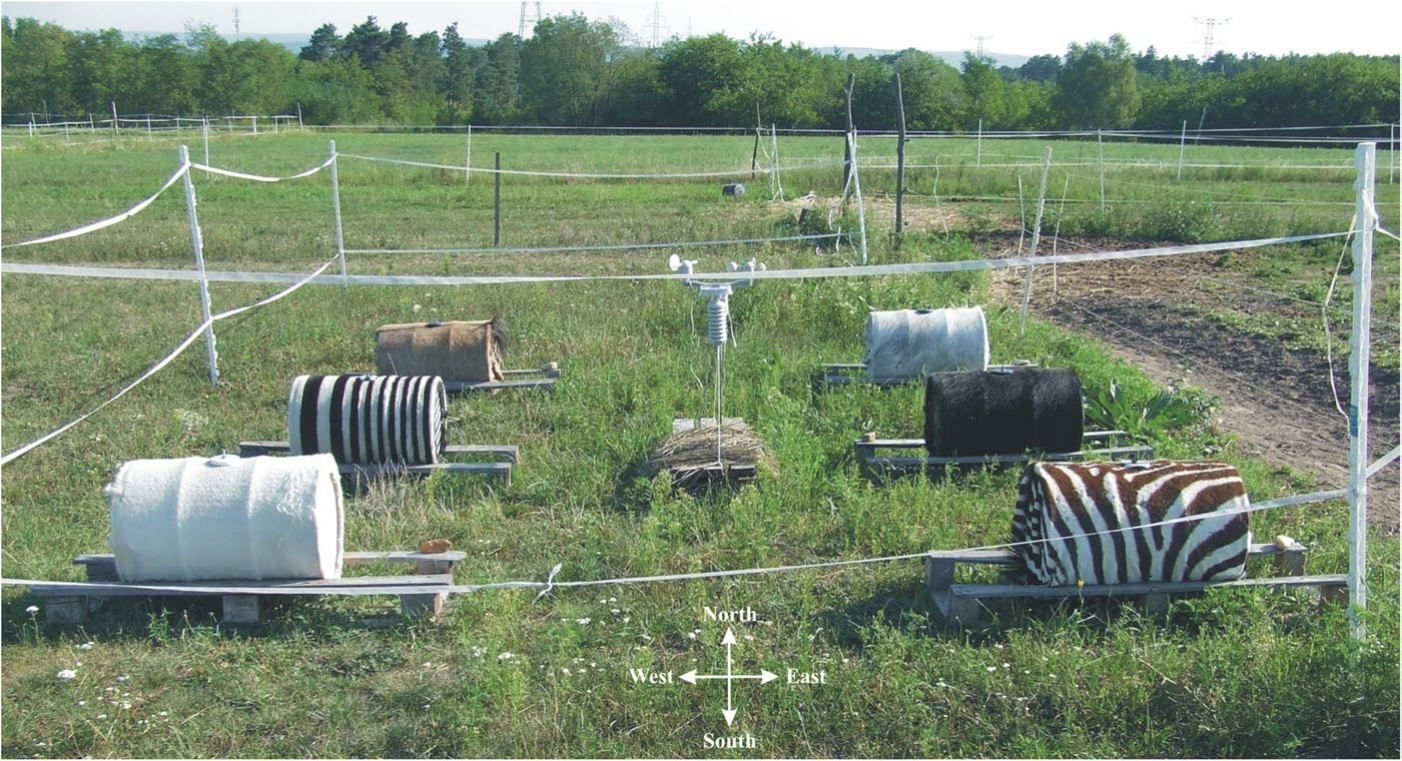
Experimental setup. Arrangement of the six barrels and the meteorological station used in the field experiments. The barrels were laid on a wooden holder which held them at a height of 10 cm above the grassy ground. A circular metal cup covered the hole of the barrel shell and kept the automatic thermometer for measuring the core temperature (photograph taken by Gábor Horváth).

Due to these open questions, the hypothesis of cooling by zebra stripes is physically and physiologically challenging. Black and white zebra stripes reflect sunlight quite differently, but the questions on the existence and mechanism of an overall cooling effect remained open until now. The aim of this work is to test experimentally the hypothesis of thermoregulation by means of thermography and thermophysical models of monochrome white, black, grey horses and striped zebras. Our thermophysical study tests the cooling hypothesis in direct field experiments (Fig. 2), and provides more convincing arguments against cooling by zebra stripes than previous studies that were either observational^4,37–39^ or correlative^33,36^. Our approach is based on measuring core temperatures of zebra models, rather than surface temperatures of zebras^4^.

## Results

### Air temperature and wind speed

Air temperature *T*_air_ (Supplementary Figs S1A–S4A) and wind speed *w* (Supplementary Figs S1B–S4B) were used as the most relevant control parameters of the core temperature *T*_core_ of our water-filled barrels. On each day of experiments 1–4, *T*_air_ increased after sunrise, culminated in early afternoon then decreased, and *w* had practically the same temporal pattern, as expected^40^. This strong positive correlation between the daily patterns of *T*_air_ and *w* (this is a well-known meteorological phenomenon also generalizable to Africa) decreases the possibility of cooling by convective eddies above zebra stripes: such cooling would be useful especially in hot weather when, however, the elevated wind speed and/or the motion of a zebra can easily blow away eddies.

Convective eddies can only form over the black and white stripes of the nearly horizontal regions of the body surface of zebras, if the temperature difference Δ*T*_b-w_ between adjacent black and white stripes is high enough, and *w* is low enough. It was not possible to measure Δ*T*_b-w_ continuously during our experiments. However, there is a strong positive correlation between *T*_air_ and Δ*T*_b-w_: the higher the former, the larger the latter. Thus, in order to estimate the frequency of possible convective eddies, we counted the number of situations when the weather conditions *T*_air_ > T* and *w* < w* occurred together during our field experiments. We found that the number of cases with low wind speeds (*w* < w* = 2 km/h) decreased with increasing T* (Supplementary Figs S5–S8). An enhanced cooling is beneficial in hot weather (*T* > 25 °C), however, above 25 °C predominantly high (*w* > 5 km/h) wind speeds occurred, that could not favour the formation of convective eddies. Nevertheless, a wind flow of elevated speed can cool zebras itself by increasing surface evaporation of sweat, thus stronger winds may be more effective for thermoregulation than the assumed convective air eddies above striped surfaces.

### Thermography of barrels

**Figure 3:**
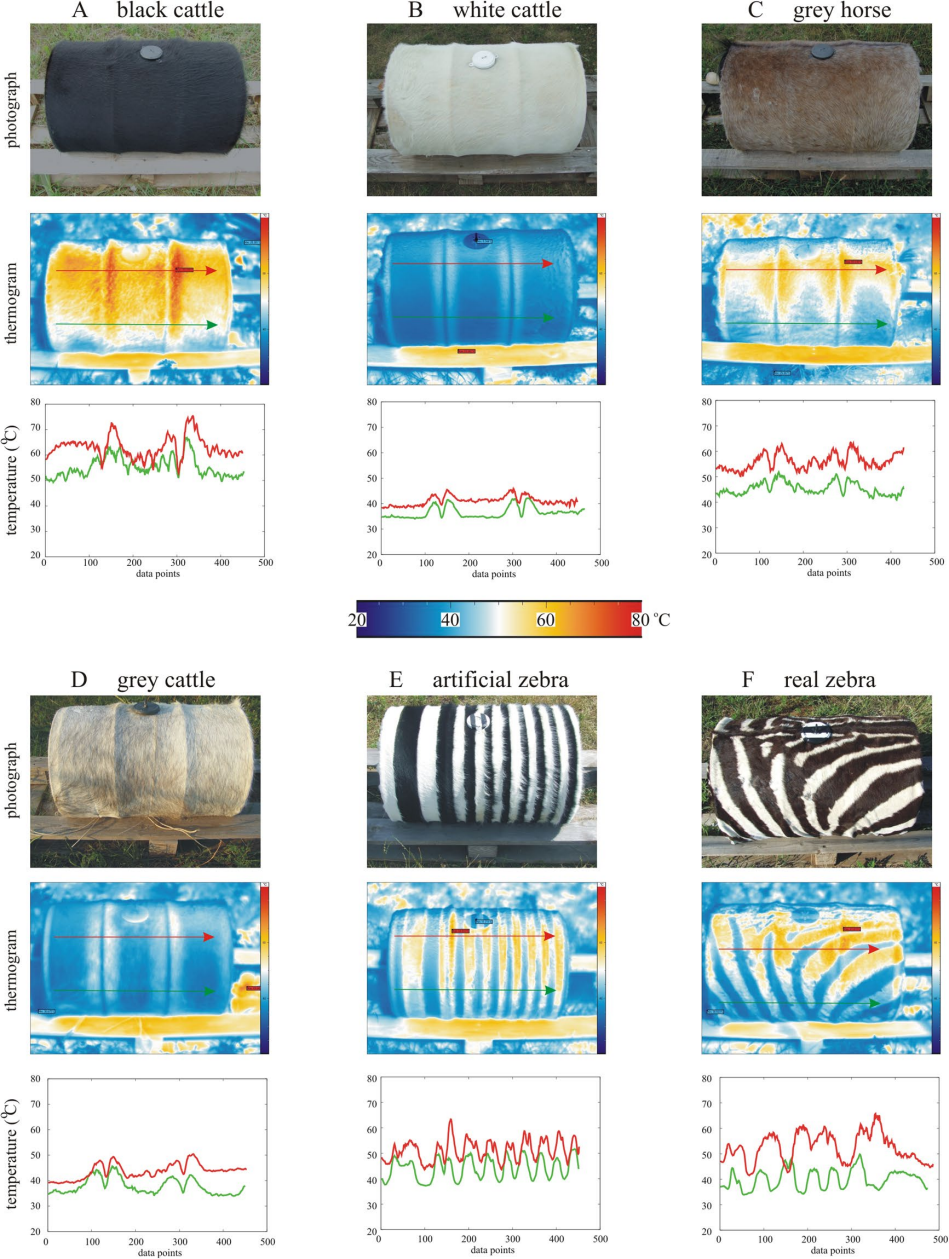
Thermography of barrels used in the experiments. Photographs (taken by Gábor Horváth) and thermograms of the six sunlit barrels covered by different hides (black cattle, white cattle, grey horse, grey cattle, artificial zebra, real zebra) used in our experiments 3 and 4, and the change of surface temperature *T* (°C) along straight lines marked with arrows in the thermograms. The upper (red) and lower (green) arrow runs on a sunlit and shady region of the barrel shell, respectively. The beginning and the end (head) of the arrows correspond to the first and last data points of the *T*-curves, respectively. In the thermograms red, yellow, white, light blue and dark blue colour hues code hot, warm, medium, cool and cold temperatures, respectively. In E and F, the large *T*-differences between adjacent black and white stripes are visible both in the thermograms and the *T*-curves. The photographs were taken independently of the thermograms.

**Figure 4:**
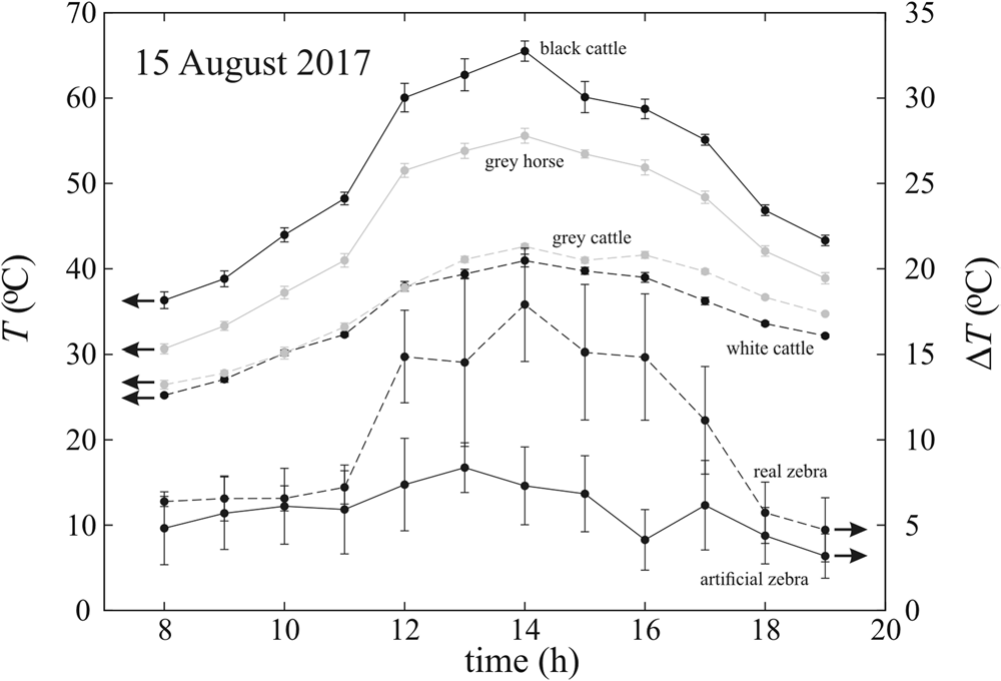
Surface temperature of barrels versus time. Average surface temperature *T* (°C, dots) ± standard deviation σ_T_ (°C, vertical bars) of water-filled barrels covered by homogeneous hides, and average surface temperature difference Δ*T* (°C, dots) between adjacent black and white stripes ± standard deviation σ_ΔT_ (°C, vertical bars) of water-filled barrels covered by zebra-striped hides as a function of time (hour = UTC + 2 h). The thermograms were measured on 15 August 2017 with a thermocamera, the temperature data of which were gathered along a straight line on the sunlit top of the barrels (numerical data are in Supplementary Table S3). Horizontal arrows show which *T* or Δ*T* vertical scale belongs to the corresponding curves.

The surface temperature *T* ± σ_T_ (average ± standard deviation) of the barrels covered by homogeneous hides and the surface temperature difference Δ*T*_b-w_ ± σ_ΔT_ between adjacent black and white stripes of the barrels covered by zebra-striped hides were recorded on 18 July, 30 July and 15 August 2017 with a thermocamera along a straight line on the sunlit barrels (Figs 3 and 4, Supplementary Fig. S9 and Tables S1–S3). The surface temperature *T* of the black, grey and white barrels increased tendentiously (i.e. not always monotonously) from 8 to 14–15 h (UTC + 2 h), and, after culminating, decreased tendentiously until 19 h. As expected, the white and black barrels were always the coldest and warmest, respectively, while *T* of the grey barrels was intermediate. Similarly, Δ*T*_b-w_ of the striped barrels tendentiously increased from 8 h, reached its maximum near 14–15 h, then decreased until 19 h. Δ*T*_b-w_ of the real zebra hide was usually larger than that of the artificial zebra hide. Between 11 and 17 h, the range of Δ*T*_b-w_ was 7–18 °C for the real, and 6–12 °C for the artificial zebra hide. We conclude from these measurements that the temperature differences Δ*T*_b-w_ between the black and white stripes were markedly high for six hours in sunny days. Hence, one of the prerequisites (high enough Δ*T*_b-w_) of convective air eddies was satisfied for these particular six hours.

**Figure 5:**
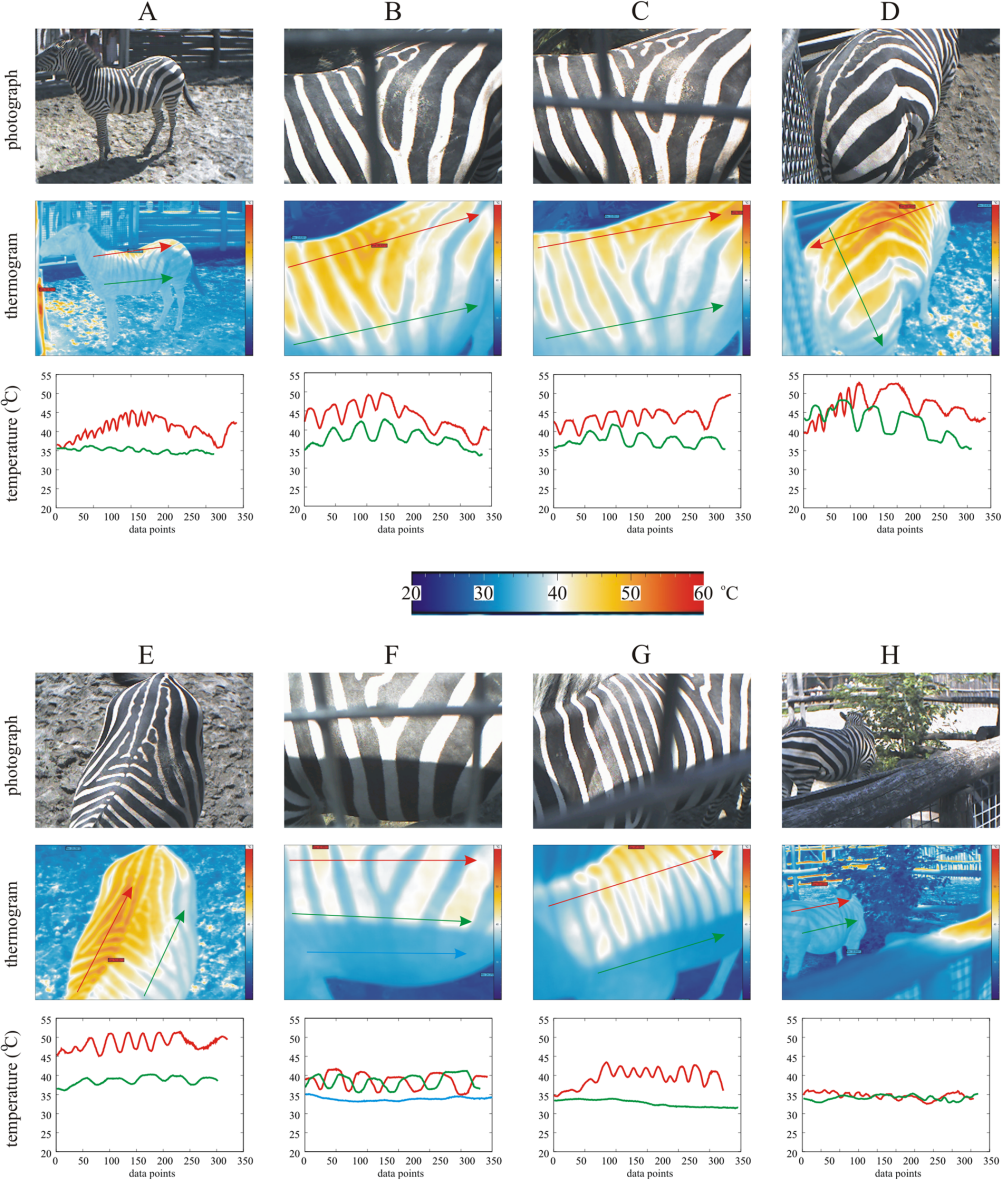
Thermography of zebras. Photographs (taken by Gábor Horváth) and thermograms of sunlit (**A**–**G**) and shady (**H**) zebras (*Equus burchelli boehmi*) in the Budapest Zoo & Botanical Garden, and the change of surface temperature *T* (°C) along straight lines marked with arrows in the thermograms. The upper (red) and lower (green) arrow runs on a sunlit and shady region of the zebra body, respectively. The beginning and the end (head) of the arrows correspond to the first and last data points of the *T*-curves. In the thermograms red, yellow, white, light blue and dark blue colour hues code hot, warm, medium, cool and cold temperatures, respectively. There are some small differences between the photograph and the thermogram of a given scene because of the slightly different fields of view of the optocamera and the thermocamera. Since the optocamera was immediately below the thermocamera, in scenes (**B**,**C**,**F** and **G**) the optocamera saw the (horizontal, vertical) metal rods of the fence, while these rods were out of the field of view of the thermocamera.

Figure 5 shows the photographs and thermograms of sunlit and shady zebras (*Equus burchelli boehmi*) in the Budapest Zoo & Botanical Garden and the temperature change along straight lines running on sunlit and shady regions of the zebra body. It is clear from Fig. 5 (see also Supplementary Table S4), that there are large temperature differences between neighbouring black and white zebra stripes only if they are illuminated by direct sunlight. In shady regions of the zebra body, such differences practically do not exist (Δ*T*_b-w_ < 0.2 °C).

From our thermographic studies (Figs 3–5, Supplementary Fig. S9 and Tables S1–S4) we conclude that the surface of our (sunlit/shady) striped barrels modelled well the temperature characteristics of the body surface of (sunlit/shady) living zebras.

### Reflection spectra of hides

The spectra of the white and black stripes of the artificial zebra hide were the same as that of the white and black cattle hide, respectively (Supplementary Fig. S10A), because the artificial zebra hide was created from white and black cattle hide stripes. The black stripes of the real zebra hide were dark brown of strongest reflectance in the wavelength range 600 nm < λ < 750 nm (Supplementary Fig. S10A). The spectrum of real white zebra stripes was very similar to that of the white cattle hide. The average spectrum of the real zebra hide was practically the same as that of the grey cattle, while the average spectrum of the artificial zebra hide was similar to that of these two former hides. The grey horse hide was slightly brownish with peak reflectance in the wavelength range 600 nm < λ < 650 nm (Supplementary Fig. S10A). If the whiteness of the white cattle hide is taken as unity (*wh* = 1), then the whiteness of the grey cattle, real zebra, artificial zebra, grey horse and black cattle was *wh* = 0.55, 0.53, 0.43, 0.25 and 0.03, respectively (Supplementary Fig. S10B, Table S5). Hence, the overall whiteness of the real zebra hide (0.53) was practically the same as that of the grey cattle hide (0.55), and it was brighter than the artificial zebra (0.43) and grey horse (0.25) hides.

### Time delay from cross correlation of air temperature and barrel’s core temperature

On the basis of correlations between air temperature *T*_air_(*t*) and the barrel’s core temperature *T*_core_(*t*) (Supplementary Fig. S11), we determined the time delay Δ*t* (thermal response time) on the warmest days (28 June, 20 July, 10 August, 1 September) of experiments 1–4 between *t*_min_ = 6:00 and *t*_max_ = 20:00 h (UTC + 2 h) for each barrel (Supplementary Fig. S12, Table S6). Depending on both the date (meteorological condition) of the experimental day and the type of the hide covering a barrel, Δ*t* ranged between 15 and 120 minutes, being minimal always for the black barrel. Due to this large variation of Δ*t*, our subsequent calculations were performed for Δ*t* = 0, 30, 60, 90 and 120 min.

### Comparison of the barrels’ core temperatures for all days

**Figure 6:**
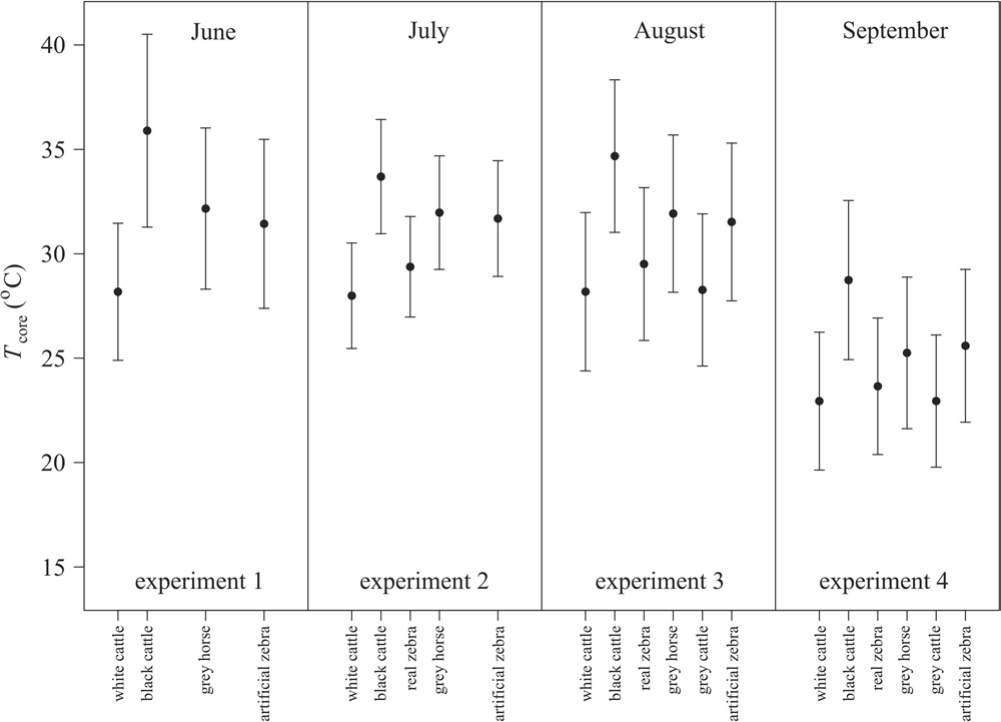
Core temperature of barrels on hot days. Average (dots) ± standard deviation (vertical bars) of the barrels’ core temperature *T*_core_ calculated only for hot days when the average air temperature was higher than 25 °C (above which cooling air eddies over zebras were the most likely to form) between 12:00 and 18:00 h (UTC + 2 h) in June (experiment 1), July (experiment 2) and August (experiment 3), and between 12:00 and 17:00 h in September (experiment 4).

Figure 6 (Supplementary Table S7) shows the average ± standard deviation of the barrels’ core temperature *T*_core_ for hot days with average air temperature >25 °C. (Supplementary Fig. S13 and Table S8 present the average ± standard deviation of the barrels’ core temperature *T*_core_ for all experimental days. Supplementary Fig. S14 and Table S9 display the mean air temperature on experimental days between 12:00 and 18:00 h June, July and August and between 12:00 and 17:00 h in September. In Supplementary Fig. S14, days above the horizontal line with average *T*_air_ > 25 °C are used to calculate the average and standard deviation for Fig. 6.) There were no statistically significant differences in the core temperatures between the barrels covered with grey horse, artificial zebra, real zebra and grey cattle hides (Supplementary Tables S10, S11). The core temperatures of barrels covered with black or white cattle hide differed statistically significantly from those of the homogeneous grey or zebra-striped barrels. As expected, *T*_core_ of the white barrel was always significantly lower than that of the black barrel. The statistically significant difference relations between *T*_core_ of barrels compared pairwise are summarized in Table 1.

**Table 1:** Statistically significant difference relations (< means: smaller than) between the core temperature *T*_core_ of barrels (w: white, b: black, gh: grey horse, az: artificial zebra, rz: real zebra, gc: grey cattle) compared pairwise for all days and for the hot days (with average *T*_air_ > 25 °C) of experiments 1–4.

comparison for all days	experiment 1	w < gh, w < az, gh < b, az < b
	experiment 2	w < gh, rz < b
	experiment 3	rz < b, gc < b
	experiment 4	gc < b
comparison for hot days	experiment 1	w < gh, w < az, gh < b, az < b
	experiment 2	w < gh, w < az, rz < b
	experiment 3	rz < b, gc < b
	experiment 4	rz < b, gc < b

### Comparison of the barrels’ core temperatures with T* and w* filtering

**Figure 7:**
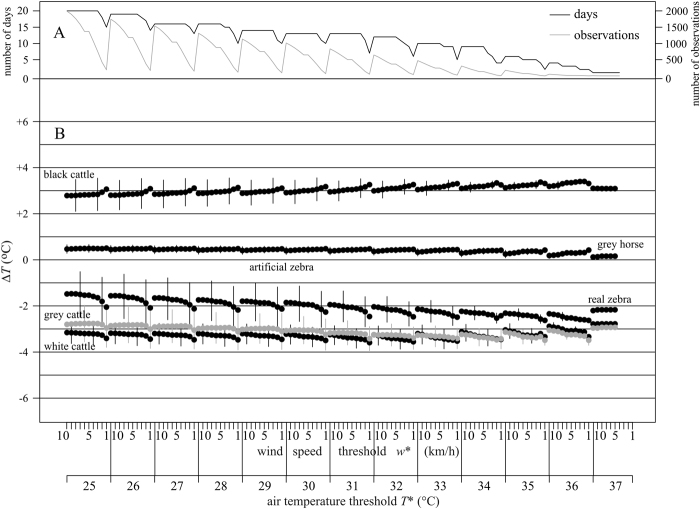
Comparison of the core temperature of barrels. (**A**) *Black curve*: Number of days when the wind speed *w* and air temperature *T*_air_ satisfy the conditions *w* < w* and *T*_air_ > T* at least once in experiment 3, which weather conditions favour the formation of cooling air eddies above zebra-striped surfaces. *Grey curve*: Number of observations when the instantaneous *w* and *T*_air_ satisfy the conditions *w* < w* and *T*_air_ > T* in experiment 3. (**B**) Difference Δ*T* = *T*_i_ – *T*_artificial zebra_ (black or grey dots) between the average core temperature *T*_i_ of barrel *i* covered with a given hide (black dots, *i*: black cattle, grey horse, real zebra, white cattle; grey dots, *i*: grey cattle) and the average core temperature *T*_artificial zebra_ of the barrel covered with the artificial zebra hide in experiment 3 versus time *t* for time delay Δ*t* = 60 min. The horizontal line at Δ*T* = 0 °C means the barrel covered with an artificial zebra hide was serving as reference. Below the upper scale of the horizontal axis shows the wind speed threshold w*, decreasing periodically from 10 to 1 km/h with 1 km/h decrement at a constant air temperature threshold T* increasing from 25 to 37 °C with 1 °C increment in the lower scale. The standard deviations, represented with vertical bars, are shown only for every 5th data points for the sake of a better visualization.

In Fig. 7A the black curve shows the number of days when the wind speed *w* and air temperature *T*_air_ simultaneously fulfilled the threshold conditions *w* < w* and *T*_air_ > T* at least once in experiment 3. The grey curve of Fig. 7A indicates the number of observations when the instantaneous *w* and *T*_air_ satisfied the conditions *w* < w* and *T*_air_ > T* in experiment 3. Both the number of days and number of observations drastically decreased at wind speeds *w* ≤ 3 km/h, thus the average temperature difference was calculated for much fewer data points than at higher wind speeds.

Figure 7B shows the difference Δ*T* = *T*_i_ – *T*_artificial zebra_ (average ± standard deviation) between the average core temperature *T*_i_ of barrel *i* covered with a given hide and the average core temperature *T*_artificial zebra_ of the barrel covered with the artificial zebra hide in experiment 3 versus time *t* for time delay Δ*t* = 60 minutes as functions of the wind speed threshold 1 km/h ≤ w* ≤ 10 km/h and the air temperature threshold 25 °C ≤ T* ≤ 37 °C. We recall that the conditions *w* < w* and *T*_air_ > T* might favour the formation of cooling convective eddies above zebra-striped surfaces. According to Fig. 7B, the average core temperature of the black barrel was always higher by Δ*T* ≈ 3–3.5 °C than that of the barrel covered with artificial zebra hide. Relative to the average core temperature of the barrel covered with artificial zebra hide, the barrels covered with white cattle and grey cattle hides were always cooler by Δ*T* ≈ 3–3.5 °C, the barrel covered with grey horse hide was warmer by Δ*T* ≈ 0–0.5 °C, and the barrel covered with a real zebra hide was cooler by Δ*T* ≈ 1.5–2.5 °C.

The paired Wilcoxon test showed highly significant (p < 0.00001) Δ*T*_core_(*t*) differences between the time-dependent core temperatures *T*_core_(*t*) of the following barrel pairs: artificial zebra versus grey horse, artificial zebra vs. grey cattle and artificial zebra vs. real zebra. However, these significant differences only mean that the barrels’ core temperatures changed fully synchronously with each other. Even for Δ*T*_core_ < 0.1 °C, if this difference is present for all observations, the result of the paired Wilcoxon test is highly significant.

All the observed differences in the barrels’ core temperatures can simply be explained by the whiteness of the barrels’ covering: the lower the average whiteness of the covering, the warmer the barrel’s core temperature. The whiteness of the barrels was the following in decreasing order (Supplementary Fig. S10A): white cattle (*wh* = 1), grey cattle (0.55), real zebra (0.53), artificial zebra (0.43), grey horse (0.25), black cattle (0.03). The average core temperature of the barrels in increasing order systematically followed the same series: white cattle < grey cattle < real zebra < artificial zebra < grey horse < black cattle (Fig. 7B).

Most remarkably, all these relative thermal characteristics of the barrels were practically independent of the time delay Δ*t* (=0, 30, 60, 90, 120 min) and weather conditions (wind speed threshold w* and air temperature threshold T*; Fig. 7, Supplementary Figs S15–S33). Various w* and T* thresholds had only marginal effects: decreasing w* resulted in only little’curls’ in the Δ*T*(w*, T*) curves with usually increasing Δ*T*, and increasing T* caused slightly higher Δ*T*. With decreasing w* and increasing T*, the number of observations *N* (when the instantaneous *w* < w* and *T*_air_ > T*) decreased rapidly in all experiments. At lower *N*, random atmospheric fluctuations had more impact on Δ*T*. As *N* decreased (parallel to the decrease of the number of days when the average *w* < w* and average *T*_air_ > T* coexisted), the standard deviation of Δ*T* also decreased. An increasing time delay Δ*t* resulted in slightly smaller Δ*T*-values. For any experiment, comparing the Δ*T*(w*, T*) curves belonging to Δ*t* = 0 and 120 min, the average Δ*T* difference was always lower than 0.5 °C.

## Discussion

In sunshine, convective air eddies are predicted to form above a striped zebra body due to upwelling and downwelling air currents above black and white stripes, respectively^14,17,20,35,36^. Such eddies can form only over nearly horizontal striped surfaces, as noted by Larison *et al*.^36^. If the zebra body is not illuminated by direct sunlight, such convective eddies cannot form because of the lack of temperature differences between adjacent black and white stripes. Convective eddies might cool a sunlit body theoretically, which would be an advantage in hot, sunny weather. In sunshine, above the horizontal topmost zones of the shell of our striped barrels such eddies might have been formed, especially in calm weather. Even more, the sunlit striped barrels in our experiments reproduced the temperature patterns of sunlit zebras (see also^37,41^). Therefore, in our experiments some cooling by stripes would be expected, that is, significantly lower core temperatures inside the striped water-filled barrels compared to the barrel covered by grey horse hide with a similar average whiteness. Our main question was whether convective air eddies above sunlit black-white striped cylindrical bodies (water-filled barrels) modelling the torso of zebras could be an effective cooling process or not, compared to monochrome grey bodies of similar average whitenesses. Importantly, we did not find any indication of such supposed cooling effect in any of our four field experiments.

Even if stripe-induced convective eddies might have formed above our striped barrels in sunshine, their cooling effect was not reflected in the core temperature, which was predominantly governed by the average whiteness of the hide covering the barrel shell, that is by the net amount of sunlight absorbed by the hide. According to our results, in sunshine, black horses or black cattle are always suffering from the strongest heating, while white ones are the least affected, and zebras and grey horses/cattle are intermediate. This conclusion is supported by the field data of Caro^4^ obtained for black buffaloes, striped zebras, light brown impalas and mottled giraffes.

In hot weather, zebras – like many other ungulates^35,42–44^ – could cool themselves by sweating (the evaporation of sweat water decreases the skin temperature), or altering blood flow (like e.g. pinnal cooling of African elephants in heat, or thermal isolation of hoofs of horses in snow). In our experiments we did not simulate cooling by sweating nor blood circulation. This can be the main reason why our sunlit zebra-striped barrels had typically higher *T*-differences (5–16 °C) between adjacent black and white stripes on the topmost horizontal zone of the barrel shell than the sunlit coats of zebras in the Budapest Zoo and Botanical Garden (2–10 °C). Similarly, Cena and Clark^37^ observed that the maximal temperature difference between adjacent sunlit black and white stripes of plains and mountain zebras was 9 °C.

Further arguments against a thermoregulatory function of zebra stripes are enumerated by Caro^4^. These are (i) his thermography data, (ii) eddies may not work on windy days or during animal movements, (iii) most mammals in hot arid environments are white or light yellow (as would be expected also from our data presented here), (iv) herbivores sympatric with zebras that live in the same microhabitats are not striped and they require thermoregulation too, (v) other equid species living in very hot climates (such as Asiatic and African wild asses) are not striped, and finally, (vi) mountain zebras have a “grid iron” pattern on their dorsal surface that is not conducive to setting up eddies in exactly the place where it is needed, namely on the flat dorsal surface. Our finding that in sunshine the core temperature of zebra-striped animal models was never significantly different from that of a homogeneous grey model with similar average whiteness provides critical experimental support to these observations showing no support for thermoregulation from black and white stripes.

The lack of leg-imitating vertical rods in our experiments (our barrels were placed at a height of 10 cm on the ground) could affect the wind speed around the barrels. Since wind velocity can be somewhat larger at the top height of a zebra body (1–1.5 m) than in the vicinity of the ground, cooling by wind can be stronger than for our barrels. Since the core temperatures *T*_core_ of our barrels were influenced by the same wind conditions simulataneously, differences in *T*_core_ among our barrels were weakly modified (if at all) by possible wind effects around them. On the other hand, if the barrels were on legs, like zebra torsos, then the slightly stronger wind received by them would play a greater influence. The fact, that they were set almost on the ground was providing an even better opportunity for convective eddies to be formed, since gound-near areas are less windy. Despite this, no cooling effect was found.

Blood circulation effectively homogenizes the body temperature of warm-blooded mammals^45^. The water body inside our barrels was not stagnant, since it is well known that the slightest horizontal temperature gradient induces thermal convection leading to water movement. Such horizontal temperature difference could build up most easily as a consequence of the East-West orientation of the barrels’ long axis, since the northern lower quarter of the barrel shell never received direct sunlight.

It should be noted that the occurrence of black stripes on the body sides and legs (possessing tilted and nearly vertical surfaces) is thermophysically disadvantageous, because the heating by sunlight absorption of black stripes would not be compensated by the cooling effect of convective air cells. In spite of this, the sides and legs of zebras are also striped. This further contradicts the hypothesis of cooling by convective air eddies above zebra stripes.

Mammals have evolved several ways to regulate body temperature, including coat colour, hair structure and physiological mechanisms^42–46^. Our field experiments indicate that zebra stripes are not part of this system.

## Conclusions

From the results of our four field experiments we conclude the followings:

- The core temperature *T*_core_ was always the highest in the black-covered barrel.
- *T*_core_ was always the lowest in the white-covered barrel.
- *T*_core_ of the barrels covered by zebra-striped and homogeneous grey hides was between that of the white- and black-covered barrels.
- Although the average *T*_core_ of the barrel covered by grey horse hide was warmer by Δ*T* < 0.5 °C and the barrel covered with real zebra hide was cooler by Δ*T* < 1.5–2.5 °C than the barrel covered with artificial zebra hide, there were no significant differences in the core temperature between the striped and grey barrels, even on the numerous (55) hot days with average *T*_air_ > 25 °C, independently of the actual wind speed and air temperature.
- Changing the air temperature threshold T* and the wind speed threshold w*, the core temperature difference between the grey and striped barrels did not change significantly, and this was true for any value of the time delay Δ*t* with which *T*_core_ followed the changes in *T*_air_ on hot days. This suggests that convective eddies did not affect the barrels’ core temperature. The non-significant small (<1.5–2.5 °C) core temperature differences between the striped and grey barrels can be simply explained by the difference in the average whitenesses of the hides determining the net absorption of sunlight.
- All these experimental findings provide evidence against the hypothesis of cooling effect of zebra stripes, because striped coats do not keep the core temperature of the body any cooler than homogeneous grey coats with a similar average whiteness.

## Materials and Methods

### Barrel experiments

Experiment 1 was conducted between 10 and 30 June 2017 in a horse farm on Göd (47°43′N, 19°09′E, northern Hungary), and was repeated between 6 and 27 July (experiment 2), 6 and 26 August (experiment 3), as well as 30 August and 19 September (experiment 4) 2017. Six metal barrels (each with a diameter = 30 cm, length = 60 cm, wall thickness = 1 mm) were filled with tap water and placed in the open air on the horse farm, arranged in two parallel rows with a horizontal spacing of 2 m (Fig. 2). From sunrise to sunset the barrels were directly exposed to sunlight (if any) and skylight, they were never in shadow. The long axis of rotation symmetry of the barrels was horizontal and parallel to the geographic East-West direction (Fig. 2). Thus, on sunny days, predominantly the cylindrical shell of the barrels was illuminated by sunlight, and around noon the most intense sunlight hit the barrel shells at maximal angles of incidence. Owing to this arrangement, sunlight absorbed by the barrel wall could warm the barrels and their water content with maximal efficiency.

The barrels were placed on wooden holders which held them at a height of 10 cm above the grassy ground (Fig. 2). At the middle of the topmost horizontal line of each barrel shell, there was a circular hole (diameter = 5 cm) through which an automatic thermometer (HOBO Pendant Temperature Data Logger, ONSET, Cape Cod, Massachusetts, USA) was suspended on the lower end of a vertical metal rod (Supplementary Fig. S34A) so that the temperature sensor was in the geometrical center of the barrel and measured the core temperature. The upper end of the metal rod was held by a circular metal cup (diameter = 7 cm) covering the hole of the barrel shell. This cup with the rod and the thermometer were stabilized by a nut of 15 gram immersed in water. The cup was painted according to the covering of the barrel (Supplementary Fig. S34B–G). The thermometers measured the water temperature in the barrels every 5 minutes continuously during the four 3-week-periods of the experiments.

We used the following coverings of the barrel shell: white cattle hide (Supplementary Fig. S34B), black cattle hide (Fig. S34C), brownish grey horse hide (Fig. S34D), Hungarian grey cattle hide (Fig. S34E), black-white-striped cattle hide called ‘artificial zebra’ (Fig. S34F), zebra (*Equus burchelli boehmi*) hide called ‘real zebra’ hereafter (Fig. S34G). The horse and cattle hides were provided by Hungarian horse and cattle keepers, while the zebra hide was obtained from a Hungarian zoological garden. The raw skins were pickled with salt and formic acid. The hides were drawn onto the barrels when wet, thus after drying they tightened onto the barrel wall as close as possible. Therefore, only a thin air layer remained between the hides and the barrel shell.

In the case of the artificial zebra hide (Supplementary Fig. S34F), 50–50% of the surface was black and white with stripe widths from 2 to 7.5 cm modelling the various patterns of real zebra hides. This covering was sewed by a furrier from stripes of black and white cattle hides. These stripes were perpendicular to the long axis of the barrel, that is vertical when the barrel was laid on the ground. This pattern modelled the mainly vertical stripes of the front (cranial) half of a zebra torso. The circular heads of the (black, white, grey, artificial zebra) barrels were painted according to the colour pattern of the shell, or covered with the same (real zebra, grey cattle) hide as the barrel shell (Supplementary Fig. S35). Since these heads were exposed only for a short period to the weak sunlight near sunrise and sunset and at other times they were mostly in shade, the impacts of barrel head coatings were negligible.

In our four field experiments the following hides covered the barrels: (1) white cattle, black cattle, grey horse, artificial zebra. (2) white, black, grey, artificial zebra, real zebra. (3, 4) white, black, grey, artificial zebra, real zebra, grey cattle.

Next to the barrels, there was a stock yard where horses were kept on the sandy ground (Fig. 2), therefore the barrel coverings sometimes became dusty. Thus, every second day the barrel surfaces were cleaned with a brush, and the positions of the barrels were randomly changed within the designated experimental area.

### Registering the meteorological variables

In order to take different weather situations into consideration, an automatic meteorological station (Conrad Electronic, equipment no: 672861, Supplementary Fig. S36A) was installed beside the barrels. Air temperature *T*_air_ (measuring range: −40 and +65 °C, accuracy ±0.1 °C), and wind speed *w* (from 0 to 160 km/h, accuracy <10%) were continuously registered during the four experiments, the manufacturer’s calibration was not changed. The sensors were placed at a height of 1 m above ground to measure temperature and wind speed in the immediate vicinity of the barrels. The receiver and data logger unit of the station were placed on the ground below the sensors in a rain-proof plastic case (Supplementary Fig. S36B).

### Thermographic measurements

Temperature patterns of sunlit zebras (*Equus burchelli boehmi*) were measured in the Budapest Zoo & Botanical Garden (Hungary) in July 2016 using a high-resolution infrared camera (VarioCAM^®^, Jenoptik Laser Optik Systeme GmbH, Jena, Germany). The corresponding heatmaps of the barrels in our field experiments were measured hourly in full sunshine under cloudless sky on 18 and 30 July and 15 August 2017 from 8:00/9:00 h (=UTC + 2 h = local summer time) to 19:00 h. To determine the spatial patterns of surface temperature distribution on zebras and barrels along straight lines, we used a self-developed software.

### Spectroscopic measurements

Spectroscopic measurements were carried out at noon under a cloudless sky in full sunshine. The measurement of the reflection spectra of the six barrels was performed within 15 minutes, during which time the illumination conditions practically did not change. The reflected light spectra *I*(λ) of the barrel coverings were measured with a spectrometer (Ocean Optics STS-VIS, Largo, USA) for wavelengths 350 nm ≤ λ ≤ 825 nm, where *I* is the radiance of surface-reflected light. For a given barrel, *I*(λ) was measured at 5 different points of the barrel surface, and these 5 spectra were averaged. The integral $INT={\int }_{350{\rm{nm}}}^{825{\rm{nm}}}I({\rm{\lambda }})d{\rm{\lambda }}$ and whiteness *wh* = *INT*/*INT*_white cattle_ (*wh* = 1: white, *wh* = 0: black) were calculated, where *INT*_white cattle_ is the spectrum integral of the white cattle hide.

### Comparison of the core temperatures of different barrels

#### Averages and standard deviations of the core temperatures of barrels

We calculated the averages (arithmetic means) and standard deviations of the core temperatures of barrels for each day of experiments 1–4 between 12:00 and 18:00 h (UTC + 2 h, experiments 1–3) and between 12:00 and 17:00 h (experiment 4). We also calculated the averages and standard deviations of the core temperatures only for the hot days when the average air temperature of the day was higher than 25 °C (i.e. when cooling air eddies above zebras were the most likely to form).

#### Statistical analysis

Applying ANOVA with Bonferroni post-hoc test (Statistica 7.0), we tested if there are differences between the core temperatures of barrels. The input data were the daily averages of the barrels’ core temperature. Statistical tests were performed (i) each day, and (ii) only for the hot days (with average *T*_air_ > 25 °C) separately for all four experiments.

### Filtering data with thresholds of the air temperature and wind speed

We assumed that the two main meteorological prerequisites of formation of convective air eddies above zebra stripes are (i) exceeding a threshold temperature T*, and (ii) low wind speeds below a threshold w*. Since we did not find any data about such thresholds in the literature, we performed statistical tests at various T* and w* values. Any temperature change on the barrel’s surface affects the core temperature of the water-filled barrels with a certain time delay Δ*t*, the thermal response time. The value of Δ*t* was estimated by computing the cross correlation integral $$CC({\rm{\delta }})=$$$${\int }_{t\,{\rm{\min }}}^{t\,{\rm{\max }}}{T}_{{\rm{air}}}({\rm{\tau }}){T}_{{\rm{core}}}({\rm{\tau }}+{\rm{\delta }})d{\rm{\tau }}$$, where *T*_air_(*t*) and *T*_core_(*t*) are the recorded time series of the air and core temperatures, δ is the time lag. *CC*(δ) was evaluated for a given barrel on the warmest days (28 June, 20 July, 10 August, 1 September 2017) of experiments 1–4 between *t*_min_ = 6:00 h and *t*_max_ = 20:00 h (UTC + 2 h) as a function of time lag δ. The maximum of *CC*(δ) at time lag δ^*^ provides an estimate for the thermal response time as Δ*t* = δ^*^. R Statistics 3.2.3 was applied for cross correlation calculations.

The hypothesis to be tested is that cooling convective eddies can build up above the zebra-striped barrels when the conditions *T*_air_ > T* and *w* < w* are satisfied, and such eddies decrease the core temperature *T*_core_ after time delay Δ*t*. First, we determined those points of time *t*_cooling_ when both conditions were satisfied, then calculated the differences Δ*T*(*t*_cooling_;T*,w*) = *T*_barrel_(*t*_cooling_ + Δ*t*) − *T*_artificial zebra_(*t*_cooling_ + Δ*t*) between the core temperatures of the barrel covered with a given hide and the artificial-zebra-hide-covered barrel as functions of thresholds 25 °C ≤ T* ≤ 37 °C and 1 km/h ≤ w* ≤ 10 km/h. The core temperature of the barrel covered with the artificial zebra hide was always the reference, because this striped barrel was used in all four experiments.

#### Statistical analysis

Using Scipy module on Python 2.7.12, for all T* and w* thresholds and Δ*t* time delays we used Wilcoxon paired test to find differences between the core temperature of the barrel covered with artificial zebra hide and the barrels with grey horse, grey cattle and real zebra hide, if the number of observations *N* (when for the instantaneous *w* and *T*_air_ the conditions *w* < w* and *T*_air_ > T* were satisfied) was higher than 20.

### Ethical approval and informed consent

Our field experiment did not need a permission or approval. We confirm that no animals were killed specifically for the purpose of this study.

### Data availability

Our paper has the following supplementary materials: Supplementary Figs S1–S36. Supplementary Tables S1–S11.

## Electronic supplementary material

Supplementary Dataset 1
