# Supplementary material for: Experimental evidence that stripes do not cool zebras
Source: Sci Rep. 2018 Jun 19;8:9351. doi: 10.1038/s41598-018-27637-1 (PMC6008466; doi:10.1038/s41598-018-27637-1)
Supplement: Supplementary file 1 — Supplementary Dataset 1 [file 41598_2018_27637_MOESM1_ESM.doc]

**Supplementary Materials**

for

**Experimental evidence that stripes do not cool zebras**

Gábor Horváth^1^,*, Ádám Pereszlényi, Dénes Száz, András Barta, Imre M. Jánosi,

Balázs Gerics and Susanne Åkesson

1: Department of Biological Physics, Eötvös University,

H-1117 Budapest, Pázmány sétány 1, Hungary

*: Corresponding Author, e-mail address: gh@arago.elte.hu

This file contains the following: Supplementary Figures S1-S36

Supplementary Tables S1-S11


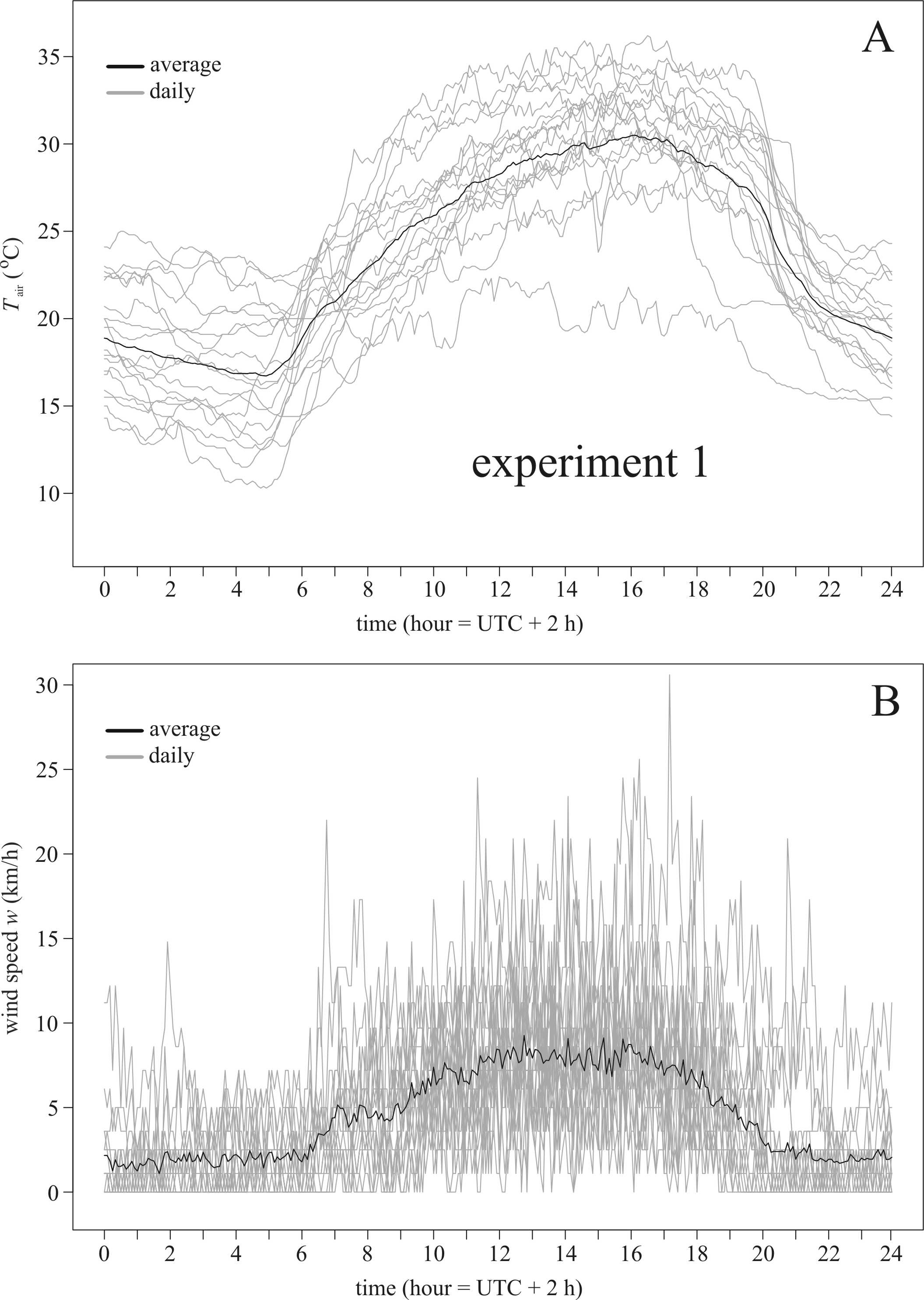


**Supplementary Figure S1**: Average (black) and daily (grey) air temperature *T*_air_ (^o^C) (A) and wind speed *w* (km/h) (B) versus time (hour = UTC + 2 h) measured during field experiment 1 between 10 and 30 June 2017.


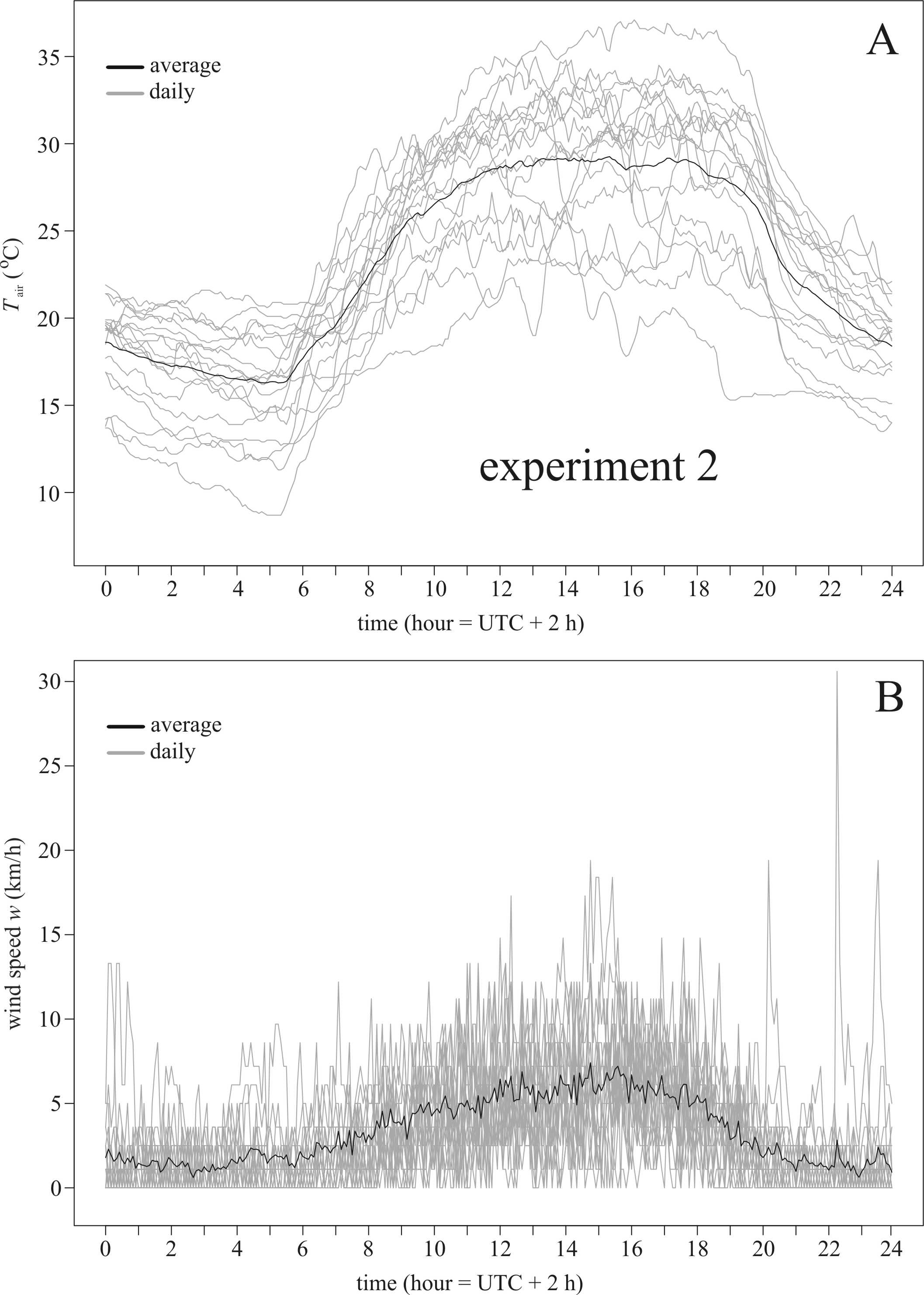


**Supplementary Figure S2**: As Supplementary Fig. S1 for field experiment 2 between 6 and 27 July 2017.


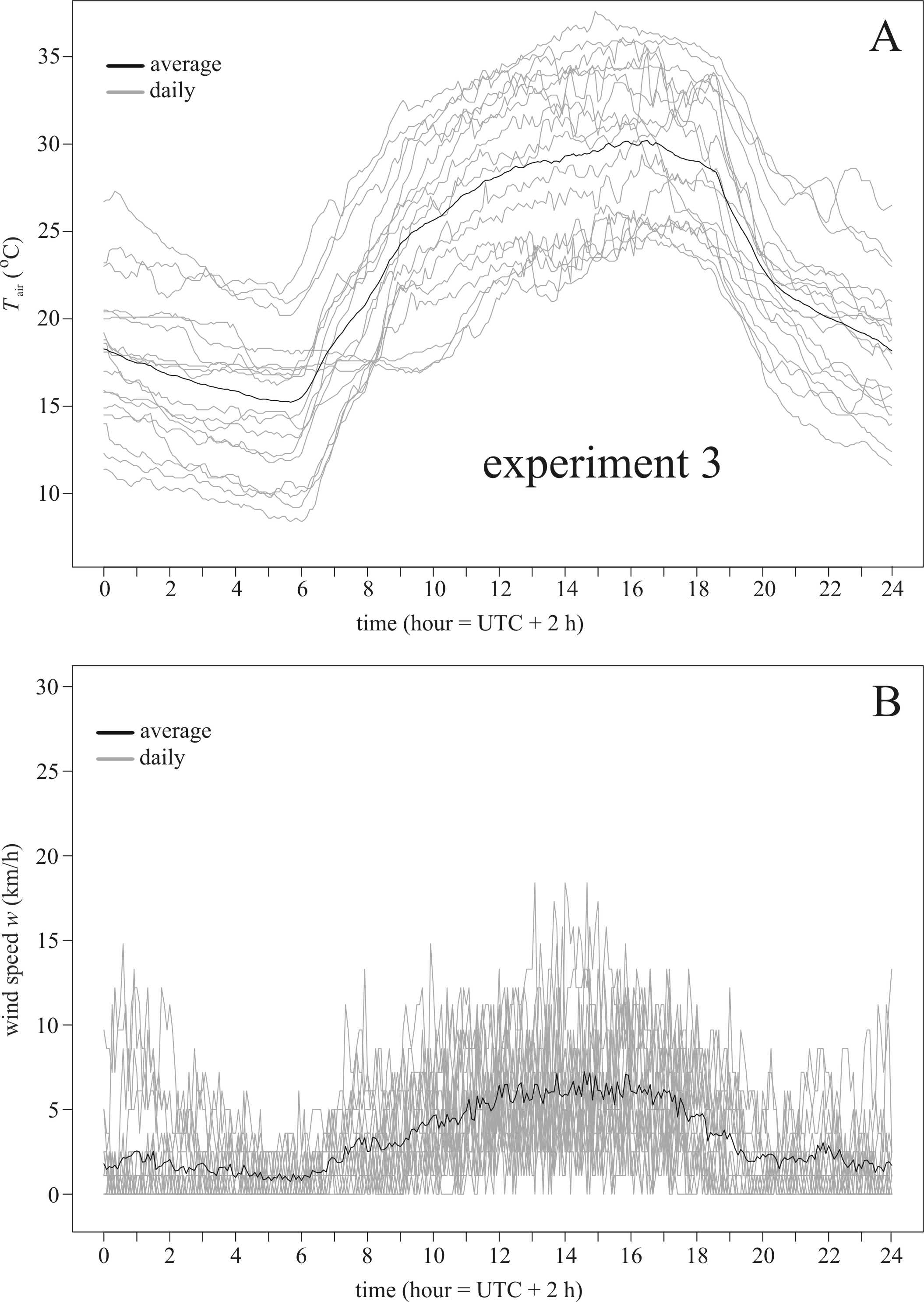


**Supplementary Figure S3**: As Supplementary Fig. S1 for field experiment 3 between 5 and 26 August 2017.


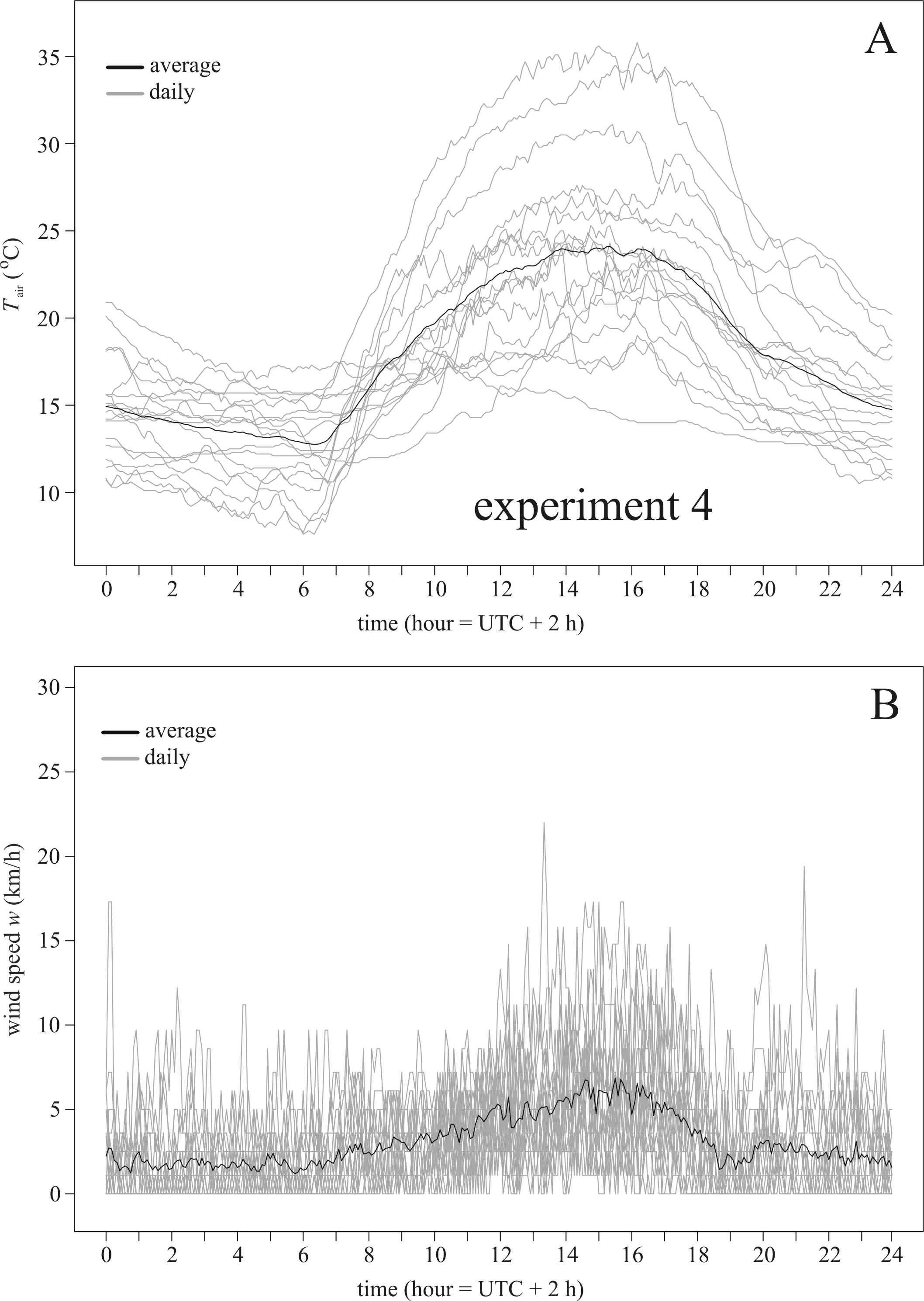


**Supplementary Figure S4**: As Supplementary Fig. S1 for field experiment 4 between 30 August and 19 September 2017.


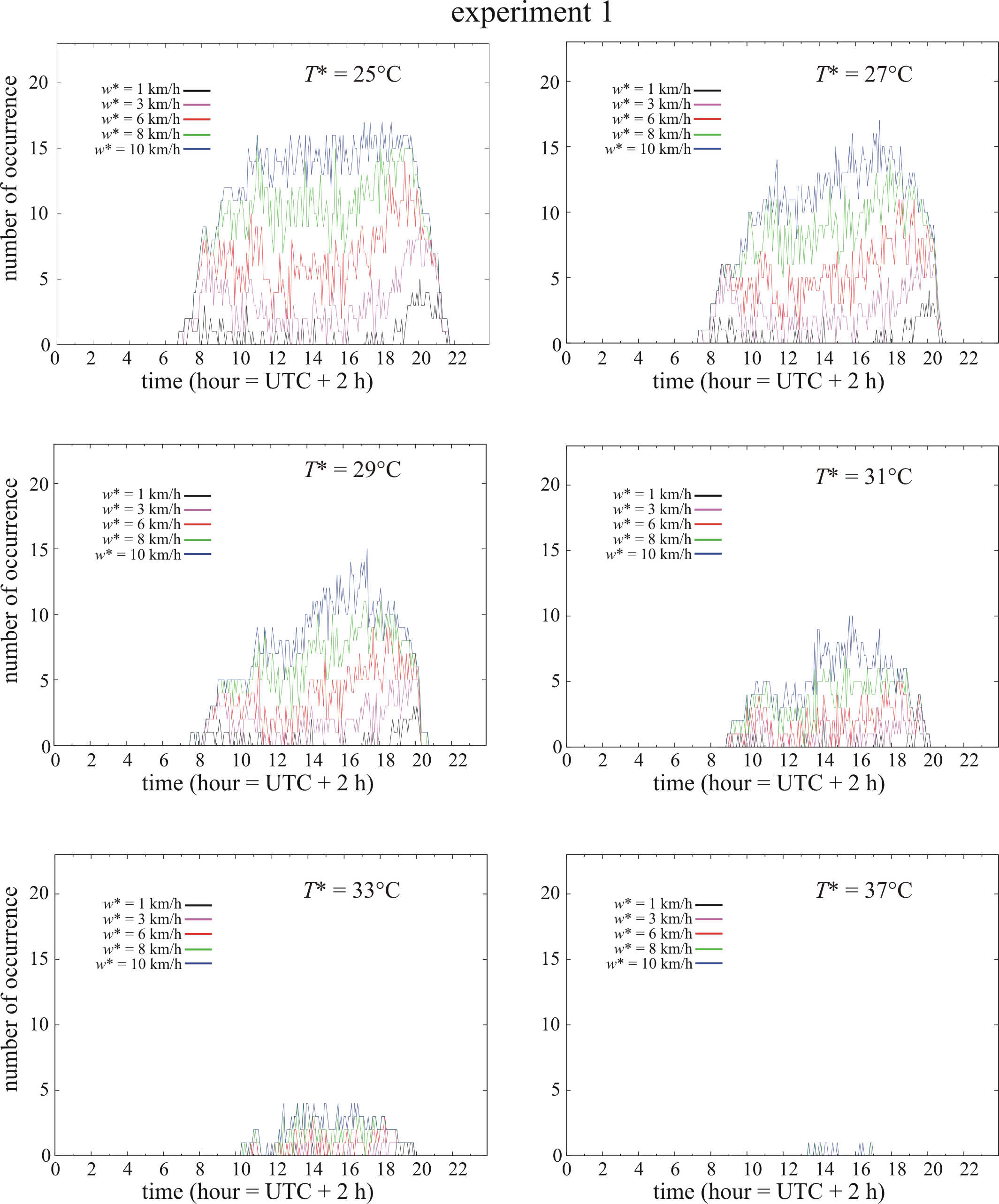


**Supplementary Figure S5**: Number of occurrences when the following two weather conditions coexisted at different points of time (UTC + 2 h) of a day during field experiment 1 in June 2017: (1) wind speed *w* < *w** (km/h) marked with different coloured lines on the graph, (2) air temperature *T*_air_ > *T** (°C).


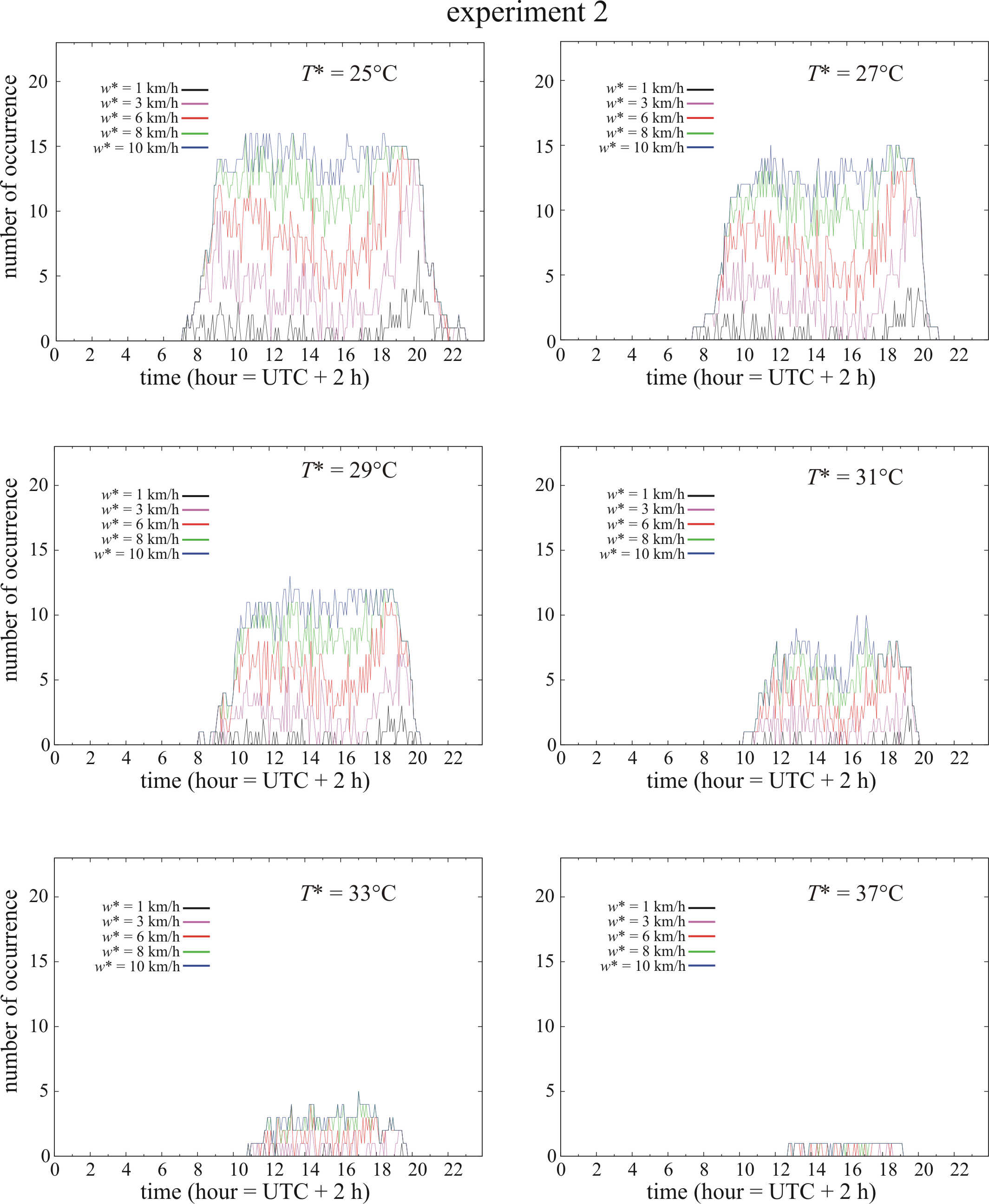


**Supplementary Figure S6**: As Supplementary Fig. S5 for experiment 2 in July 2017.


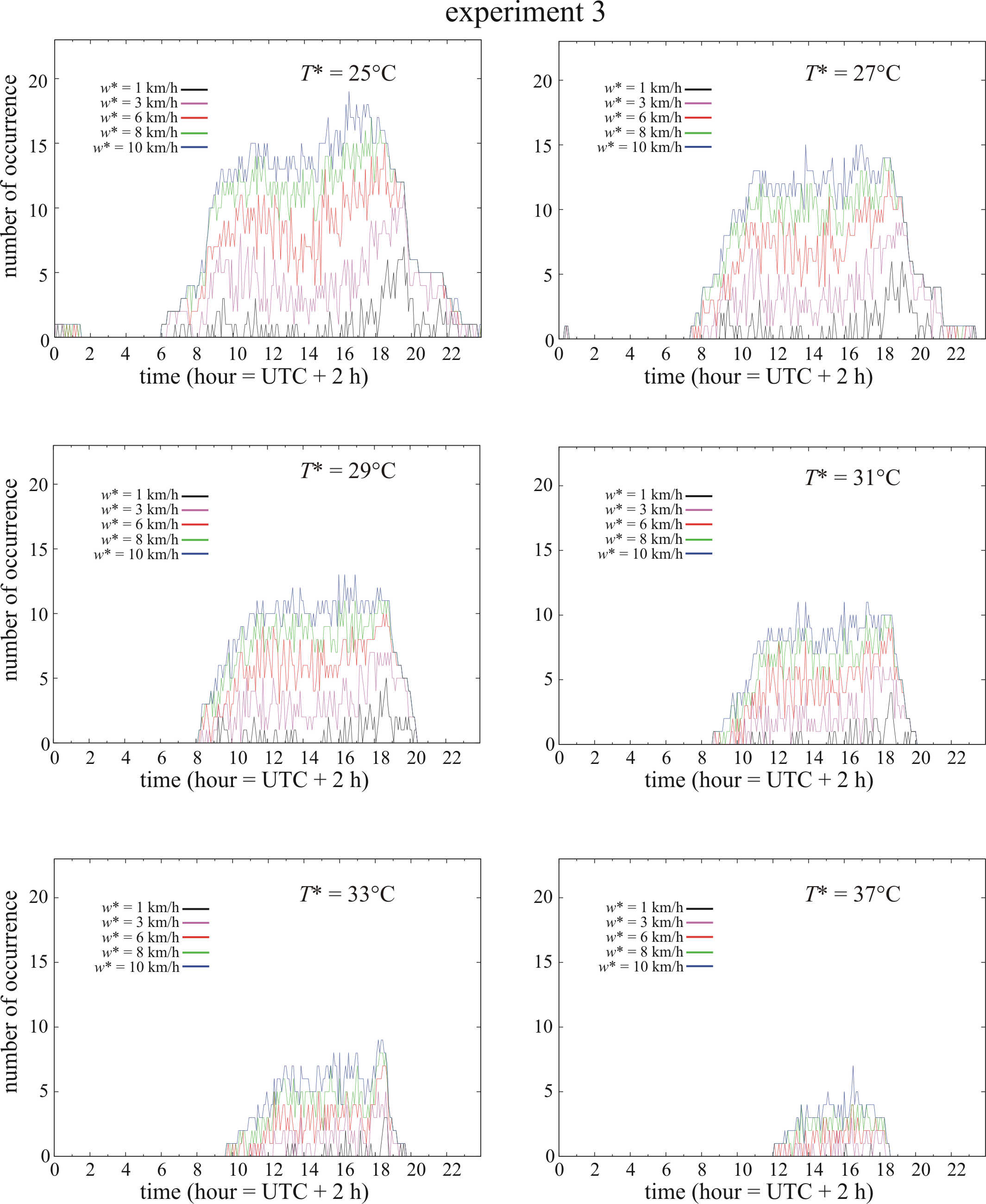


**Supplementary Figure S7**: As Supplementary Fig. S5 for experiment 3 in August 2017.


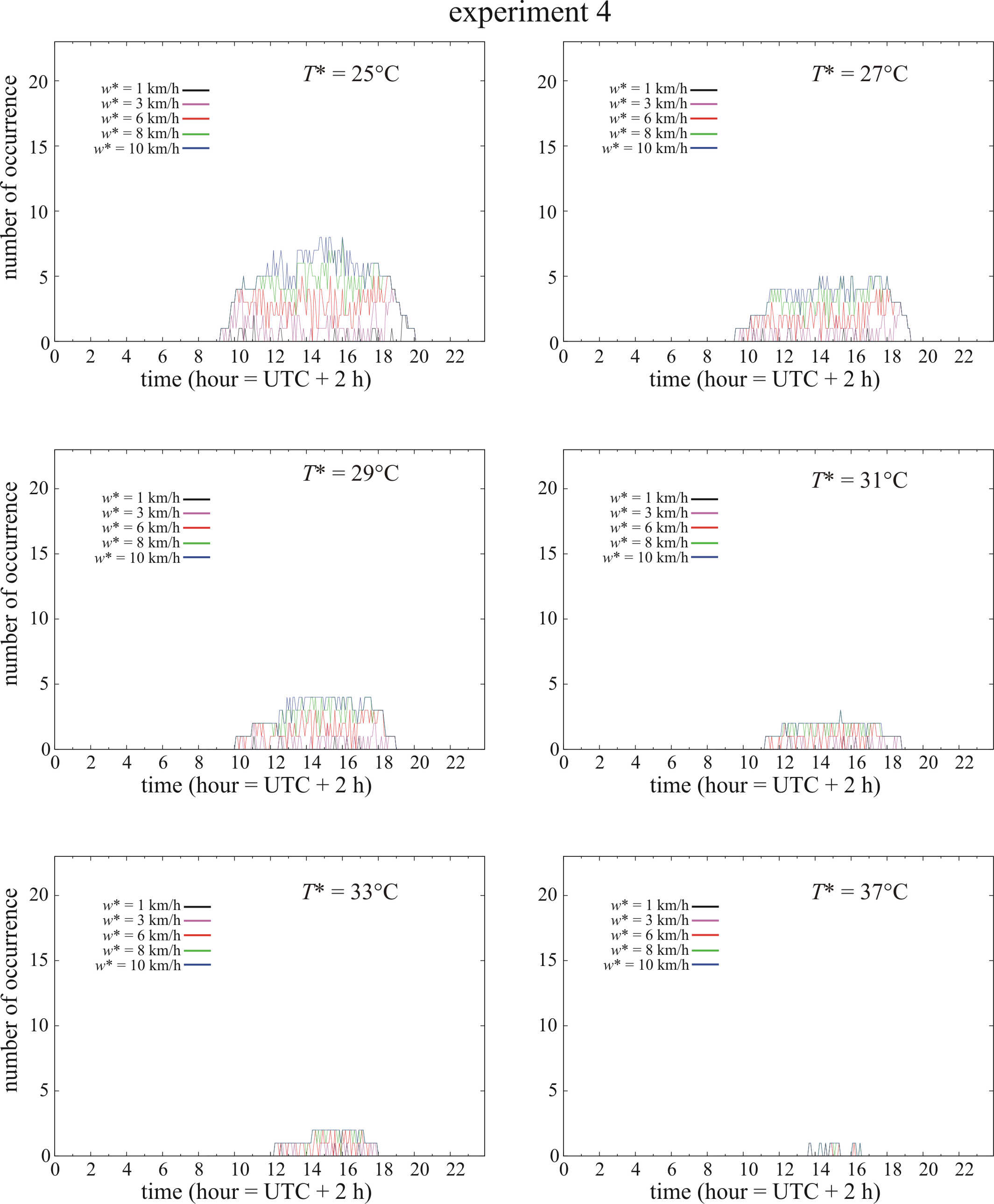


**Supplementary Figure S8**: As Supplementary Fig. S5 for experiment 4 in September 2017.


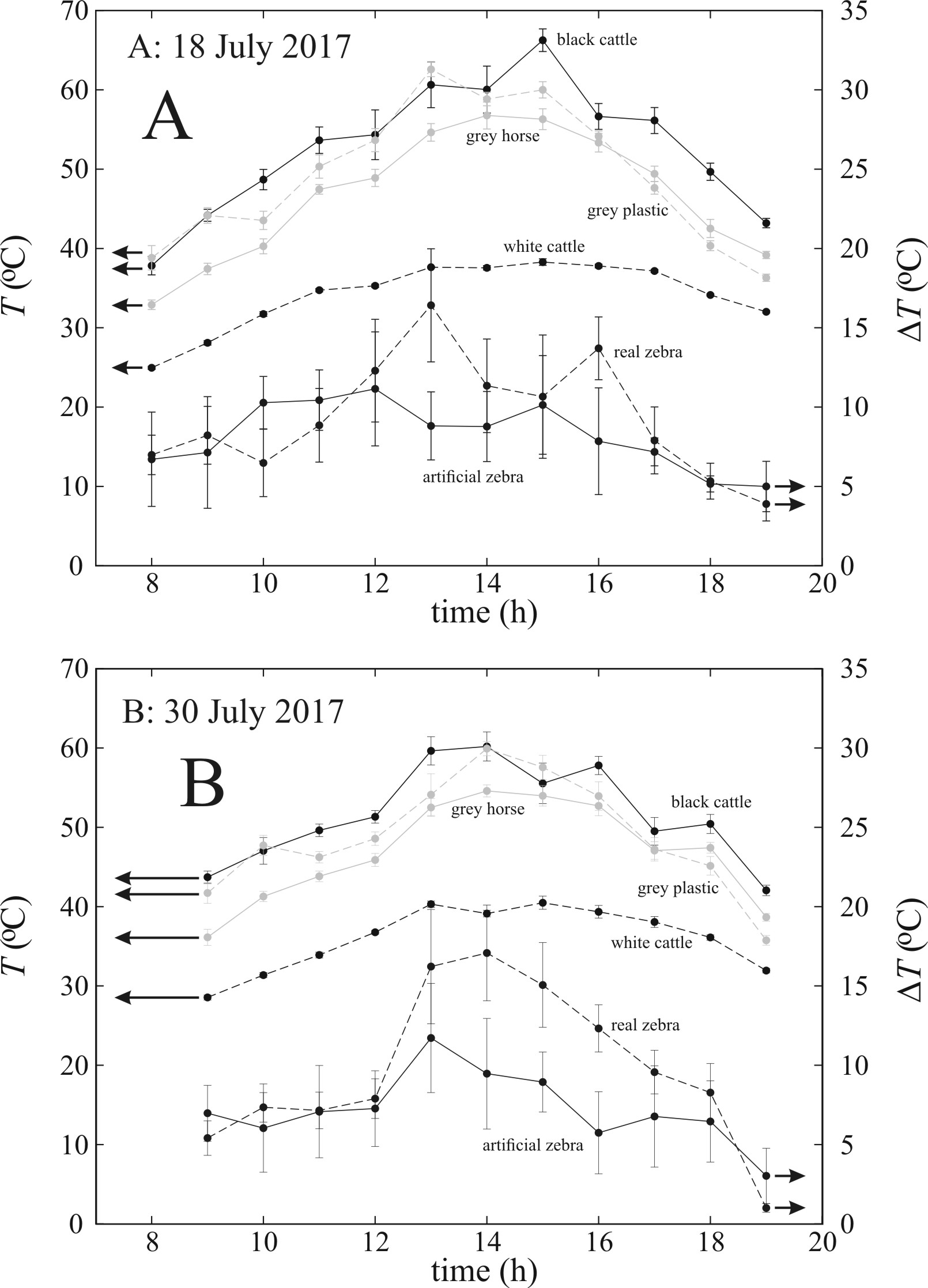


**Supplementary Figure S9**: Average surface temperature *T* (^o^C, dots) ± standard deviation σ_T_ (^o^C, vertical bars) of water-filled barrels covered by homogeneous hides, and average surface temperature difference Δ*T* (^o^C, dots) between adjacent black and white stripes ± standard deviation σ_ΔT_ (^o^C, vertical bars) of water-filled barrels covered by zebra-striped hides as a function of time (hour = UTC + 2 h). The thermograms were measured on 18 (A) and 30 (B) July 2017 with a thermocamera, the temperature data of which were gathered along a straight line on the sunlit top of the barrels (numerical data are in Supplementary Tables S1-S3). Horizontal arrows show which *T* or Δ*T* vertical scale belongs to the corresponding curves.


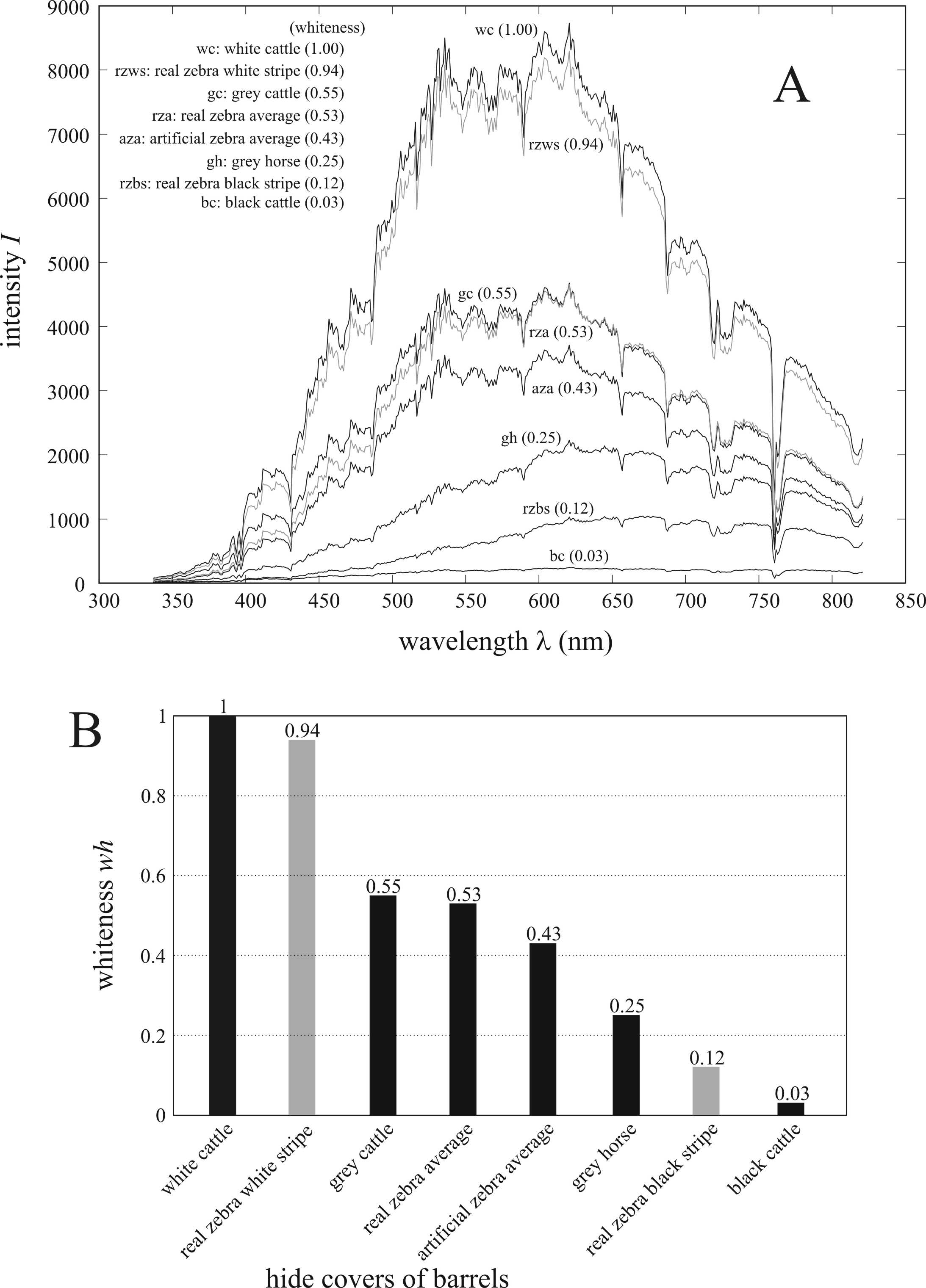


**Supplementary Figure S10**: (A) Reflected light spectra *I*(λ) and whiteness *wh* of the different hides used in our experiments. The average spectra are presented for real and artificial zebra hides: the spectra of the white and black stripes were averaged. The spectrum of the white and black stripes of the artificial zebra hide is the same as that of the white and black cattle hide, respectively, because the artificial zebra hide was composed of white and black cattle hide stripes. After the name of the hide covers the whiteness-value is given in brackets. (B) Whiteness *wh* of the different hides used in our experiments. The columns of the white and black stripes of the real zebra hide are grey, because the average of the real zebra hide is the only important value in the comparison with other covers.


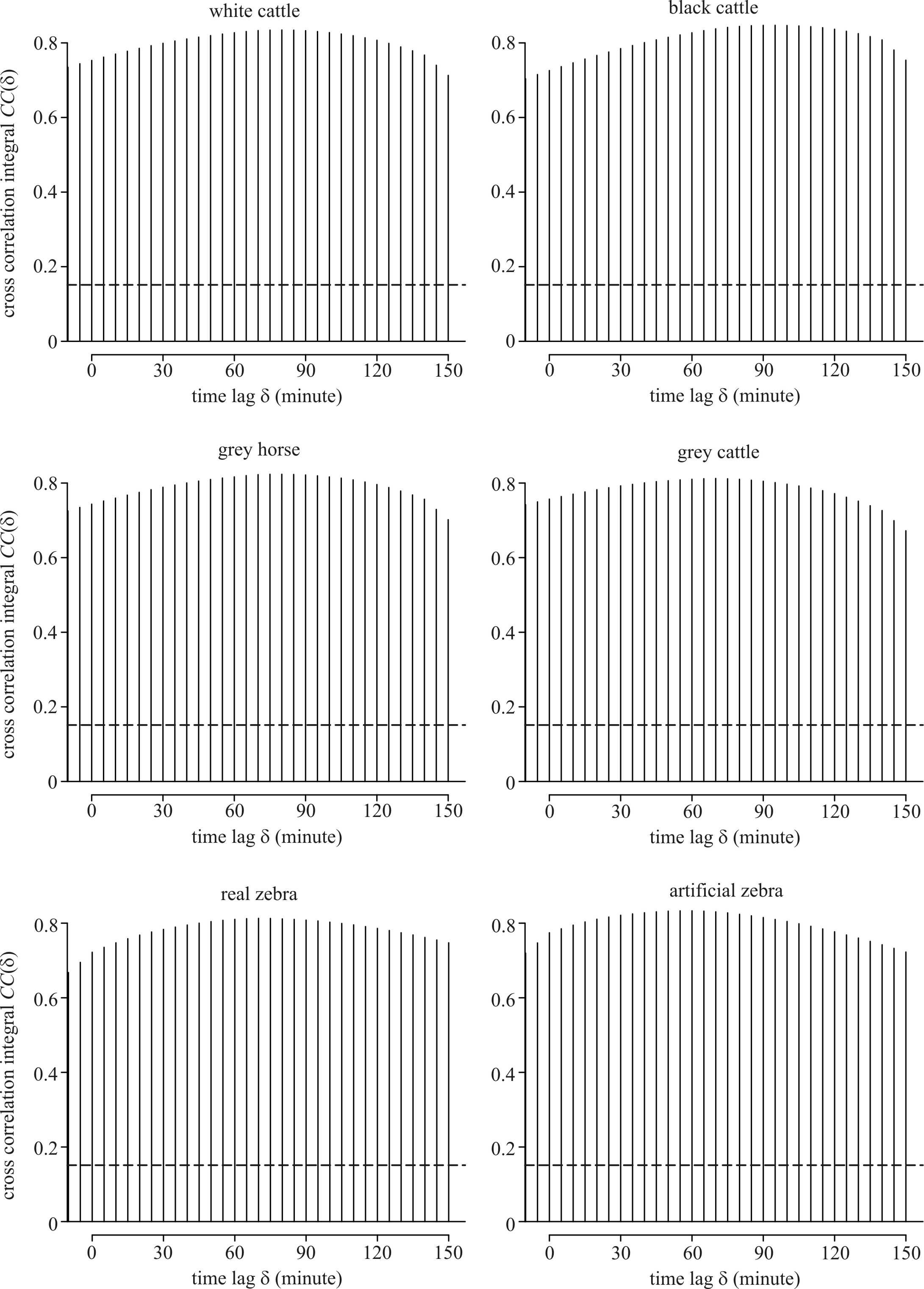


**Supplementary Figure S11**: Cross correlation integral $CC(\text{δ})=\int_{t\min}^{t\max} T_{\text{air}}(\text{τ})T_{\text{core}}(\text{τ}+\text{δ})\text{dτ}$ of the air temperature *T*_air_(*t*) and the barrel’s core temperature *T*_core_(*t*) for the warmest day 10 August 2017 of experiment 3 as a function of the time lag δ ranging from 0 to 150 minutes for the six barrels used, where *t*_min_ = 6:00 h and *t*_max_ = 20:00 h (UTC + 2 h). The maximum of *CC*(δ) at time lag δ^*^ provides an estimate for the thermal response time as Δ*t* = δ^*^ with which *T*_core_(*t*) follows the changes in *T*_air_(*t*). The horizontal dashed line shows the 5 % significance level: *T*_air_(*t*) and *T*_core_(*t*) correlate significantly at time lags δ where *CC*(δ) is higher than the significance level.


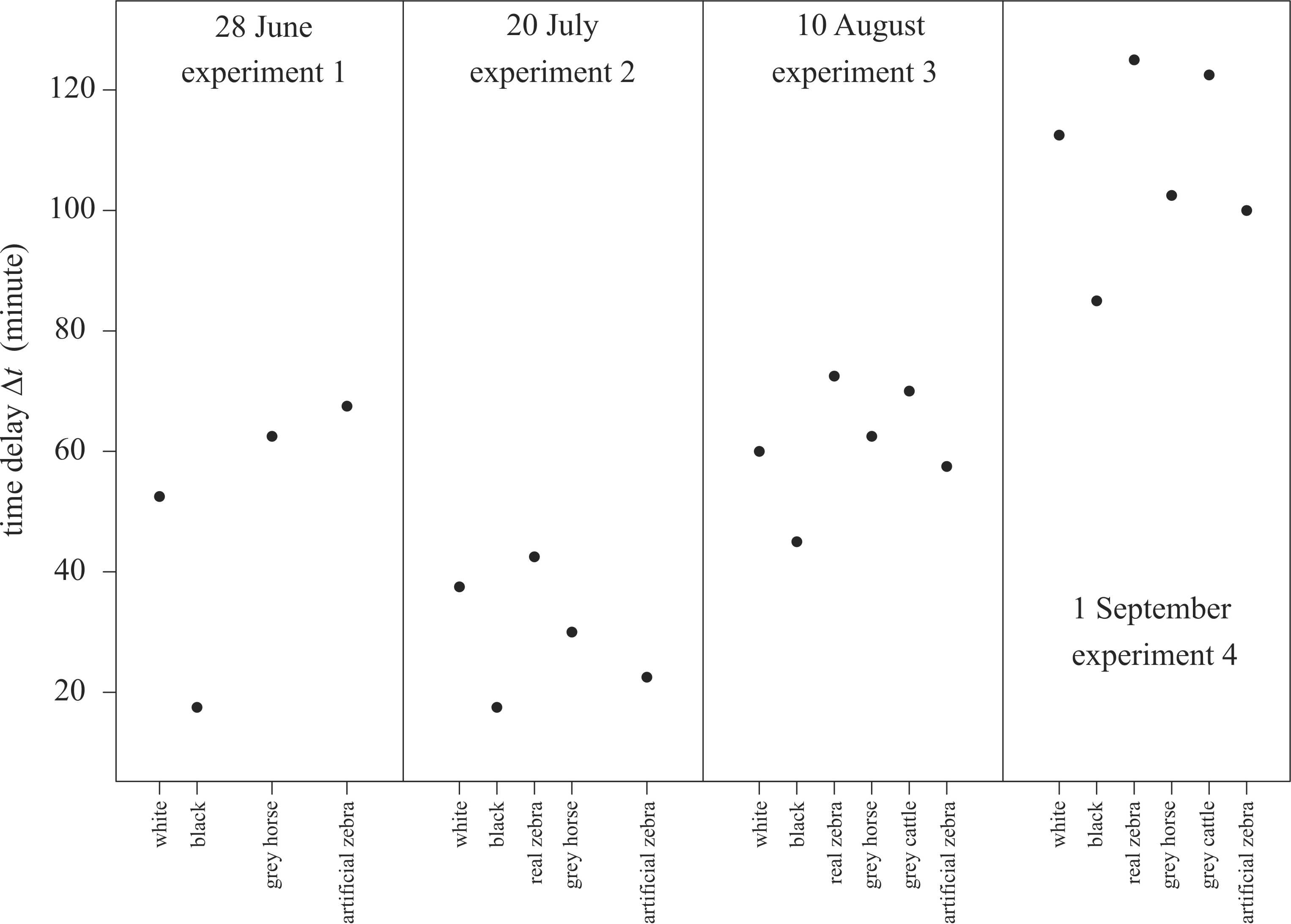


**Supplementary Figure S12**: Time delay Δ*t* (minute) with which the barrel’s core temperature *T*_core_(*t*) follows the changes in the air temperature *T*_air_(*t*) calculated for the warmest day (28 June, 20 July, 10 August, 1 September 2017) of each experiment (1-4) and for each barrel.


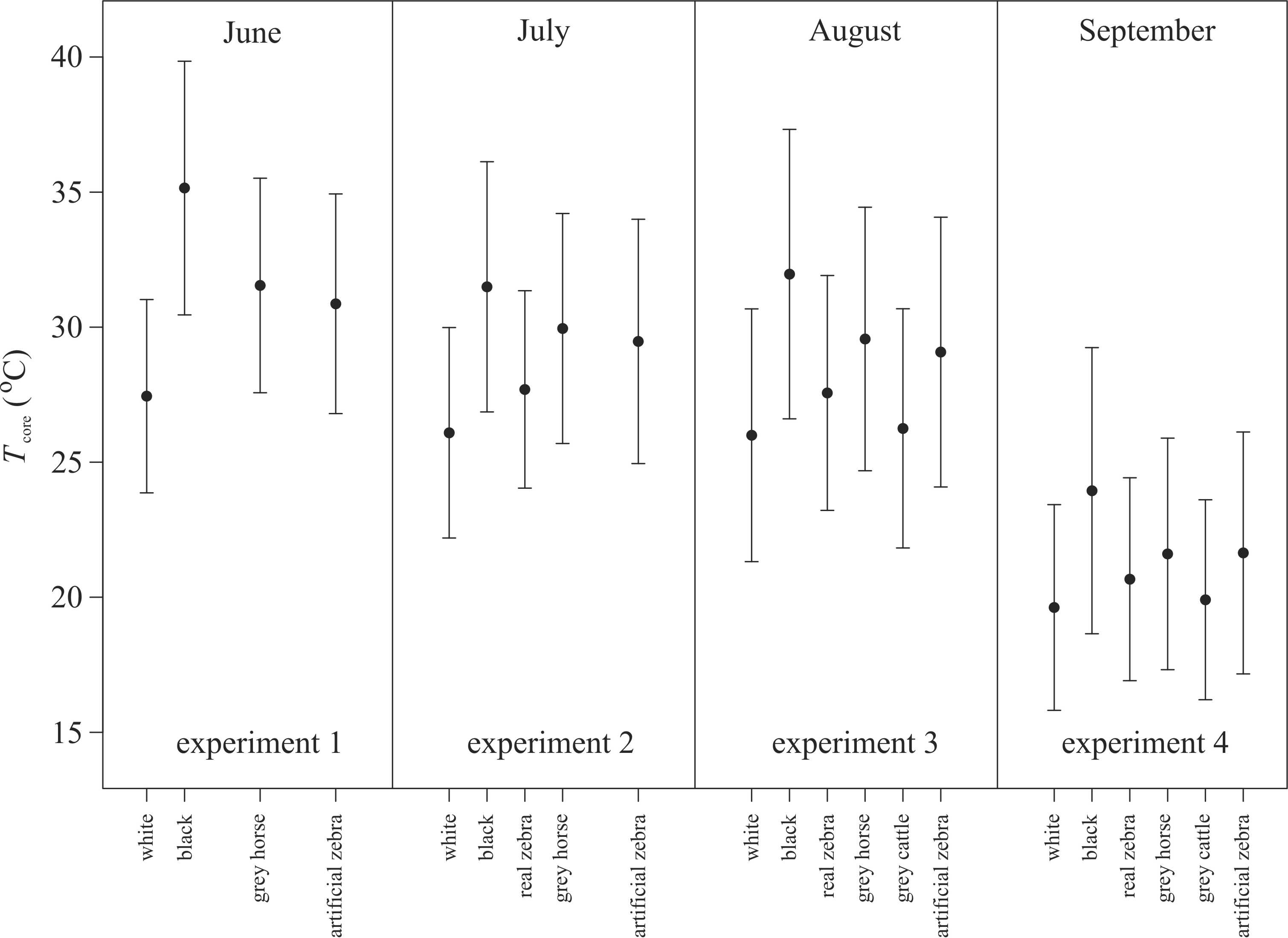


**Supplementary Figure S13**: Average (dots) ± standard deviation (vertical bars) of the core temperature *T*_core_ (^o^C) of different barrels calculated for all experimental days between 12:00 and 18:00 h (UTC + 2 h) in June (experiment 1), July (experiment 2) and August (experiment 3), and between 12:00 and 17:00 h in September (experiment 4).


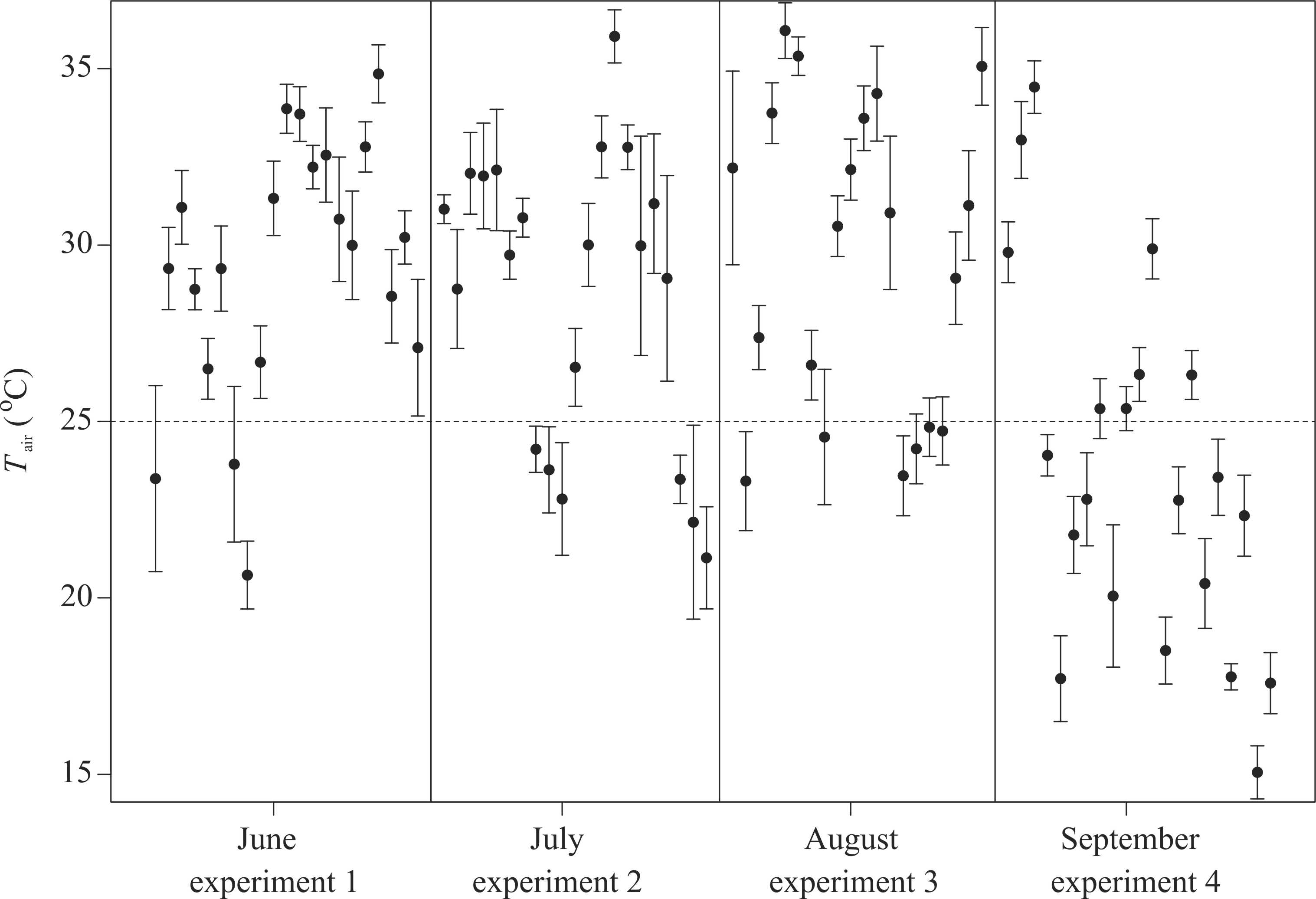


**Supplementary Figure S14**: Average (dots) ± standard deviation (vertical bars) of the air temperature *T*_air_ (^o^C) between 12:00 and 18:00 h (UTC + 2 h) on experimental days in June, July and August and between 12:00 and 17:00 h in September. Days with *T*_air_ > 25 °C (horizontal dashed line) are used for calculation of the averages and standard deviations in Fig. 2.


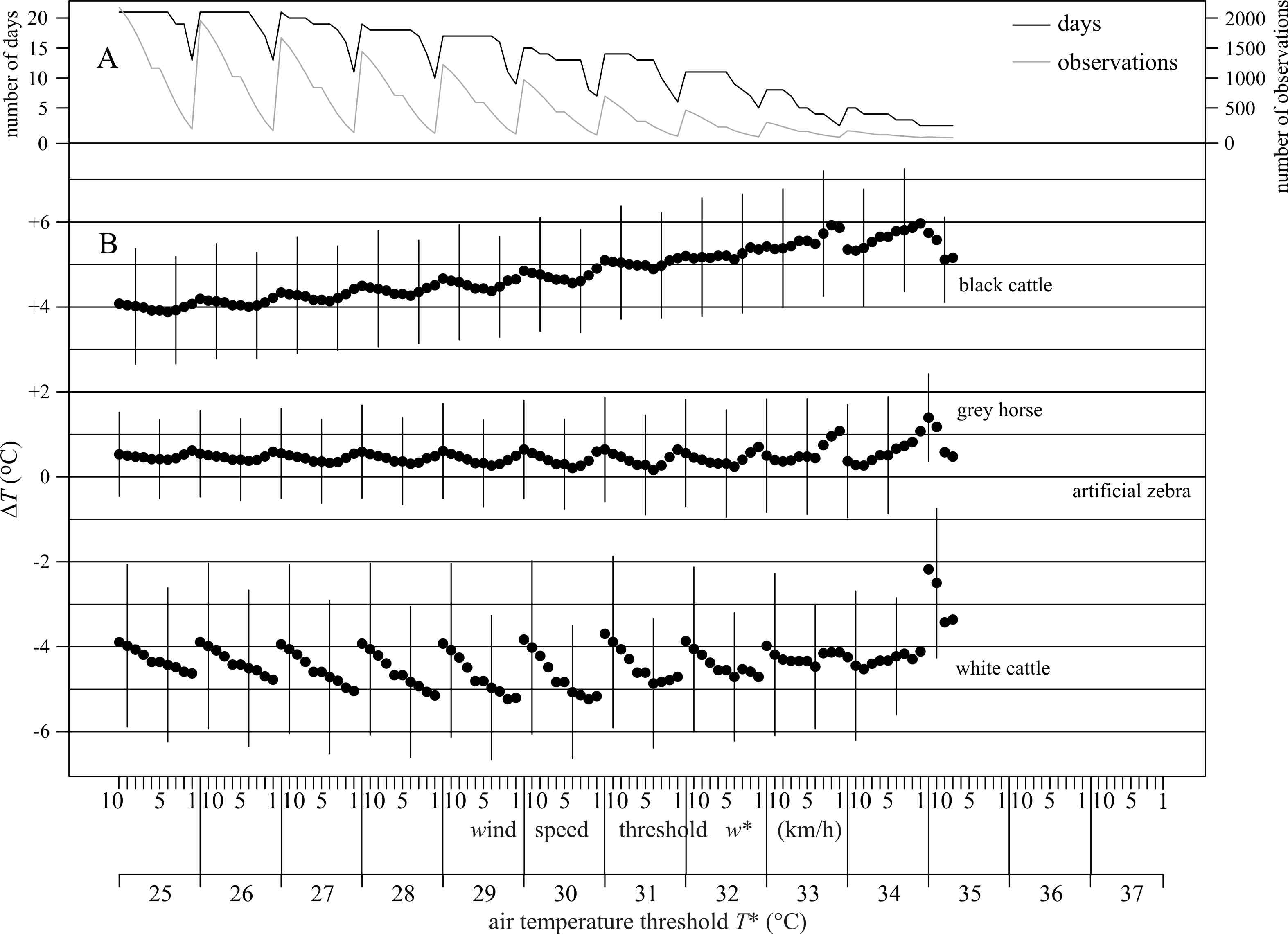


**Supplementary Figure S15**: As Fig. 7 for experiment 1 and time delay Δ*t* = 0 minute.


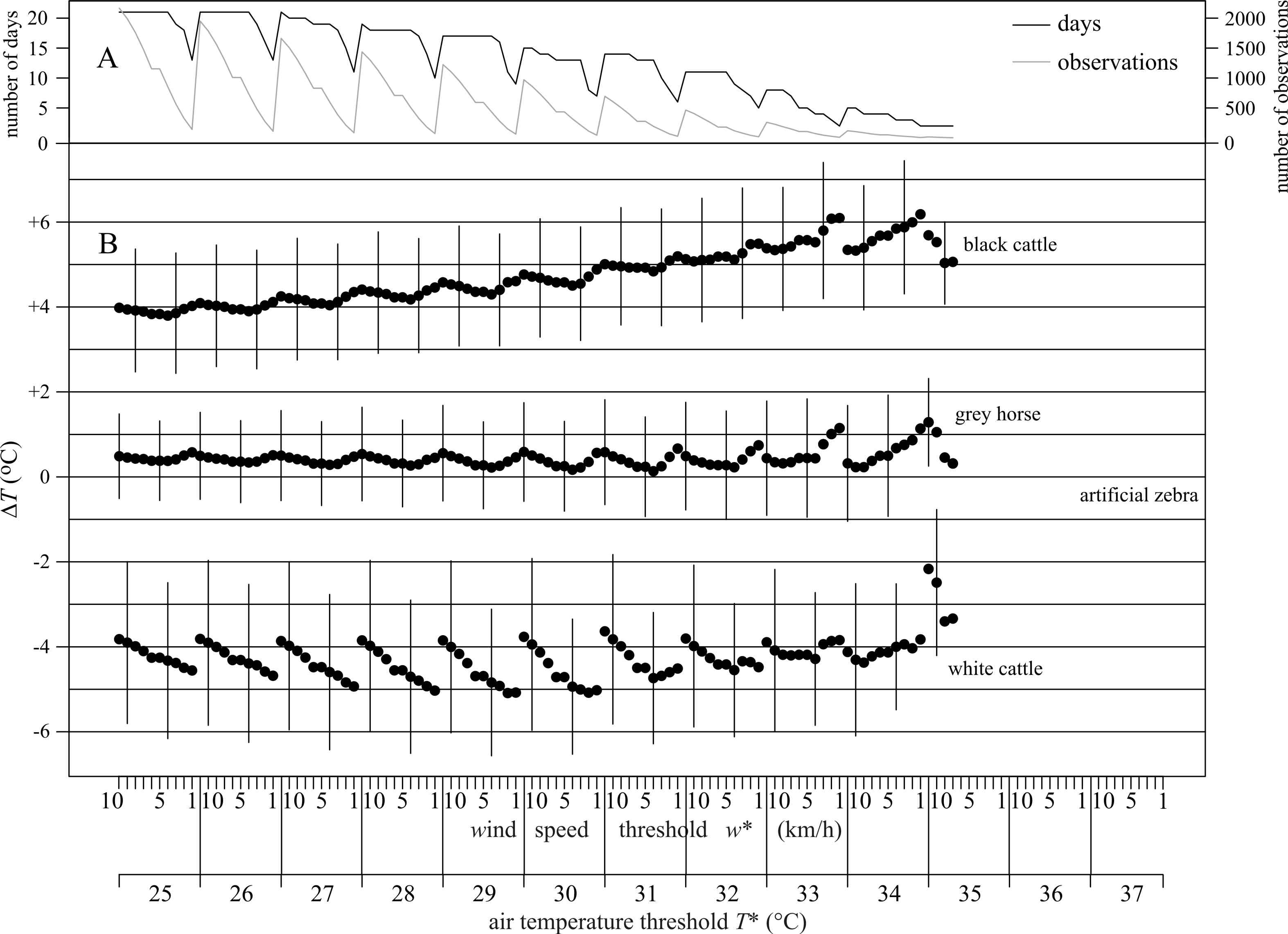


**Supplementary Figure S16**: As Fig. 7 for experiment 1 and time delay Δ*t* = 30 minutes.


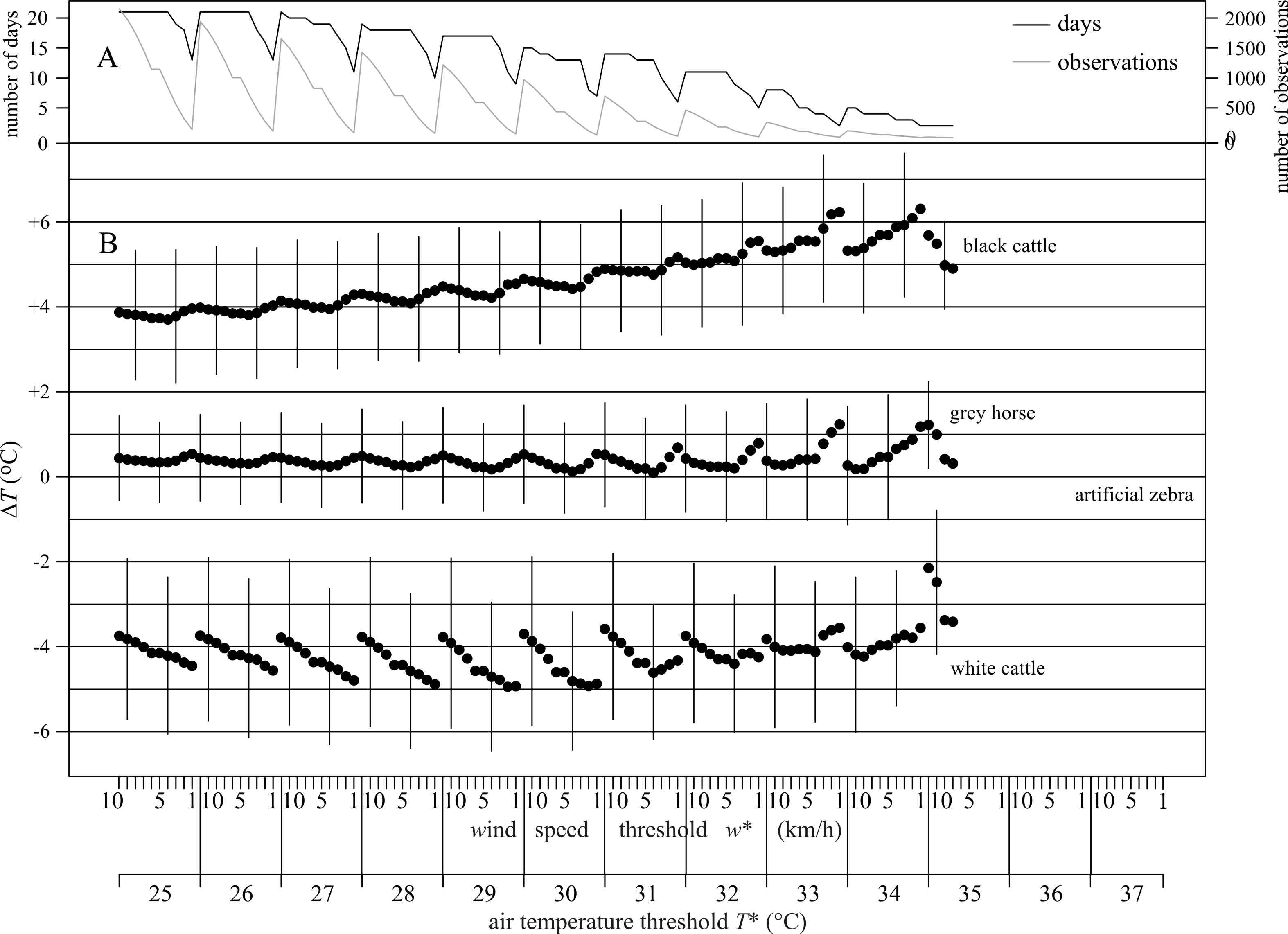


**Supplementary Figure S17**: As Fig. 7 for experiment 1 and time delay Δ*t* = 60 minutes.


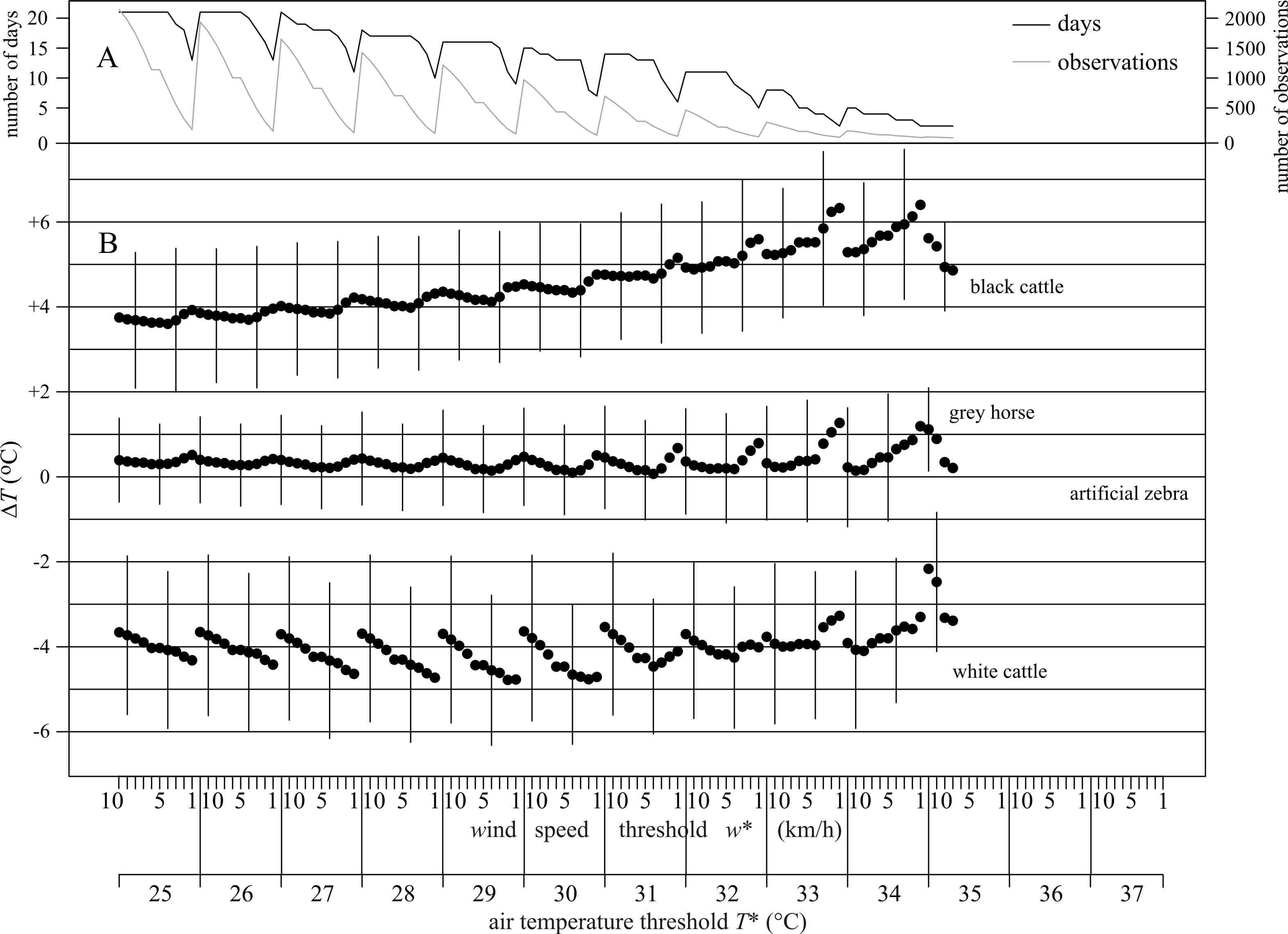


**Supplementary Figure S18**: As Fig. 7 for experiment 1 and time delay Δ*t* = 90 minutes.


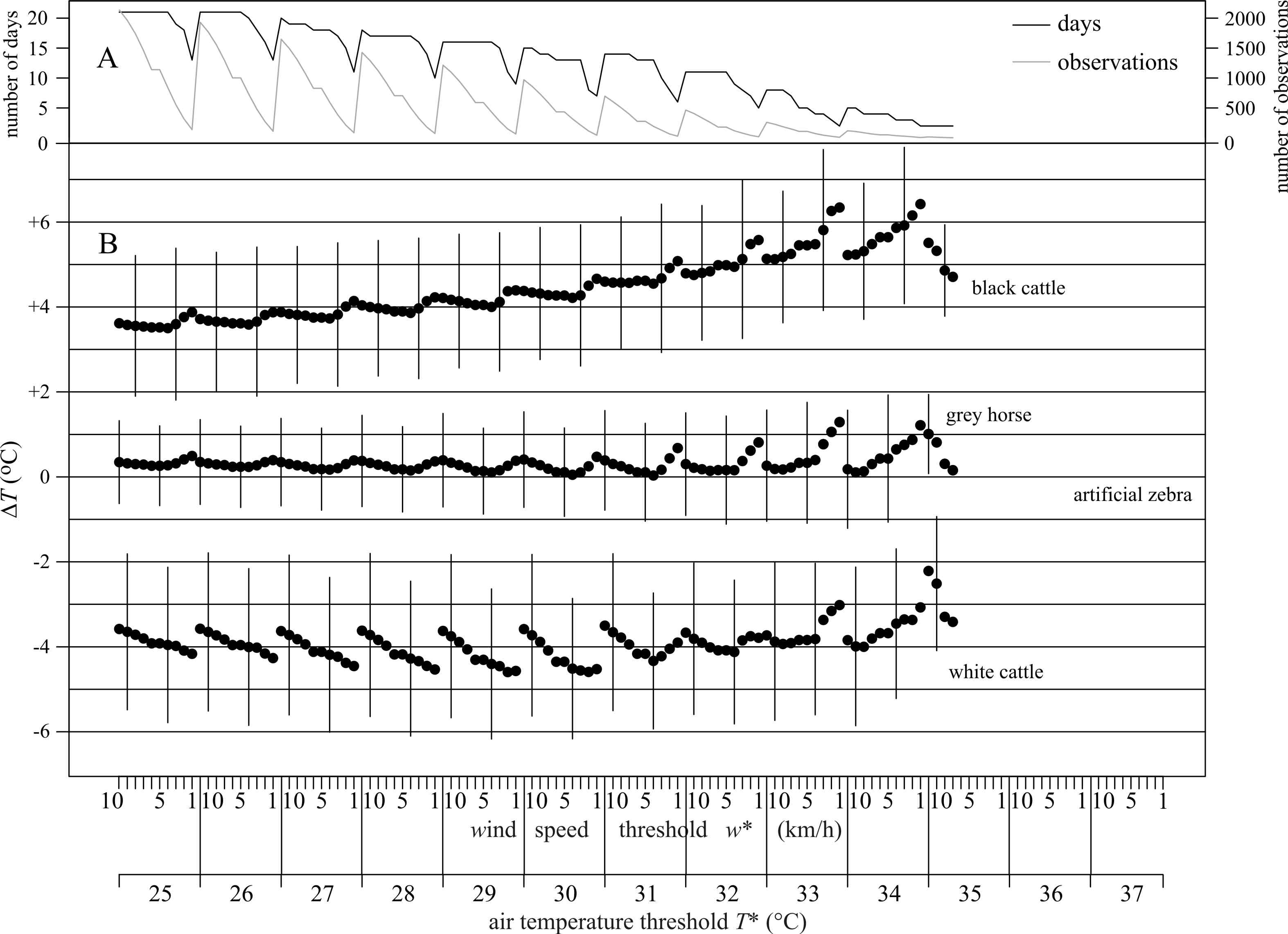


**Supplementary Figure S19**: As Fig. 7 for experiment 1 and time delay Δ*t* = 120 minutes.


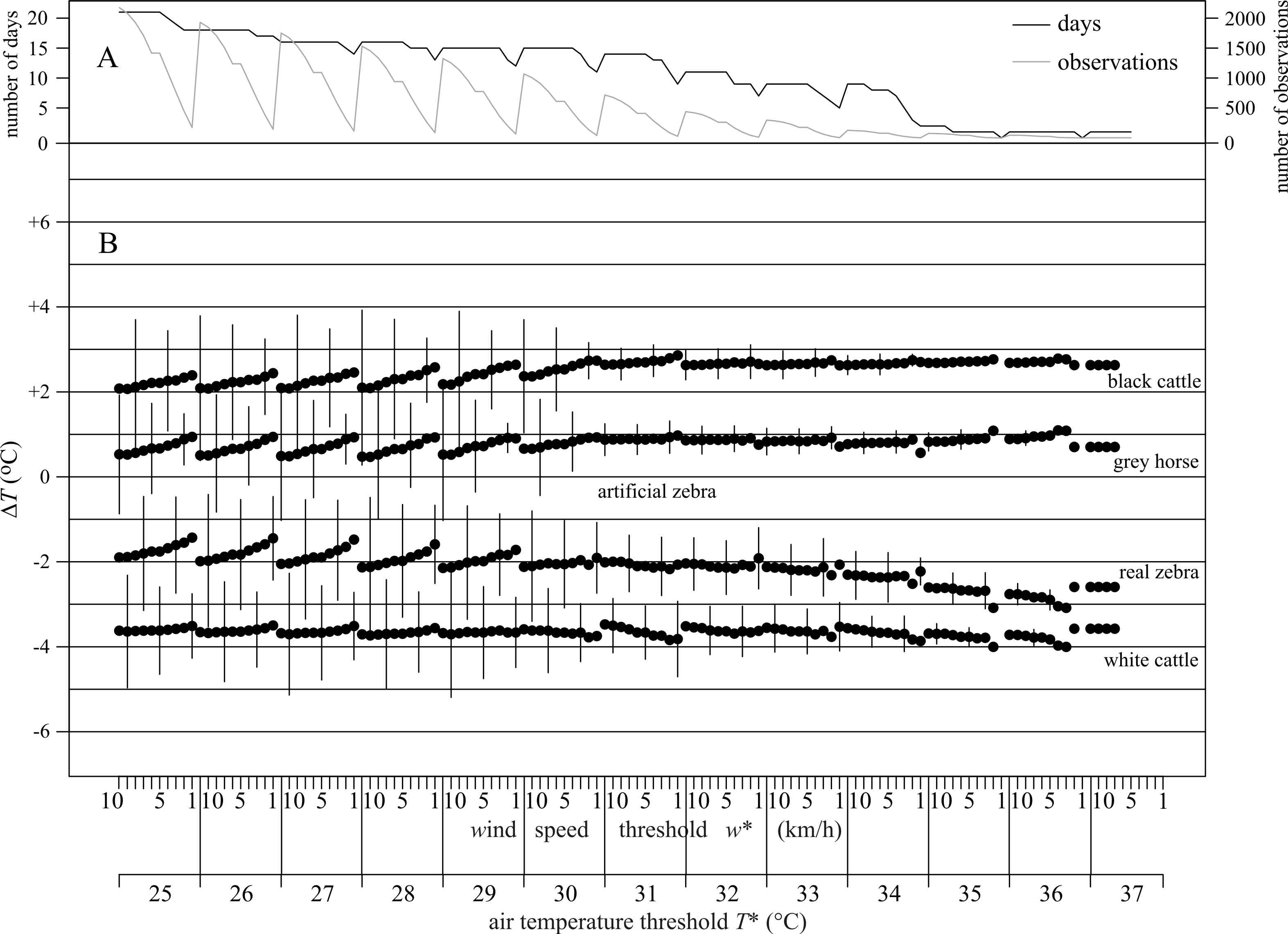


**Supplementary Figure S20**: As Fig. 7 for experiment 2 and time delay Δ*t* = 0 minute.


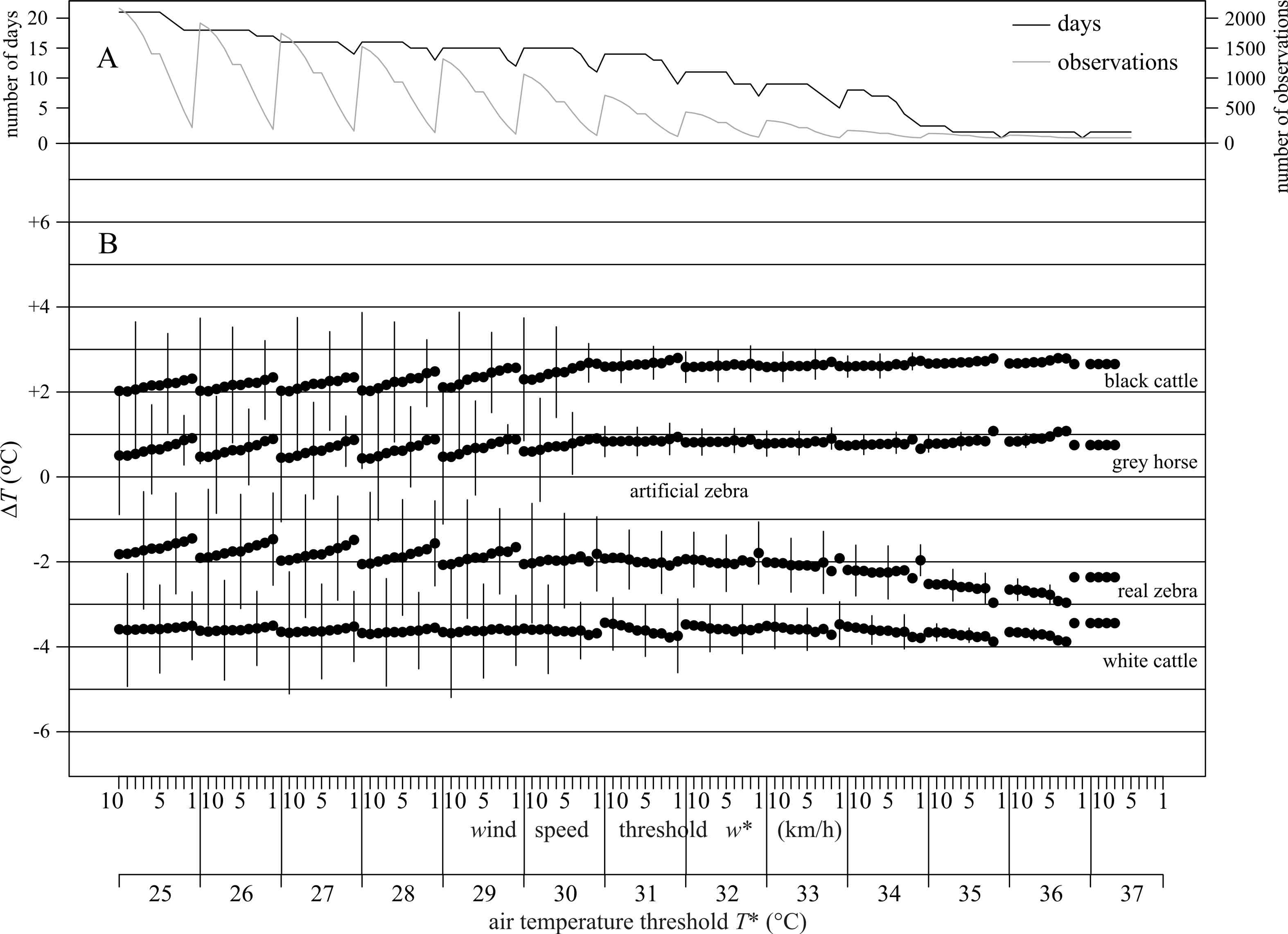


**Supplementary Figure S21**: As Fig. 7 for experiment 2 and time delay Δ*t* = 30 minutes.


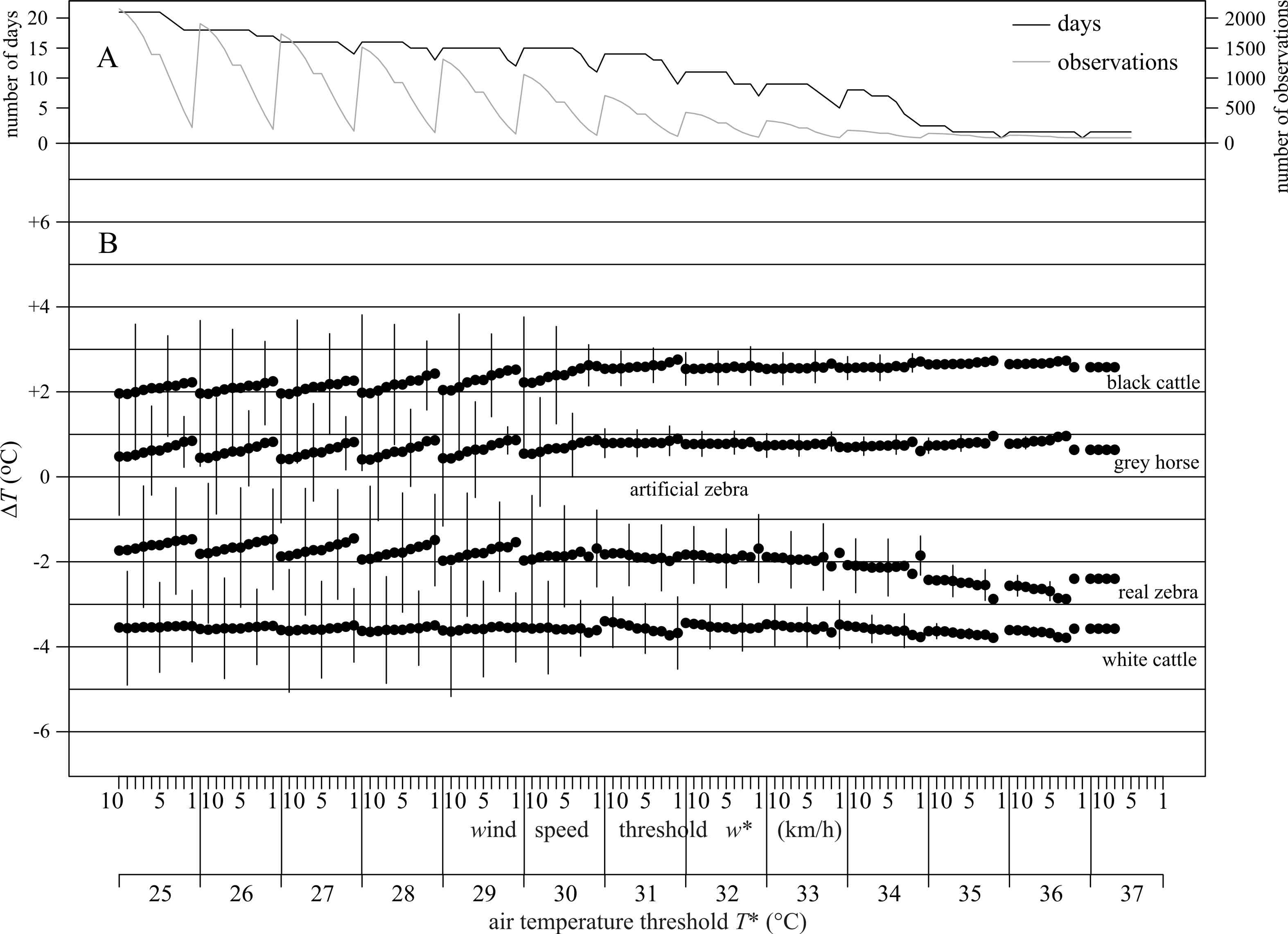


**Supplementary Figure S22**: As Fig. 7 for experiment 2 and time delay Δ*t* = 60 minutes.


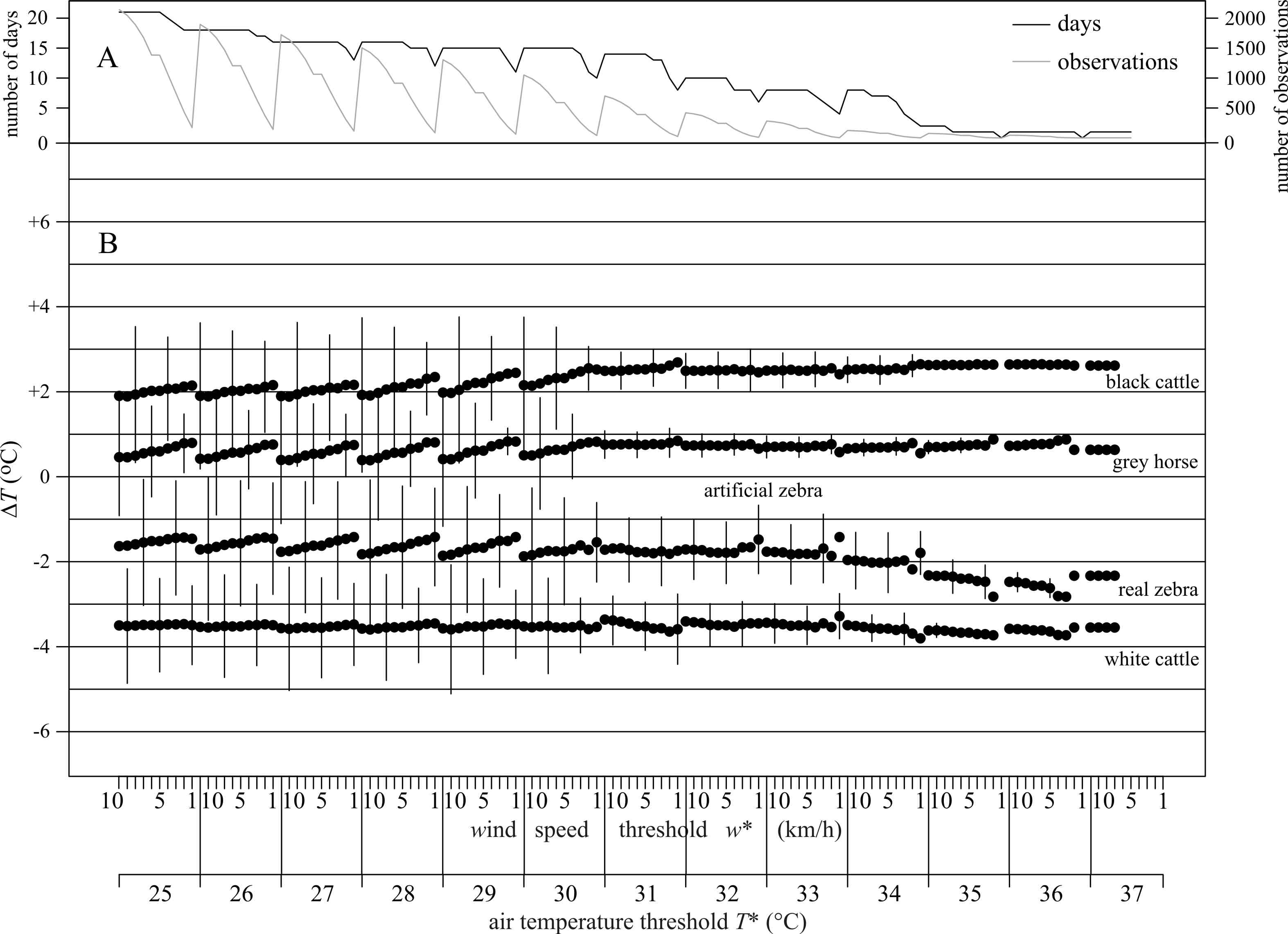


**Supplementary Figure S23**: As Fig. 7 for experiment 2 and time delay Δ*t* = 90 minutes.


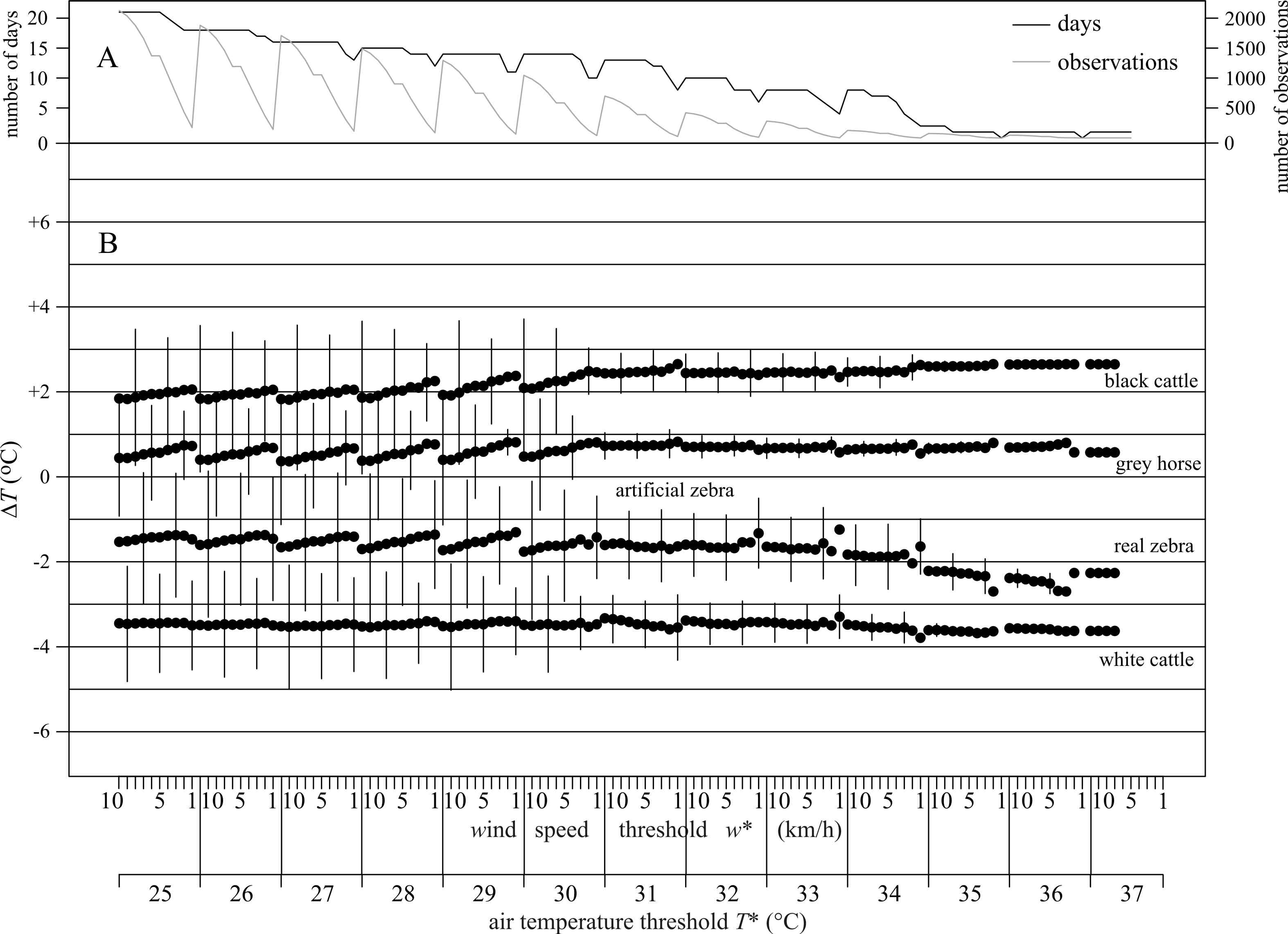


**Supplementary Figure S24**: As Fig. 7 for experiment 2 and time delay Δ*t* = 120 minutes.


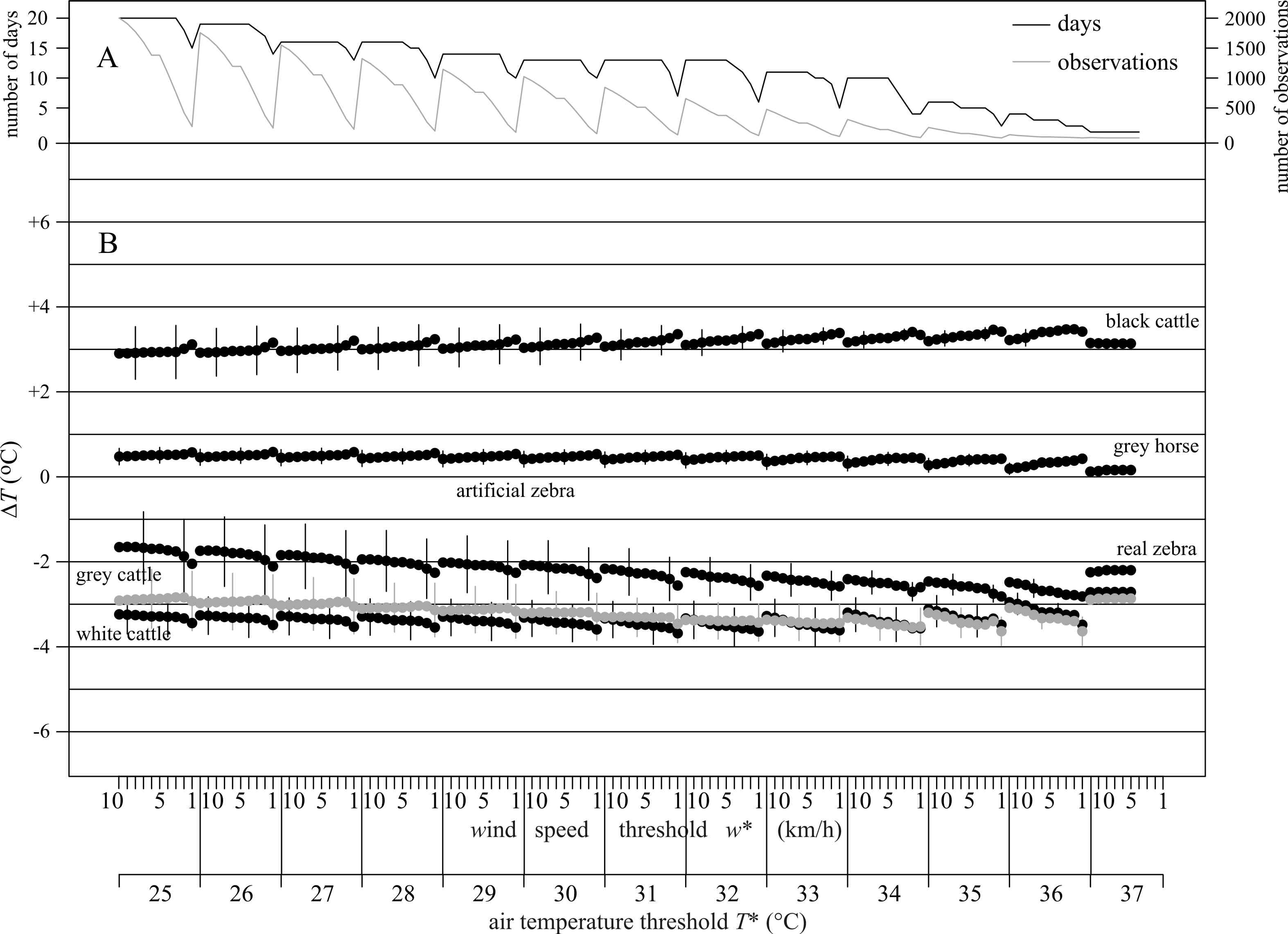


**Supplementary Figure S25**: As Fig. 7 for experiment 3 and time delay Δ*t* = 0 minute.


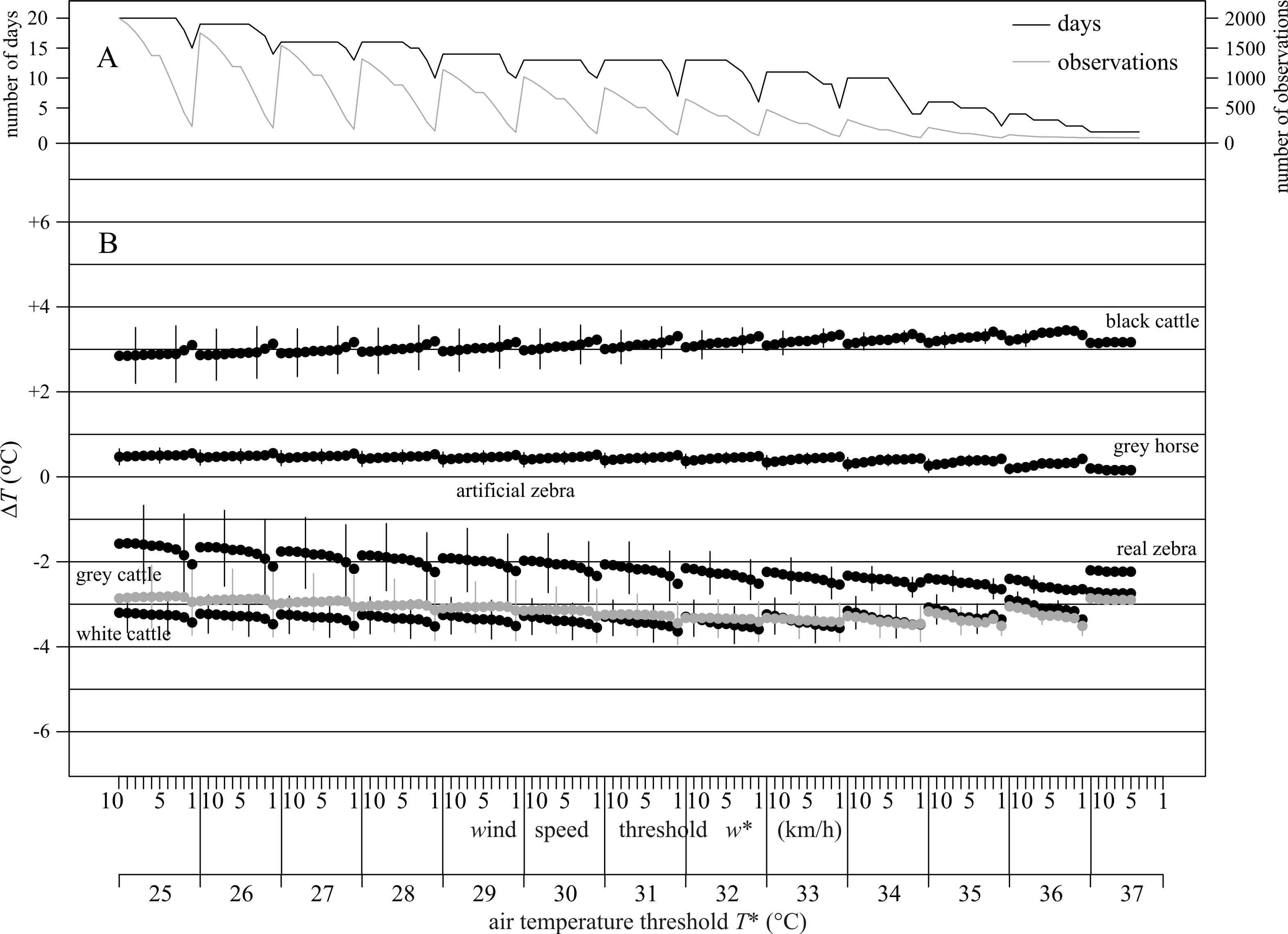


**Supplementary Figure S26**: As Fig. 7 for experiment 3 and time delay Δ*t* = 30 minutes.


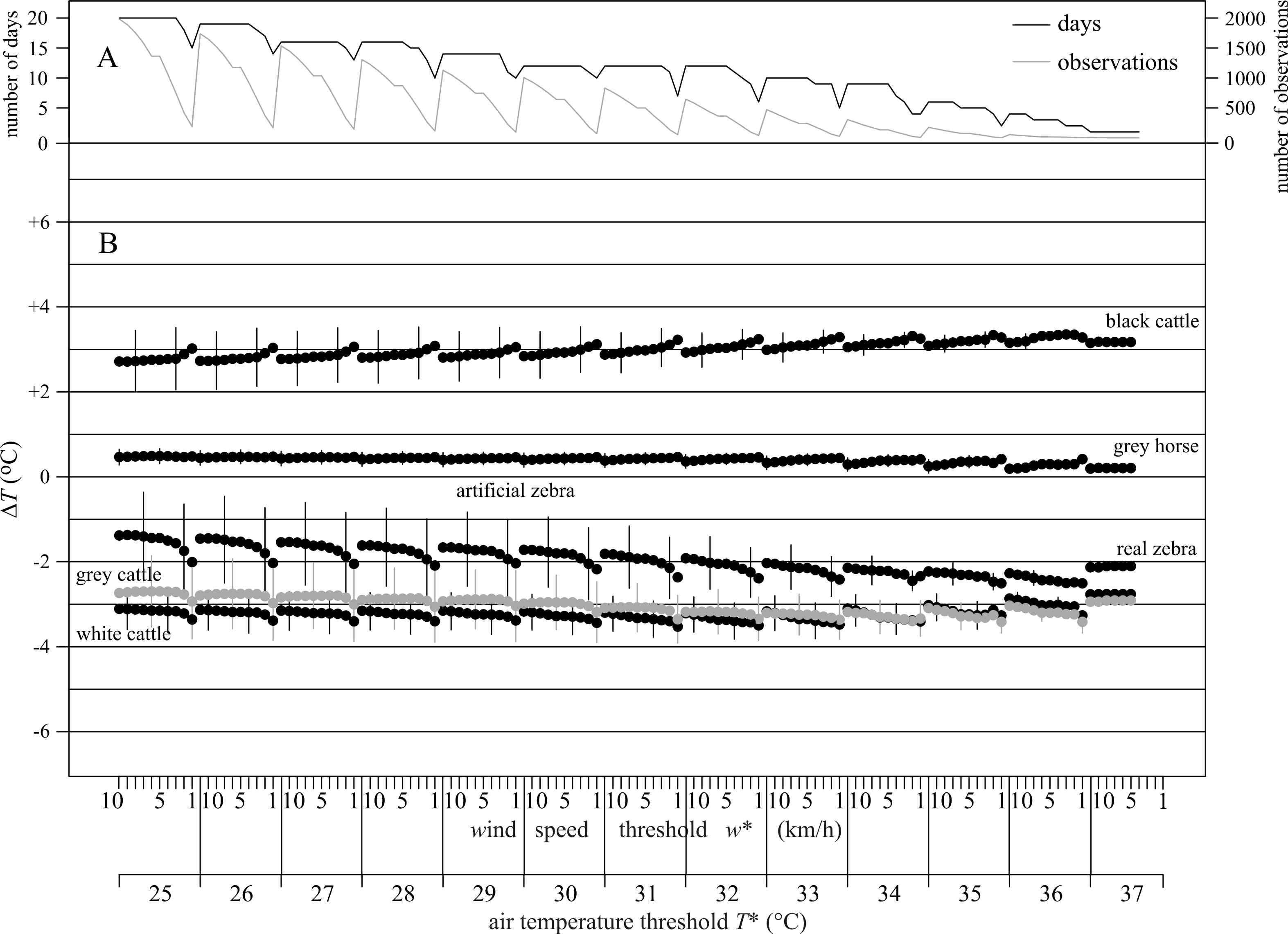


**Supplementary Figure S27**: As Fig. 7 for experiment 3 and time delay Δ*t* = 90 minutes.


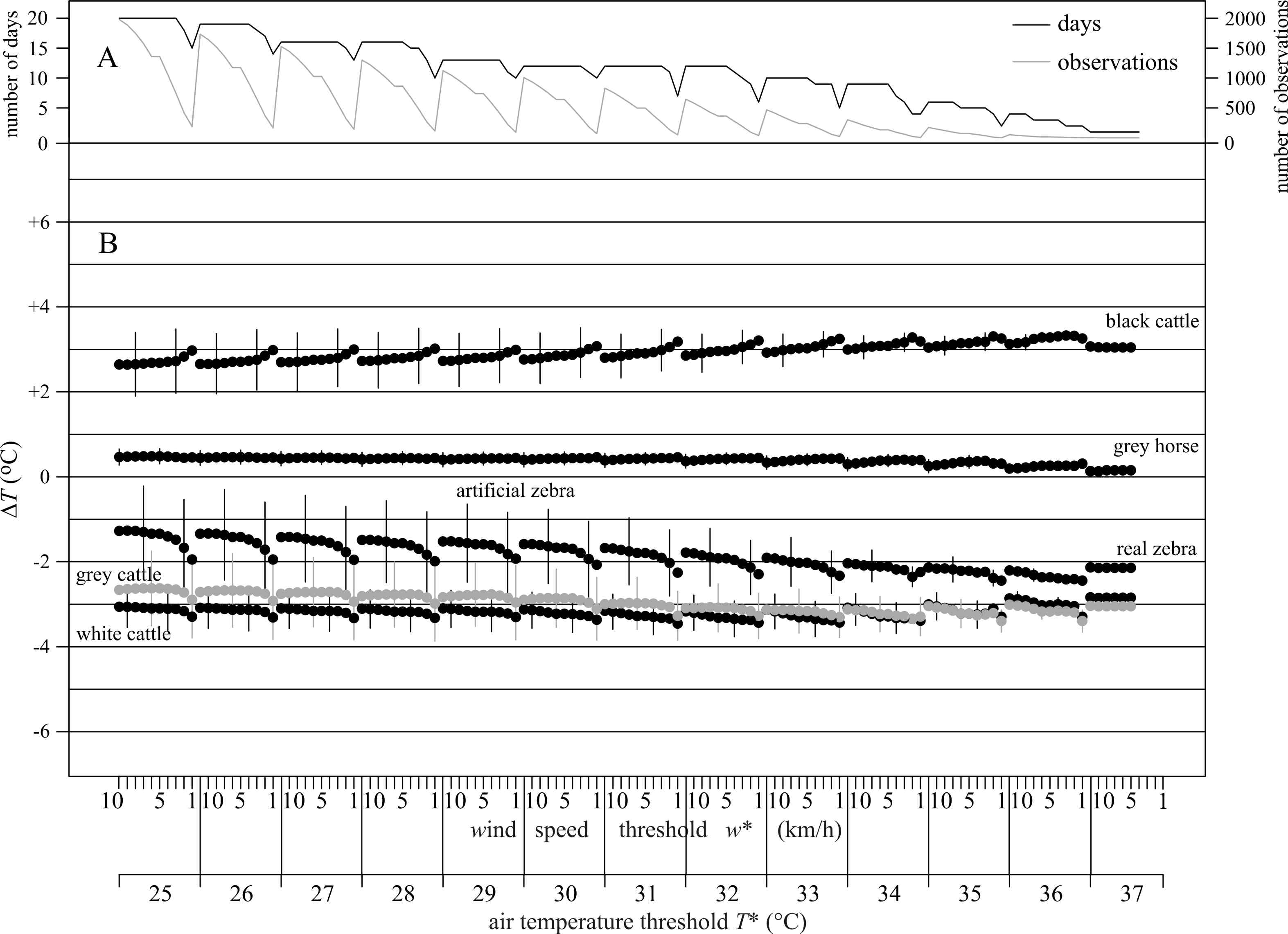


**Supplementary Figure S28**: As Fig. 7 for experiment 3 and time delay Δ*t* = 120 minutes.


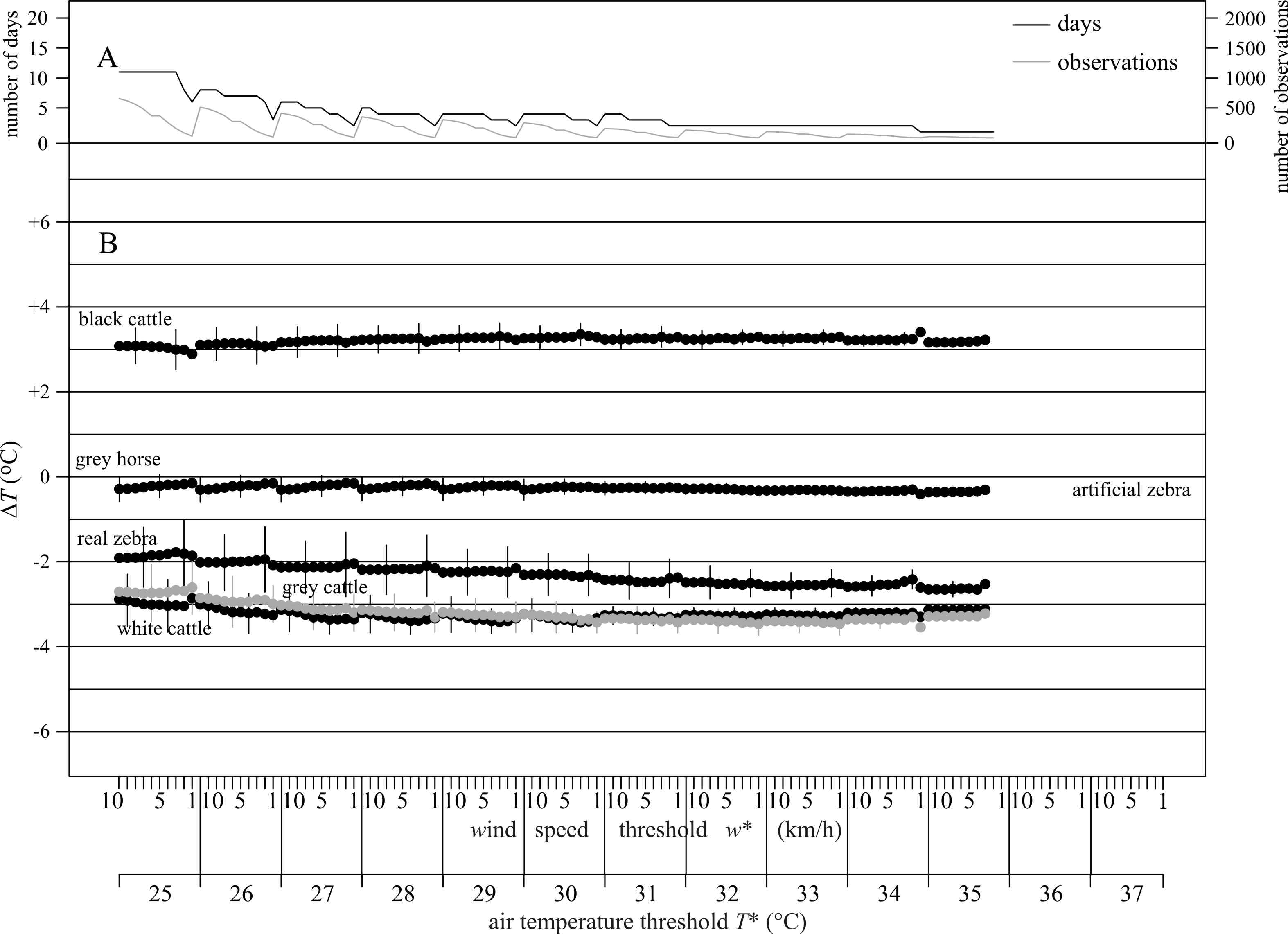


**Supplementary Figure S29**: As Fig. 7 for experiment 4 and time delay Δ*t* = 0 minute.


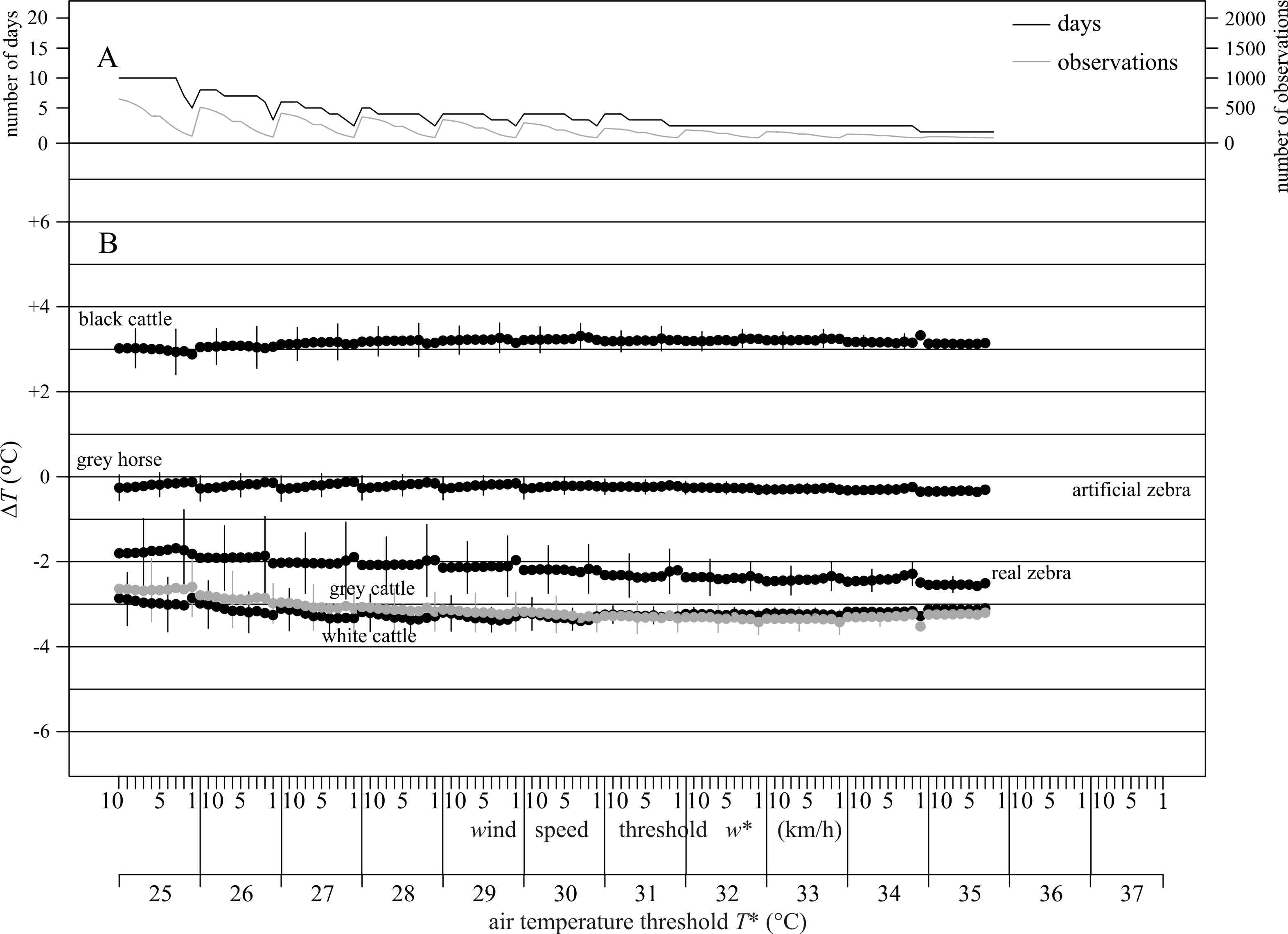


**Supplementary Figure S30**: As Fig. 7 for experiment 4 and time delay Δ*t* = 30 minutes.


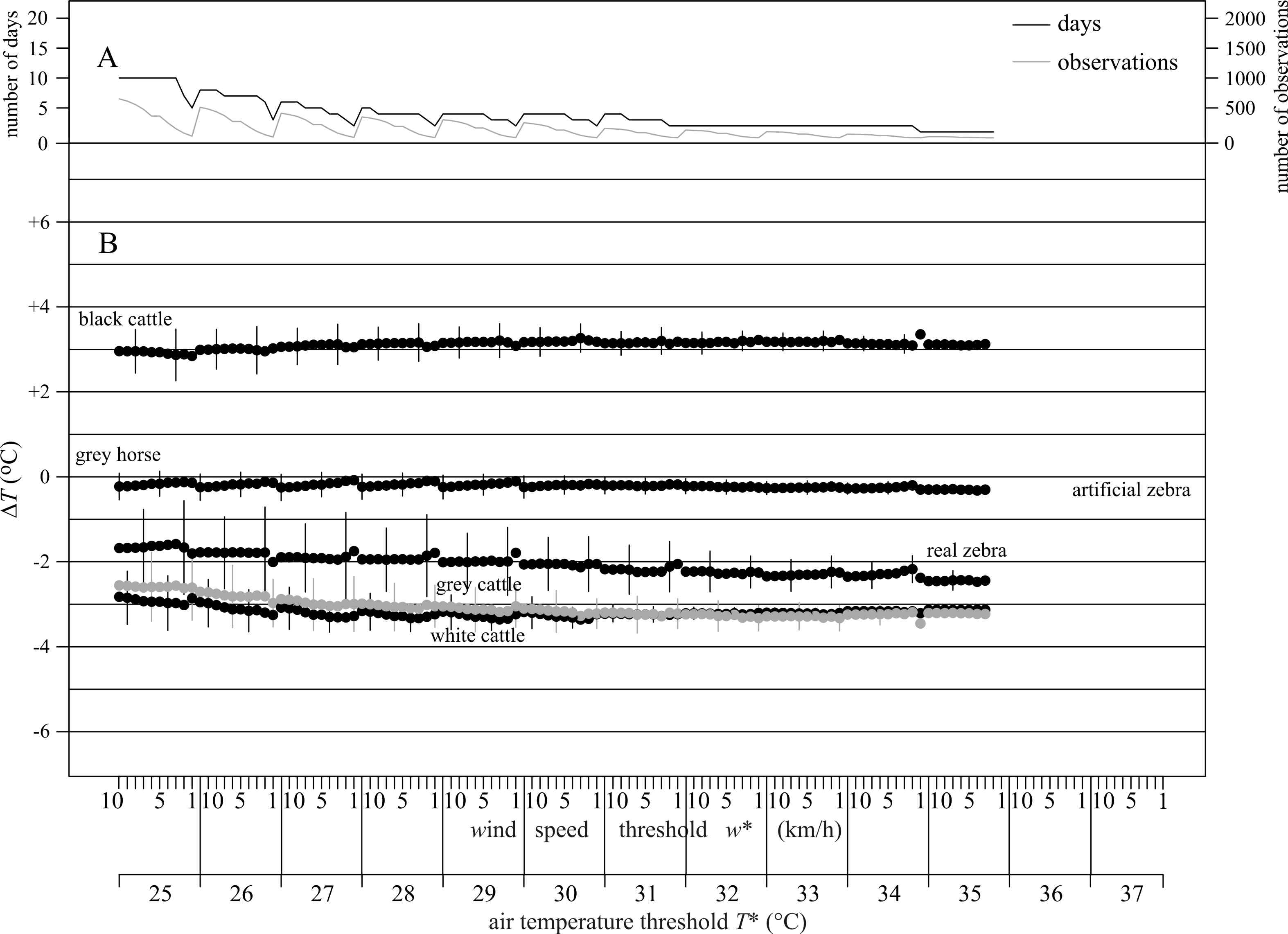


**Supplementary Figure S31**: As Fig. 7 for experiment 4 and time delay Δ*t* = 60 minutes.


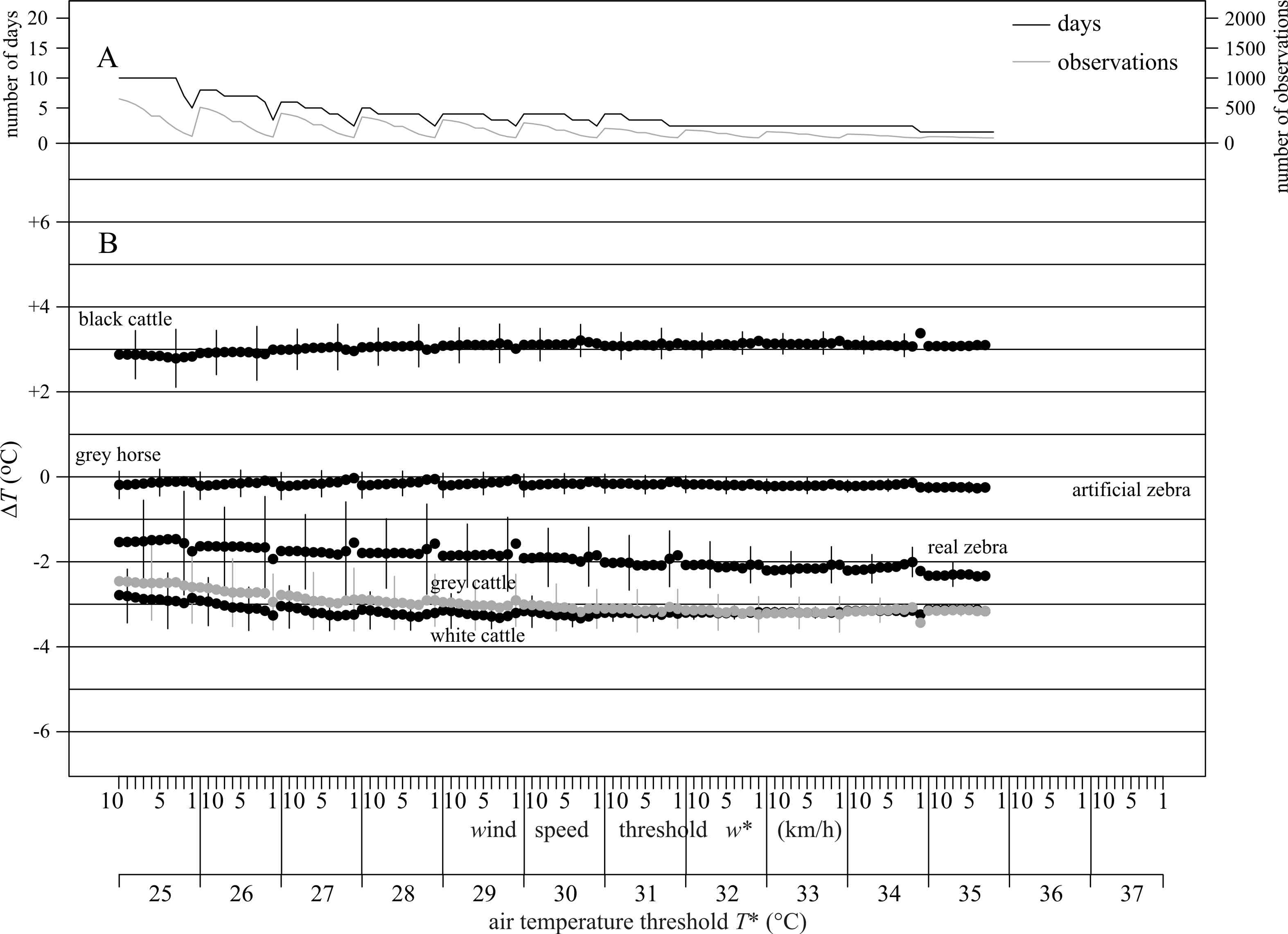


**Supplementary Figure S32**: As Fig. 7 for experiment 4 and time delay Δ*t* = 90 minutes.


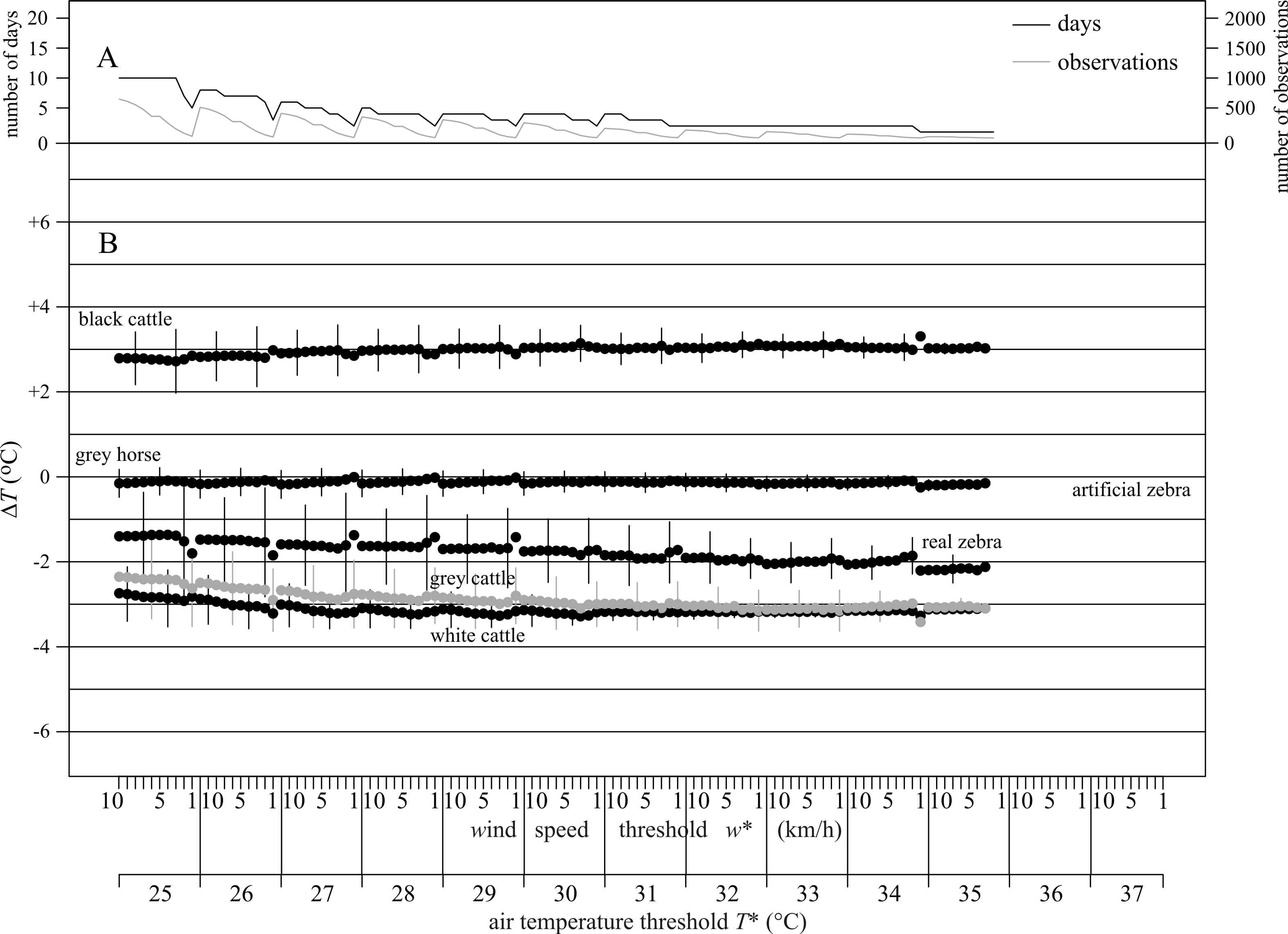


**Supplementary Figure S33**: As Fig. 7 for experiment 4 and time delay Δ*t* = 120 minutes.


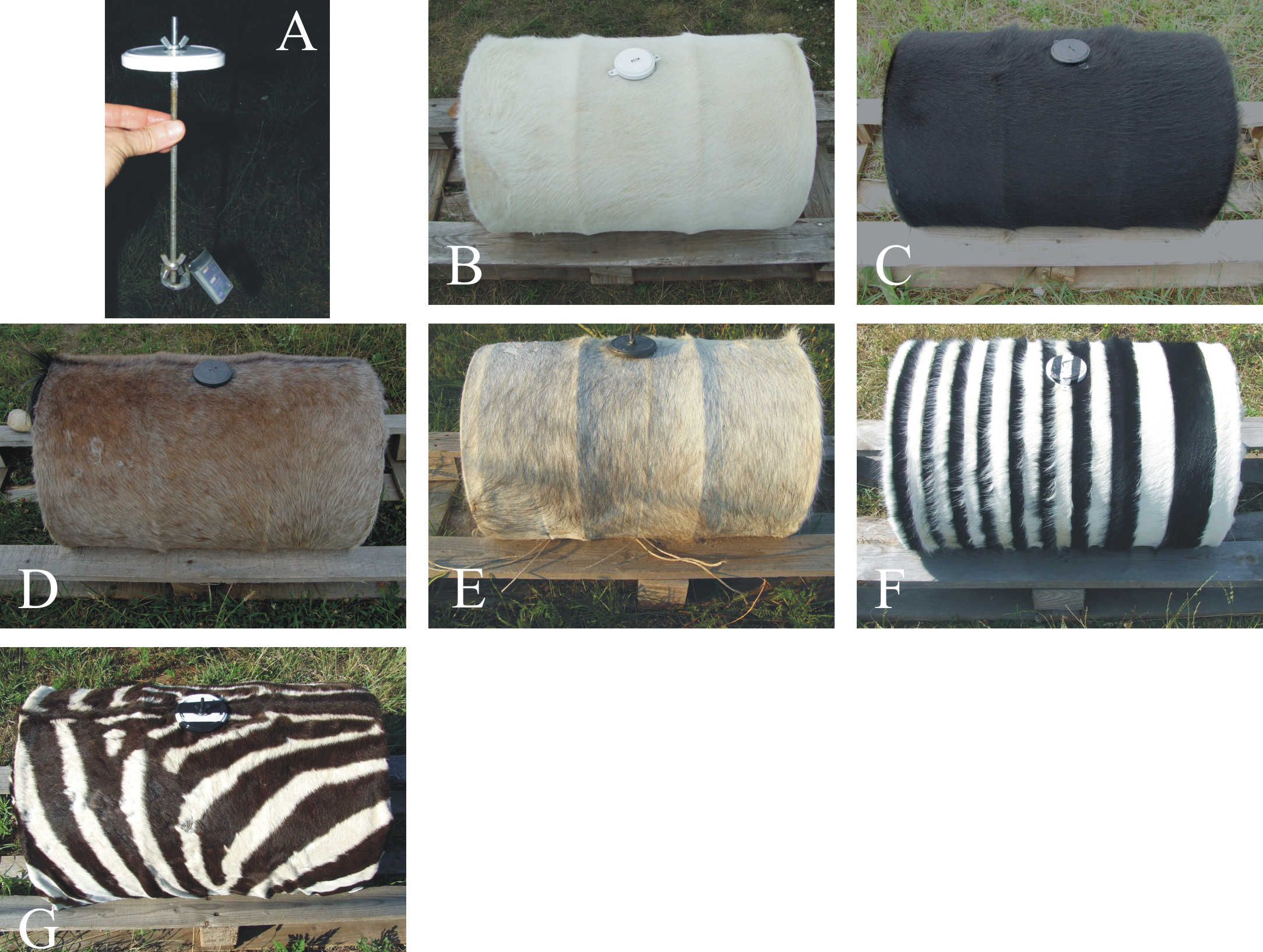


**Supplementary Figure S34**: (A) The thermometer was suspended on the lower end of a vertical metal rod stabilized by a metal weight (nut = 15 gramm). The upper end of the rod was fixed to the cup. (C-H) Barrels covered with white cattle hide (B), black cattle hide (C), brownish-grey horse hide (D), grey cattle hide (E), black-white-striped cattle (artificial zebra) hide (F), and real zebra (*Equus burchelli boehmi*) hide (G).


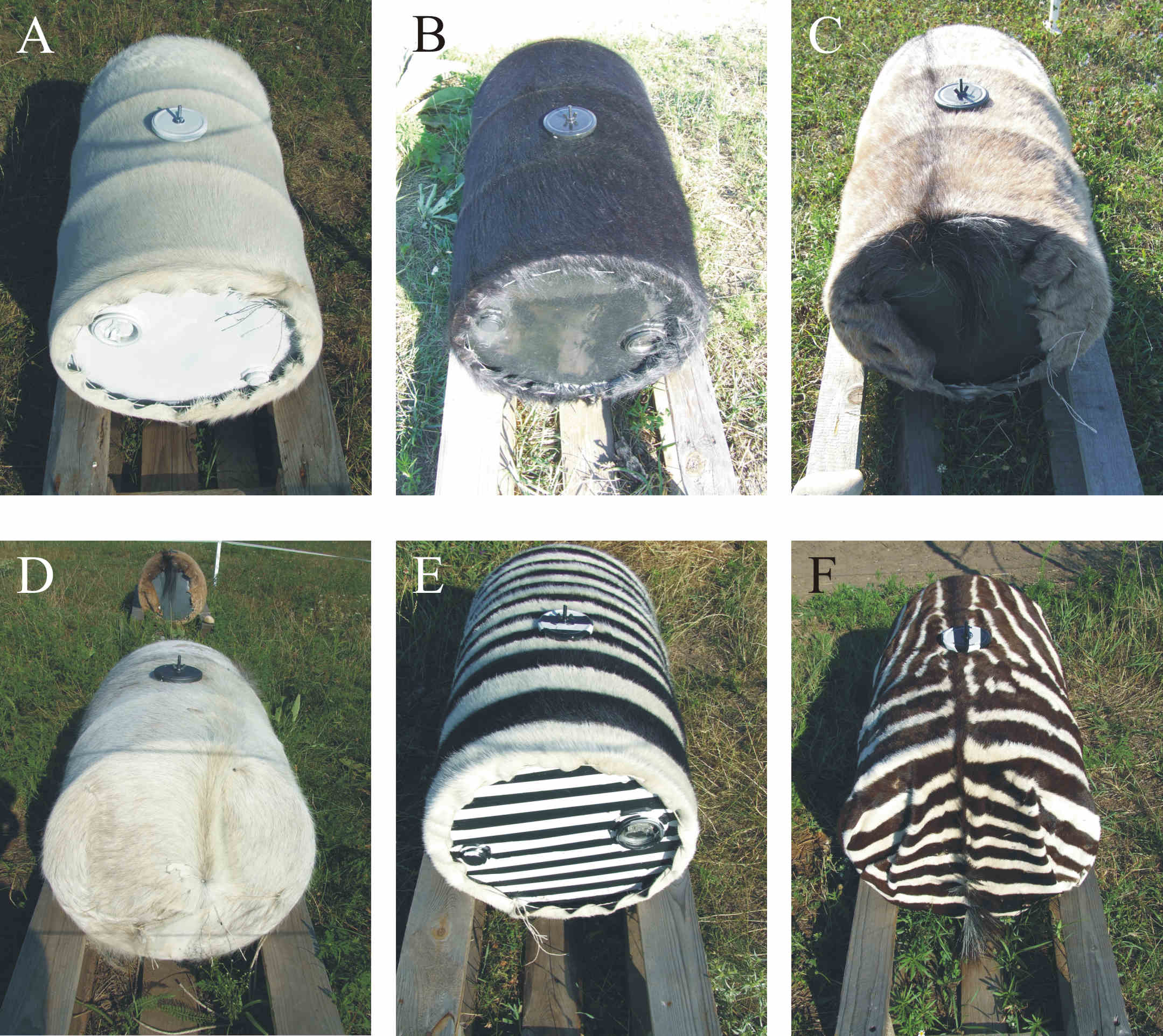


**Supplementary Figure S35**: Side view of the barrels used in the experiments: barrels covered with white cattle hide (A), black cattle hide (B), brownish-grey horse hide (C), grey cattle hide (D), black-white-striped cattle (artificial zebra) hide (E), and real zebra (*Equus burchelli boehmi*) hide (F).


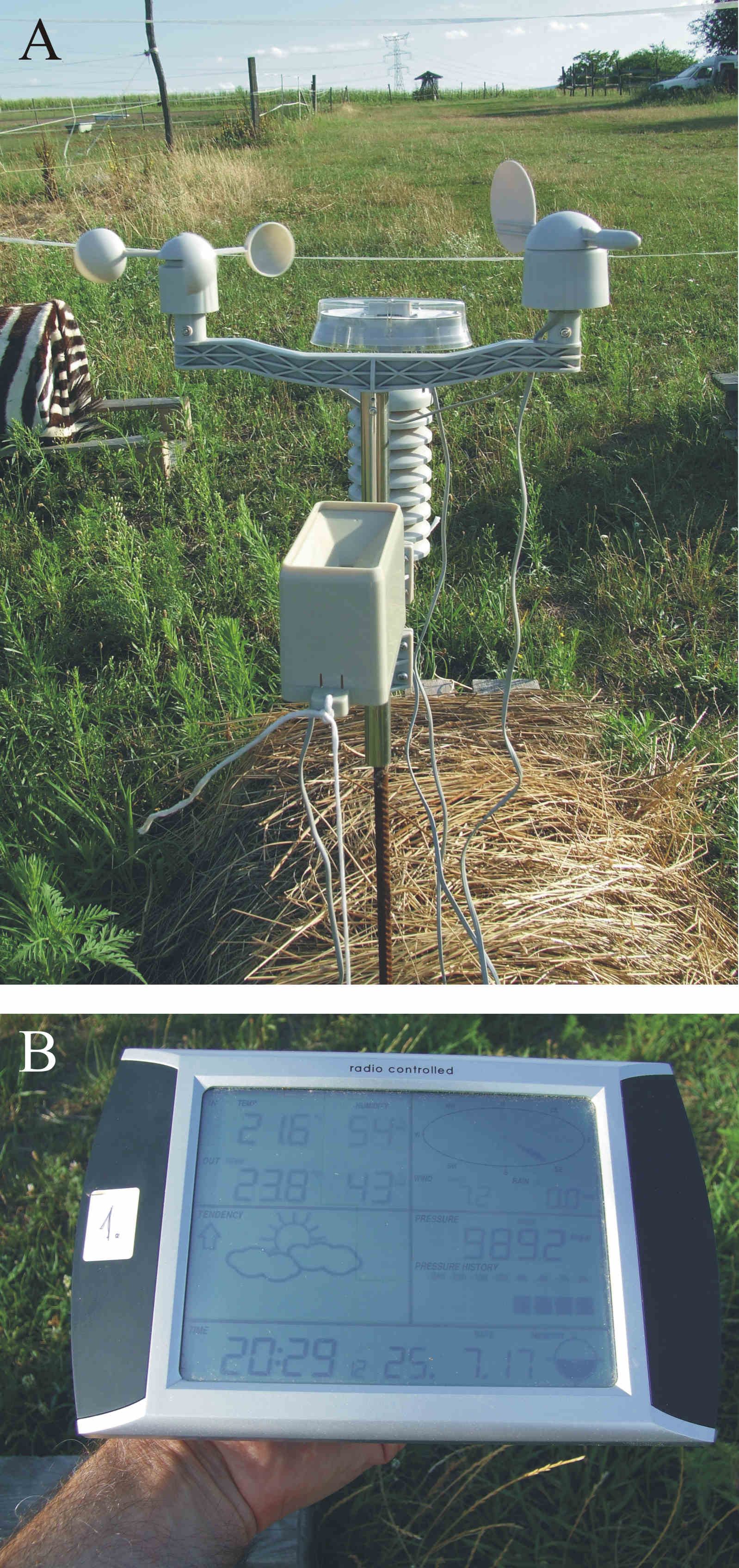


**Supplementary Figure S36**: The automatic meteorological station with sensors fixed at a height of 1 m (A), and the radio-controlled receiver unit registering the weather data (B) used in our four 3-week field experiments.

**Supplementary Table S1**: Average surface temperature *T* (^o^C) ± standard deviation σ_T_ (^o^C) of water-filled barrels covered by homogeneous hides, and average temperature difference Δ*T* (^o^C) between adjacent black and white stripes ± standard deviation σ_ΔT_ (^o^C) of water-filled barrels covered by zebra-striped hides as a function of time measured on 18 July 2017 (experiment 2) with a thermocamera along a straight line on the sunlit top of barrels.

| **18 July 2017** | **white cattle** | | **black cattle** | | **grey horse** | | **artificial zebra** | | **real zebra** | |
| --- | --- | --- | --- | --- | --- | --- | --- | --- | --- | --- |
| **time**  **(h)** | **T**  **(°C)** | **σ_T_**  **(°C)** | **T**  **(°C)** | **σ_T_**  **(°C)** | **T**  **(°C)** | **σ_T_**  **(°C)** | **ΔT**  **(°C)** | **σ_ΔT_**  **(°C)** | **ΔT**  **(°C)** | **σ_ΔT_**  **(°C)** |
| 8 | 24.95 | 0.20 | 37.83 | 1.17 | 32.90 | 0.60 | 6.71 | 2.97 | 6.98 | 1.24 |
| 9 | 28.10 | 0.28 | 44.18 | 0.75 | 37.42 | 0.72 | 7.14 | 3.52 | 8.22 | 1.82 |
| 10 | 31.74 | 0.30 | 48.68 | 1.29 | 40.27 | 0.95 | 10.28 | 1.65 | 6.48 | 2.12 |
| 11 | 34.73 | 0.21 | 53.64 | 1.67 | 47.45 | 0.60 | 10.44 | 1.90 | 8.85 | 2.32 |
| 12 | 35.29 | 0.21 | 54.33 | 3.13 | 48.89 | 1.09 | 11.15 | 3.59 | 12.30 | 3.24 |
| 13 | 37.63 | 0.08 | 60.63 | 2.88 | 54.63 | 1.10 | 8.82 | 2.14 | 16.42 | 3.56 |
| 14 | 37.56 | 0.27 | 60.03 | 2.97 | 56.76 | 1.70 | 8.78 | 2.21 | 11.34 | 2.95 |
| 15 | 38.29 | 0.41 | 66.25 | 1.43 | 56.30 | 1.31 | 10.13 | 3.11 | 10.66 | 3.89 |
| 16 | 37.79 | 0.23 | 56.64 | 1.63 | 53.33 | 1.17 | 7.85 | 3.37 | 13.71 | 1.98 |
| 17 | 37.16 | 0.18 | 56.13 | 1.64 | 49.41 | 0.97 | 7.18 | 0.88 | 7.90 | 2.11 |
| 18 | 34.14 | 0.12 | 49.67 | 1.09 | 42.51 | 1.14 | 5.16 | 0.51 | 5.34 | 1.13 |
| 19 | 32.02 | 0.15 | 43.20 | 0.59 | 39.17 | 0.45 | 5.00 | 1.58 | 3.89 | 1.07 |

**Supplementary Table S2**: As Supplementary Table S1 measured on 30 July 2017 (experiment 2).

| **30 July 2017** | **white cattle** | | **black cattle** | | **grey horse** | | **artificial zebra** | | **real zebra** | |
| --- | --- | --- | --- | --- | --- | --- | --- | --- | --- | --- |
| **time**  **(h)** | **T**  **(°C)** | **σ_T_**  **(°C)** | **T**  **(°C)** | **σ_T_**  **(°C)** | **T**  **(°C)** | **σ_T_**  **(°C)** | **ΔT**  **(°C)** | **σ_ΔT_**  **(°C)** | **ΔT**  **(°C)** | **σ_ΔT_**  **(°C)** |
| 9 | 28.55 | 0.31 | 43.71 | 0.79 | 36.13 | 1.02 | 6.98 | 1.76 | 5.41 | 1.09 |
| 10 | 31.37 | 0.33 | 47.03 | 1.69 | 41.27 | 0.65 | 6.04 | 2.78 | 7.35 | 0.93 |
| 11 | 33.90 | 0.36 | 49.61 | 0.79 | 43.82 | 0.67 | 7.08 | 2.91 | 7.15 | 1.15 |
| 12 | 36.77 | 0.25 | 51.32 | 0.79 | 45.89 | 0.82 | 7.27 | 2.39 | 7.90 | 1.25 |
| 13 | 40.31 | 0.40 | 59.64 | 1.78 | 52.49 | 1.13 | 11.72 | 3.44 | 16.22 | 3.60 |
| 14 | 39.13 | 0.41 | 60.18 | 1.84 | 54.58 | 0.76 | 9.47 | 3.49 | 17.08 | 3.02 |
| 15 | 40.49 | 0.83 | 55.54 | 2.56 | 53.97 | 1.30 | 8.94 | 1.89 | 15.06 | 2.67 |
| 16 | 39.35 | 0.80 | 57.79 | 1.15 | 52.70 | 1.24 | 5.74 | 2.59 | 12.32 | 1.48 |
| 17 | 38.06 | 0.68 | 49.50 | 1.73 | 47.07 | 1.10 | 6.78 | 3.19 | 9.57 | 1.37 |
| 18 | 36.11 | 0.34 | 50.42 | 1.21 | 47.42 | 0.67 | 6.46 | 2.56 | 8.28 | 1.83 |
| 19 | 31.94 | 0.28 | 42.05 | 0.65 | 38.66 | 0.49 | 3.03 | 1.74 | 1.01 | 0.27 |

**Supplementary Table S3**: As Supplementary Table S1 measured on 15 August 2017 (experiment 3).

| **15 August 2017** | **white cattle** | | **black cattle** | | **grey horse** | | | **grey cattle** | | **artificial zebra** | | **real zebra** | |
| --- | --- | --- | --- | --- | --- | --- | --- | --- | --- | --- | --- | --- | --- |
| **time**  **(h)** | **T**  **(°C)** | **σ_T_**  **(°C)** | **T**  **(°C)** | **σ_T_**  **(°C)** | | **T**  **(°C)** | **σ_T_**  **(°C)** | **T**  **(°C)** | **σ_T_**  **(°C)** | **ΔT**  **(°C)** | **σ_ΔT_**  **(°C)** | **ΔT**  **(°C)** | **σ_ΔT_**  **(°C)** |
| 8 | 25.23 | 0.17 | 36.31 | 0.99 | | 30.63 | 0.56 | 26.46 | 0.52 | 4.82 | 2.14 | 6.39 | 0.30 |
| 9 | 27.12 | 0.38 | 38.82 | 0.93 | | 33.30 | 0.52 | 27.82 | 0.33 | 5.70 | 2.13 | 6.56 | 1.32 |
| 10 | 30.17 | 0.32 | 43.97 | 0.82 | | 37.19 | 0.73 | 30.13 | 0.67 | 6.11 | 2.22 | 6.57 | 0.74 |
| 11 | 32.28 | 0.24 | 48.23 | 0.74 | | 40.98 | 0.79 | 33.19 | 0.44 | 5.92 | 2.61 | 7.21 | 0.98 |
| 12 | 37.93 | 0.57 | 60.05 | 1.67 | | 51.51 | 0.81 | 37.77 | 0.47 | 7.38 | 2.71 | 14.87 | 2.70 |
| 13 | 39.37 | 0.56 | 62.73 | 1.87 | | 53.81 | 0.89 | 41.09 | 0.37 | 8.37 | 1.46 | 14.53 | 4.92 |
| 14 | 40.96 | 0.75 | 65.51 | 1.19 | | 55.59 | 0.87 | 42.60 | 0.33 | 7.31 | 2.28 | 17.90 | 3.31 |
| 15 | 39.76 | 0.41 | 60.12 | 1.83 | | 53.45 | 0.47 | 40.99 | 0.35 | 6.84 | 2.23 | 15.12 | 3.96 |
| 16 | 38.98 | 0.57 | 58.73 | 1.15 | | 51.87 | 0.88 | 41.61 | 0.40 | 4.14 | 1.77 | 14.84 | 3.69 |
| 17 | 36.22 | 0.44 | 55.11 | 0.63 | | 48.38 | 0.72 | 39.67 | 0.30 | 6.17 | 2.62 | 11.14 | 3.15 |
| 18 | 33.57 | 0.24 | 46.85 | 0.63 | | 42.07 | 0.61 | 36.65 | 0.20 | 4.38 | 1.65 | 5.73 | 1.80 |
| 19 | 32.13 | 0.20 | 43.32 | 0.62 | | 38.88 | 0.66 | 34.71 | 0.14 | 3.19 | 1.30 | 4.73 | 1.88 |

**Supplementary Table S4**: Average of the temperature difference Δ*T* (^o^C) ± standard deviation σ_Δ_*_T_* (^o^C) between adjacent black and white stripes of zebras (*Equus burchelli boehmi*) in the Budapest Zoo & Botanical Garden. Δ*T* ± σ_Δ_*_T_* is measured along straight lines marked with arrows in the thermograms of Fig. 5. The target column indicates whether the arrow runs on a sunlit or a shady region of the zebra body.

|  | **target** | **Δ*T* (^o^C)** | **σ_Δ_*_T_* (^o^C)** |
| --- | --- | --- | --- |
| **zebras**  (*Equus burchelli boehmi*),  **Figure 5** | Fig. 5A, sunlit | 2.44 | 1.37 |
|  | Fig. 5A, shady | 0.93 | 0.51 |
|  | Fig. 5B, sunlit | 5.42 | 1.16 |
|  | Fig. 5B, shady | 4.00 | 1.32 |
|  | Fig. 5C, sunlit | 2.83 | 0.83 |
|  | Fig. 5C, shady | 3.13 | 1.69 |
|  | Fig. 5D, sunlit | 3.49 | 2.22 |
|  | Fig. 5D, shady | 4.65 | 1.30 |
|  | Fig. 5E, sunlit | 3.78 | 1.21 |
|  | Fig. 5E, shady | 1.98 | 0.64 |
|  | Fig. 5F, sunlit | 4.54 | 1.18 |
|  | Fig. 5F, sunlit | 3.37 | 0.41 |
|  | Fig. 5F, shady | 0.50 | 0.15 |
|  | Fig. 5G, sunlit | 3.03 | 1.13 |
|  | Fig. 5G, shady | 0.64 | 0.59 |
|  | Fig. 5H, shady | 1.09 | 0.60 |
|  | Fig. 5H, shady | 1.09 | 0.42 |

**Supplementary Table S5**: Integral $INT=\int_{350\text{nm}}^{825\text{nm}} I(\text{λ})\text{dλ}$ of the spectrum *I*(λ) and the whiteness *wh* = *INT*/*INT*_white cattle_ of the different hides covering the barrel surfaces in our experiments, where *I* and λ are the radiance and wavelength of surface-reflected light, respectively (Supplementary Fig. S10).

| **barrel surface** | ***INT*** | ***wh*** |
| --- | --- | --- |
| white cattle | 2240227.5304 | 1.00 |
| real zebra (white stripe) | 2103787.015 | 0.94 |
| grey cattle | 1228327.0256 | 0.55 |
| real zebra average | 1185550.2877 | 0.53 |
| artificial zebra average | 958525.8263 | 0.43 |
| grey horse | 568491.3338 | 0.25 |
| real zebra (black stripe) | 267313.5577 | 0.12 |
| black cattle | 74816.114 | 0.03 |

**Supplementary Table S6**: Time delay Δ*t* = δ* (minute) for which the cross correlation integral $CC(\text{δ})=\int_{t\min}^{t\max} T_{\text{air}}(\text{τ})T_{\text{core}}(\text{τ}+\text{δ})\text{dτ}$ of the measured time-dependent air temperatue *T*_air_(*t*) and core temperature *T*_core_(*t*) of a given barrel is maximal calculated for the warmest days (28 June, 20 July, 10 August, 1 September) of experiments 1-4 between *t*_min_ = 6:00 and *t*_max_ = 20:00 h (UTC + 2 h). The maximum of *CC*(δ) at time lag δ^*^ provides an estimate for the thermal response time as Δ*t* = δ^*^ with which *T*_core_(*t*) follows the changes in *T*_air_(*t*).

| **datum (2017) of**  **the warmest day** | **experiment** | **barrel** | | | | | |
| --- | --- | --- | --- | --- | --- | --- | --- |
|  |  | **white**  **cattle** | **black**  **cattle** | **real**  **zebra** | **grey**  **horse** | **grey**  **cattle** | **artificial**  **zebra** |
| 28 June | 1 | 50 | 15 | - | 55 | - | 65 |
| 20 July | 2 | 35 | 15 | 40 | 20 | - | 20 |
| 10 August | 3 | 60 | 40 | 70 | 55 | 65 | 55 |
| 1 September | 4 | 105 | 80 | 120 | 100 | 120 | 95 |

**Supplementary Table S7**: Average ± standard deviation of the core temperature *T*_core_ of different barrels calculated only for hot days when the average air temperature was higher than 25 °C (above which cooling air eddies above zebras are the most likely to form) between 12:00 and 18:00 h (UTC + 2 h) in June (experiment 1), July (experiment 2), August (experiment 3), and between 12:00 and 17:00 h in September (experiment 4) in 2017.

| **experiment** | **white**  **cattle** | **black**  **cattle** | **real**  **zebra** | **grey**  **horse** | **grey**  **cattle** | **artifical**  **zebra** |
| --- | --- | --- | --- | --- | --- | --- |
| **1 (June)** | 28.18 ±3.28 | 35.89 ±4.62 | - | 32.16 ±3.86 | - | 31.43 ±4.05 |
| **2 (July)** | 27.99 ±2.53 | 33.7 ±2.73 | 29.38 ±2.41 | 31.97 ±2.72 | - | 31.69 ±2.77 |
| **3 (August)** | 28.18 ±3.79 | 34.68 ±3.65 | 29.51 ±3.66 | 31.92 ±3.76 | 28.27 ±3.64 | 31.52 ±3.78 |
| **4 (September)** | 22.94 ±3.3 | 28.74 ±3.81 | 23.66 ±3.27 | 25.25 ±3.63 | 22.95 ±3.17 | 25.59 ±3.66 |

**Supplementary Table S8**: Average ± standard deviation of the core temperature *T*_core_ of different barrels calculated for all days between 12:00 and 18:00 h (UTC + 2 h) in June (experiment 1), July (experiment 2), August (experiment 3), and between 12:00 and 17:00 h in September (experiment 4) in 2017.

| **experiment** | **white**  **cattle** | **black**  **cattle** | **real**  **zebra** | **grey**  **horse** | **grey**  **cattle** | **artifical**  **zebra** |
| --- | --- | --- | --- | --- | --- | --- |
| **1 (June)** | 27.44 ±3.58 | 35.15 ±4.69 | - | 31.54 ±3.97 | - | 30.87 ±4.07 |
| **2 (July)** | 26.09 ±3.89 | 31.49 ±4.63 | 27.69 ±3.65 | 29.95 ±4.26 | - | 29.47 ±4.52 |
| **3 (August)** | 26.00 ±4.68 | 31.96 ±5.36 | 27.56 ±4.35 | 29.56 ±4.88 | 26.25 ±4.43 | 29.08 ±4.99 |
| **4 (September)** | 19.62 ±3.81 | 23.94 ±5.30 | 20.67 ±3.76 | 21.6 ±4.28 | 19.91 ±3.70 | 21.64 ±4.48 |

**Supplementary Table S9**: Average ± standard deviation of the air temperature calculated for all days between 12:00 and 18:00 h (UTC + 2 h) in June (experiment 1), July (experiment 2), August (experiment 3), and between 12:00 and 17:00 h in September (experiment 4) in 2017.

| **experiment 1**  **(June)** | | **experiment 2**  **(July)** | | **experiment 3**  **(August)** | | **experiment 4**  **(September)** | |
| --- | --- | --- | --- | --- | --- | --- | --- |
| 10 June | 23.38 ± 2.64 | 6 July | 31.02 ± 0.41 | 6 August | 32.19 ± 2.74 | 30 August | 29.8 ± 0.86 |
| 11 | 29.34 ± 1.16 | 7 | 28.76 ± 1.69 | 7 | 23.31 ± 1.4 | 31 August | 32.98 ± 1.09 |
| 12 | 31.07 ± 1.04 | 8 | 32.03 ± 1.16 | 8 | 27.38 ± 0.91 | 1 September | 34.48 ± 0.74 |
| 13 | 28.75 ± 0.58 | 9 | 31.96 ± 1.5 | 9 | 33.74 ± 0.86 | 2 | 24.04 ± 0.59 |
| 14 | 26.49 ± 0.86 | 10 | 32.13 ± 1.72 | 10 | 36.08 ± 0.79 | 3 | 17.71 ± 1.21 |
| 15 | 29.33 ± 1.21 | 11 | 29.72 ± 0.68 | 11 | 35.36 ± 0.54 | 4 | 21.78 ± 1.09 |
| 16 | 23.79 ± 2.21 | 12 | 30.78 ± 0.55 | 12 | 26.6 ± 0.99 | 5 | 22.8 ± 1.32 |
| 17 | 20.65 ± 0.96 | 13 | 24.21 ± 0.65 | 13 | 24.56 ± 1.92 | 6 | 25.36 ± 0.85 |
| 18 | 26.68 ± 1.03 | 14 | 23.63 ± 1.22 | 14 | 30.53 ± 0.86 | 7 | 20.05 ± 2.02 |
| 19 | 31.33 ± 1.05 | 15 | 22.8 ± 1.6 | 15 | 32.14 ± 0.87 | 8 | 25.37 ± 0.63 |
| 20 | 33.86 ± 0.69 | 16 | 26.53 ± 1.1 | 16 | 33.59 ± 0.92 | 9 | 26.33 ± 0.76 |
| 21 | 33.71 ± 0.78 | 17 | 30 ± 1.18 | 17 | 34.29 ± 1.35 | 10 | 29.89 ± 0.85 |
| 23 | 32.21 ± 0.62 | 18 | 32.78 ± 0.88 | 19 | 30.91 ± 2.18 | 12 | 18.51 ± 0.95 |
| 24 | 32.55 ± 1.33 | 20 | 35.92 ± 0.75 | 20 | 23.46 ± 1.13 | 13 | 22.77 ± 0.95 |
| 25 | 30.73 ± 1.76 | 21 | 32.77 ± 0.63 | 21 | 24.22 ± 0.99 | 14 | 26.32 ± 0.69 |
| 26 | 29.99 ± 1.54 | 22 | 29.98 ± 3.11 | 22 | 24.84 ± 0.83 | 15 | 20.41 ± 1.27 |
| 27 | 32.78 ± 0.71 | 23 | 31.17 ± 1.98 | 23 | 24.73 ± 0.97 | 16 | 23.42 ± 1.08 |
| 28 | 34.85 ± 0.82 | 24 | 29.06 ± 2.91 | 24 | 29.06 ± 1.31 | 17 | 17.76 ± 0.37 |
| 29 | 28.55 ± 1.32 | 25 | 23.36 ± 0.69 | 25 | 31.12 ± 1.55 | 18 | 22.33 ± 1.15 |
| 30 June | 30.22 ± 0.76 | 26 | 22.14 ± 2.75 | 26 | 35.07 ± 1.1 | 19 | 15.06 ± 0.75 |
| 1 July | 27.09 ± 1.93 | 27 | 21.13 ± 1.45 | - | - | 20 | 17.58 ± 0.87 |

**Supplementary Table S10**: Results of Bonferroni post-hoc tests for all days between 12:00 and 18:00 h (UTC + 2 h) in June (experiment 1), July (experiment 2), August (experiment 3), and between 12:00 and 17:00 h in September (experiment 4) in 2017.

| **experiment 1**  **(June)** |  | **grey horse** | **artifical zebra** |  |  |
| --- | --- | --- | --- | --- | --- |
|  | **grey horse** |  | 1.0000 |  |  |
|  | **artifical zebra** | 1.0000 |  |  |  |
| **experiment 2**  **(July)** |  | **real zebra** | **grey horse** | **artifical zebra** |  |
|  | **real zebra** |  | 0.7475 | 1.0000 |  |
|  | **grey horse** | 0.7475 |  | 1.0000 |  |
|  | **artifical zebra** | 1.0000 | 1.0000 |  |  |
| **experiment 3**  **(August)** |  | **real zebra** | **grey horse** | **grey cattle** | **artifical zebra** |
|  | **real zebra** |  | 1.0000 | 1.0000 | 1.0000 |
|  | **grey horse** | 1.0000 |  | 0.3637 | 1.0000 |
|  | **grey cattle** | 1.0000 | 0.3637 |  | 0.8031 |
|  | **artifical zebra** | 1.0000 | 1.0000 | 0.8031 |  |
| **experiment 4**  **(September)** |  | **real zebra** | **grey horse** | **grey cattle** | **artifical zebra** |
|  | **real zebra** |  | 1.0000 | 1.0000 | 1.0000 |
|  | **grey horse** | 1.0000 |  | 1.0000 | 1.0000 |
|  | **grey cattle** | 1.0000 | 1.0000 |  | 1.0000 |
|  | **artifical zebra** | 1.0000 | 1.0000 | 1.0000 |  |

**Supplementary Table S11**: Results of Bonferroni post-hoc tests for hot days when the average air temperature was higher than 25 °C (above which cooling air eddies above zebras are the most likely to form) between 12:00 and 18:00 h (UTC + 2 h) in June (experiment 1), July (experiment 2), August (experiment 3), and between 12:00 and 17:00 h in September (experiment 4) in 2017.

| **experiment 1**  **(June)** |  | **grey horse** | **artifical zebra** |  |  |
| --- | --- | --- | --- | --- | --- |
|  | **grey horse** |  | 1.0000 |  |  |
|  | **artifical zebra** | 1.0000 |  |  |  |
| **experiment 2**  **(July)** |  | **real zebra** | **grey horse** | **artifical zebra** |  |
|  | **real zebra** |  | 0.1077 | 0.3178 |  |
|  | **grey horse** | 0.1077 |  | 1.0000 |  |
|  | **artifical zebra** | 0.3178 | 1.0000 |  |  |
| **experiment 3**  **(August)** |  | **real zebra** | **grey horse** | **grey cattle** | **artifical zebra** |
|  | **real zebra** |  | 0.8973 | 1.0000 | 1.0000 |
|  | **grey horse** | 0.8973 |  | 0.0749 | 1.0000 |
|  | **grey cattle** | 1.0000 | 0.0749 |  | 0.0997 |
|  | **artifical zebra** | 1.0000 | 1.0000 | 0.0997 |  |
| **experiment 4**  **(September)** |  | **real zebra** | **grey horse** | **grey cattle** | **artifical zebra** |
|  | **real zebra** |  | 1.0000 | 1.0000 | 1.0000 |
|  | **grey horse** | 1.0000 |  | 1.0000 | 1.0000 |
|  | **grey cattle** | 1.0000 | 1.0000 |  | 1.0000 |
|  | **artifical zebra** | 1.0000 | 1.0000 | 1.0000 |  |
